# Structural Investigations on 2-Amidobenzimidazole Derivatives as New Inhibitors of Protein Kinase CK1 Delta

**DOI:** 10.3390/ph17040468

**Published:** 2024-04-07

**Authors:** Sara Calenda, Daniela Catarzi, Flavia Varano, Erica Vigiani, Rosaria Volpini, Catia Lambertucci, Andrea Spinaci, Letizia Trevisan, Ilenia Grieco, Stephanie Federico, Giampiero Spalluto, Gianluca Novello, Veronica Salmaso, Stefano Moro, Vittoria Colotta

**Affiliations:** 1 Section of Pharmaceutical and Nutraceutical Sciences, Department of Neuroscience, Psychology, Drug Research and Child Health (NEUROFARBA), University of Florence, Via Ugo Schiff, 6, 50019 Florence, Italy; sara.calenda@unifi.it (S.C.); daniela.catarzi@unifi.it (D.C.); flavia.varano@unifi.it (F.V.); erica.vigiani@unifi.it (E.V.); 2 Medicinal Chemistry Unit, School of Pharmacy, University of Camerino, Via Madonna delle Carceri, 62032 Camerino, Italy; rosaria.volpini@unicam.it (R.V.); catia.lambertucci@unicam.it (C.L.); andrea.spinaci@unicam.it (A.S.); 3 Department of Chemical and Pharmaceutical Sciences, University of Trieste, Via Licio Giorgieri 1, 34127 Trieste, Italy; letizia.trevisan@phd.units.it (L.T.); ilenia.grieco@phd.units.it (I.G.); sfederico@units.it (S.F.); spalluto@units.it (G.S.); 4 Molecular Modeling Section (MMS), Department of Pharmaceutical and Pharmacological Sciences, University of Padova, Via Marzolo 5, 35131 Padova, Italy; gianluca.novello@phd.unipd.it (G.N.); veronica.salmaso@unipd.it (V.S.); stefano.moro@unipd.it (S.M.)

**Keywords:** CK1δ inhibitors, benzimidazole derivatives, molecular modeling

## Abstract

Protein kinase CK1δ (CK1δ) is a serine-threonine/kinase that modulates different physiological processes, including the cell cycle, DNA repair, and apoptosis. CK1δ overexpression, and the consequent hyperphosphorylation of specific proteins, can lead to sleep disorders, cancer, and neurodegenerative diseases. CK1δ inhibitors showed anticancer properties as well as neuroprotective effects in cellular and animal models of Parkinson’s and Alzheimer’s diseases and amyotrophic lateral sclerosis. To obtain new ATP-competitive CK1δ inhibitors, three sets of benzimidazole-2-amino derivatives were synthesized (**1**–**32**), bearing different substituents on the fused benzo ring (R) and diverse pyrazole-containing acyl moieties on the 2-amino group. The best-performing derivatives were those featuring the (1H-pyrazol-3-yl)-acetyl moiety on the benzimidazol-2-amino scaffold (**13**–**32**), which showed CK1δ inhibitor activity in the low micromolar range. Among the R substituents, 5-cyano was the most advantageous, leading to a compound endowed with nanomolar potency (**23**, IC_50_ = 98.6 nM). Molecular docking and dynamics studies were performed to point out the inhibitor–kinase interactions.

## 1. Introduction

Protein kinase CK1δ (CK1δ) is a serine/threonine kinase belonging to the casein kinase 1 family (CK1), and it is expressed in all eukaryotic organisms [1]. At least six human isoforms of CK1 (termed α, γ**1**–**3**, δ, and ε) have been cloned and characterized as displaying a highly conservative kinase domain [1]. CK1δ isoform is distributed both in the cytosol and in the nucleus and can be associated with membranes, receptors, transport vesicles, cytoskeleton components, and centrosomes, depending on the conditions of the cell [1,2,3]. CK1δ isoform modulates several physiological processes, such as circadian rhythm [4], DNA damage repair, cellular proliferation, and apoptosis [5]. Expression of CK1δ can vary depending on the tissue and cell types and according to physiological and pathological conditions. CK1δ overexpression and the consequent hyperphosphorylation of its substrates can lead to a dysregulation of transduction signaling and cell functions. CK1δ plays a role in oncogenesis due to the control it exerts on Wnt/β-catenin-, p53-, Hedgehog-, and Hippo-mediated pathways, whose dysfunction can influence the development of cancer cells [5,6,7,8]. A high expression of CK1δ was found to be a factor associated with a poor prognosis in patients affected by glioblastoma, lung cancer, and colorectal cancer [5,6,9].

Clear evidence points out that CK1 isoforms could affect the development of central nervous system (CNS) diseases. Familial advanced sleep-phase syndrome (FASPS), a disorder of the circadian rhythm [10], has been linked to CK1δ mutations at the level of the binding domain of Period 2 protein. In Alzheimer’s disease (AD) patients, a higher expression of CK1δ in the hippocampus was found compared to the control group, and high levels of CK1δ were evidenced in the neuritic plaques [11]. CK1δ phosphorylates tau protein in vitro, leading to the destruction of microtubules [12]. CK1δ also phosphorylates TDP-43 (transactive response DNA-binding protein, 43 kDa), a protein contributing to the onset of neurodegenerative processes. Aberrant TPD-43 phosphorylation seems to occur in the earliest phases of amyotrophic lateral sclerosis (ALS) and frontotemporal lobe degeneration (FTLD) [13,14]. Aggregates of phosphorylated TPD-43 were found in patients suffering from ALS, FTLD, and other neurodegenerative diseases, including Parkinson’s disease (PD) and AD [15,16]. CK1 showed the ability to phosphorylate α-synuclein in both in vitro and in vivo experiments [17,18]. α-Synuclein is recognized as the hallmark of PD, being implicated in the pathogenesis of the illness. Phosphorylated α-synuclein is the major component of Lewy bodies in the brain of sporadic PD patients and is also present in some cases of familial AD and dementia [19]. CK1 and α-synuclein aggregates were found to be colocalized in the post-mortem brains of patients suffering from PD and Lewy-body dementia [20]. Parkin is another protein whose mutation and dysfunction have been linked to inherited and sporadic PD. Parkin phosphorylation by CK1δ leads to reduced solubility of the protein, thus causing its aggregation and inactivation. Higher levels of phosphorylated parkin were found in the caudate nucleus of sporadic PD patients [21] compared to controls. Altogether, these results highlight the fact that targeting CK1δ with inhibitors can be a therapeutic strategy to fight against cancer and neurodegenerative diseases.

**Figure 1:**
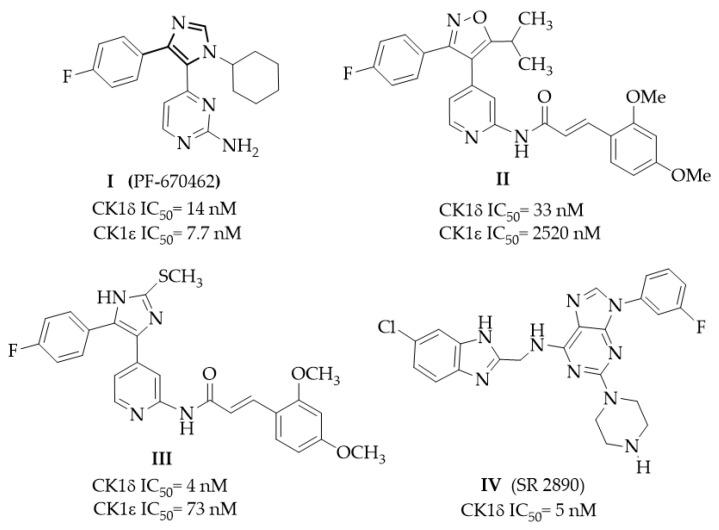
Some representative potent CK1δ inhibitors.

**Figure 2:**
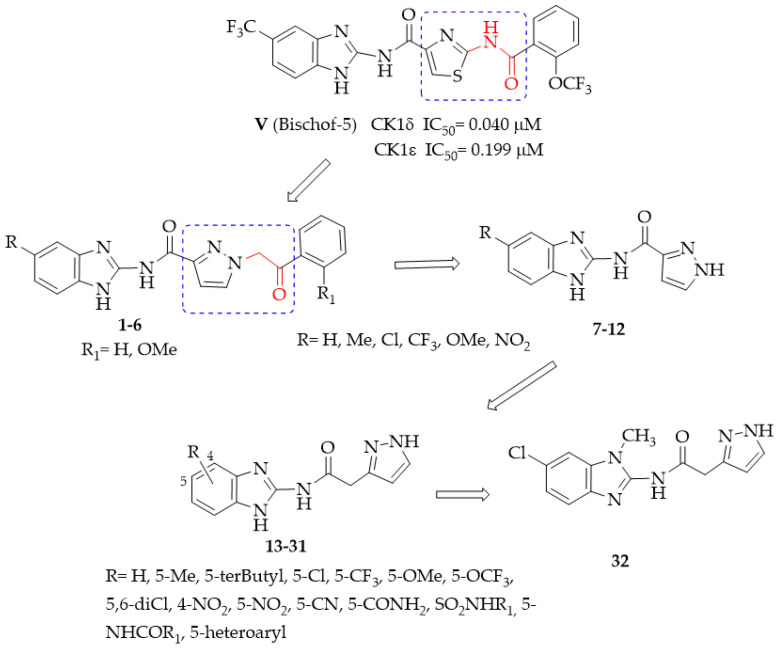
Bischof-5-based design of the herein-reported 2-amido-benzimidazole derivatives.

Since the early 2000s, much effort has been carried out to develop CK1δ inhibitors. Different classes of derivatives, either naturally occurring or suitably synthesized, showed the ability to bind the kinase ATP site [22]. From a structural point of view, an ATP-competitive CK1δ inhibitor typically consists of a central nitrogen-containing heterocycle decorated with various hydrophobic groups. In Figure 1, the structures of some potent CK1δ inhibitors are reported. Compound **I** (PF-670462) was one of the first potent CK1δ inhibitors identified (IC_50_ = 14 nM), but it was also active against CK1ε (IC_50_ = 7.7 nM) [23,24]. Derivative **I** was tested in a triple transgenic mouse model of AD, furnishing a proof of concept for CK1δ/ε inhibition to reduce AD-related cognitive deficits [25]. The oxazole derivative **II** [26] and its analog imidazole compound **III** showed high potency and improved selectivity versus the CK1ε isoform [27,28]. Compound **IV** (SR 2890) [29] is one of the most representative purine derivatives linked to a substituted benzimidazole-2-yl ring. Benzimidazole is the core nucleus of a deeply investigated series within which several potent inhibitors were identified, such as the series developed by Bischof [30]. Compound **V**, named Bischof-5 (Figure 2), emerged as a promising compound. It showed nanomolar CK1δ inhibitor activity and good selectivity versus CK1ε isoform and also demonstrated the ability to induce apoptosis on different tumor cell lines [30]. Bischof-5 analogs with reduced molecular size were developed to improve solubility and intracellular availability [31]. Poor solubility and limited cell membrane permeability are the concerns shared by several inhibitors showing nanomolar activity in enzyme assays while possessing poor efficacy in cellular and in vivo tests.

Thus, the discovery of new potent and selective CK1δ inhibitors can be of interest to overcome this issue. To identify new ATP-competitive CK1δ inhibitors, we designed the new benzimidazole derivatives **1**–**6**, taking Bischof-5 as the lead compound (Figure 2) and replacing its thiazole ring with a pyrazole nucleus, as well as replacing the NHCO linker to the terminal aryl group with a more flexible CH_2_CO residue.

The benzimidazoles **7**–**12**, lacking the appended lipophilic substituents, were designed as simplified analogs of **1**–**6**, and the third set of compounds (**13**–**32**) ensued from the elongation of the linker between the benzimidazole and pyrazole moieties. The latter modification was carried out to increase the structural flexibility of the compounds, which could ameliorate both solubility and complementarity with the target. Substituents with different electronic, lipophilic, and steric properties were inserted into the fused benzo ring, some chosen for their beneficial effects in the lead series. Molecular modeling investigations were performed to predict the kinase–ligand interactions and drive the design of the new compounds.

## 2. Results and Discussion

### 2.1. Chemistry

**Scheme 1:**
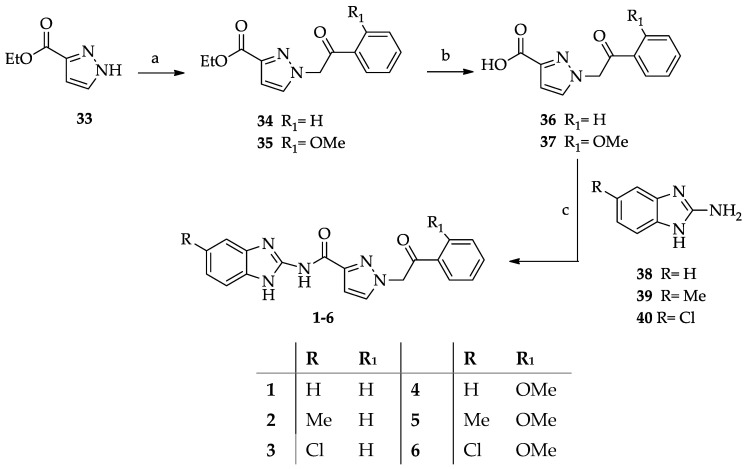
Reagents and conditions: (a) suitable ArCOCH_2_Br, acetonitrile, K_2_CO_3_; (b) 1M NaOH, EtOH; (c) EDCI HCl, HOBT, anhydrous DMF.

**Scheme 2:**
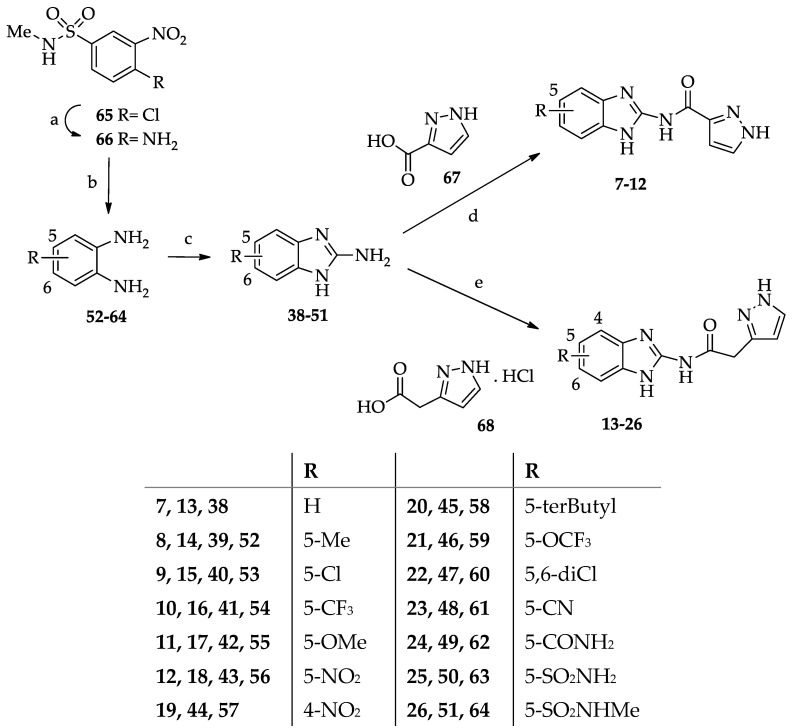
Reagents and conditions: (a) 33% aqueous NH_3_, 1,4-dioxane, 130 °C, sealed tube; (b) H_2_, Pd/C, Parr apparatus, EtOH, 35 psi; (c) (i) BrCN, CH_3_CN/H_2_O, T = 90 °C, (ii) saturated NaHCO_3_ to pH = 8; (d) from **38**–**43**, EDCI HCl, OH-BT, anhydrous DMF, rt, N_2_; (e) from **38**–**51**, Et_3_N, EDCI HCl, OH-BT, anhydrous DMF, rt, N_2_.

**Scheme 3:**
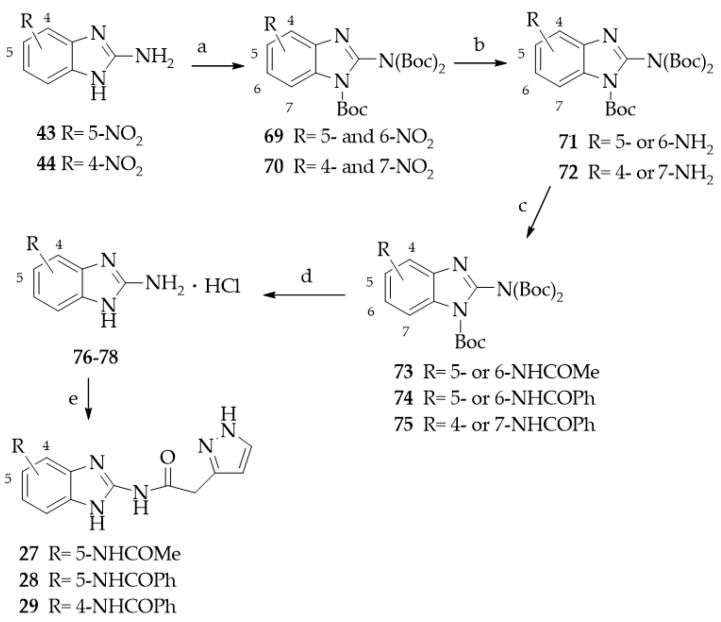
Reagents and conditions: (a) (t-BoC)_2_O, DMAP, anhydrous THF; (b) H_2_, Pd/C, MeOH, Parr apparatus, 35 psi; (c) RCOCl, Et_3_N, anhydrous CH_2_Cl_2_, rt; (d) (i) CF_3_COOH, CH_2_Cl_2_, rt, (ii) HClg saturated MeOH; (e) 2-(1H-pyrazol-3-yl)acetic acid hydrochloride **68**, EDCI HCl, HOBT, Et_3_N, DMF, rt.

The synthesis of the target compounds is shown in Scheme 1, Scheme 2 and Scheme 3. Derivatives **1**–**6** (Scheme 1) were obtained starting from the regioselective alkylation of ethyl pyrazole-3-carboxylate **33** with 1-phenyl- and 2-methoxyphenyl-2-bromoethanones, which gave compounds **34** [32] and **35**, respectively. The reaction was carried out in the conditions described to obtain **34**, i.e., by using acetonitrile as the solvent and in the presence of K_2_CO_3_ at r.t., which afforded the ethyl 1-alkyl-3-carboxylate derivatives **34** and **35** as the predominant regioisomers. The 1-substituted-3-carboxylate structure of **35** was confirmed using NOESY experiments, which showed a spatial closeness of the pyrazole hydrogen atom to the methylene protons.

Derivatives **34** and **35** were hydrolyzed to give the corresponding carboxylic acids **36** and **37**, which were reacted with the benzimidazol-2-amine derivatives **38**–**40** (prepared as described below) in anhydrous DMF and in the presence of 1-(3-(dimethylamino)-propyl))-3-ethylcarbodiimide (EDCI) hydrochloride and 1-hydroxybenzotriazole (HOBT) to give the desired compounds **1**–**6**.

Derivatives **7**–**26** were synthesized as depicted in Scheme 2. Compounds **7**–**12** were obtained by coupling the benzimidazole-2-amines **38**–**43** with pyrazole-3-carboxylic acid **67** in the experimental conditions employed to obtain derivatives **1**–**6**. Derivatives **13**–**26** were prepared by reacting 2-(3-pyrazolyl) acetic acid hydrochloride **68** [33] with benzimidazol-2-amines **38**–**51** in anhydrous DMF and the presence of triethylamine, EDCI hydrochloride, and 1-hydroxybenzotriazole. The benzimidazole-2-amine **38** was commercially available, and **39**–**43** were suitably synthesized by cyclizing the corresponding 1,2-phenylenediamines **52**–**56** with cyanogen bromide [34]. Similarly, **44** [35], **47** [34], and **48** [36] and the newly synthesized **45**, **46**, and **49**–**51** were prepared by cyclizing the suitable 1,2-phenylenediamines **57**–**64** with cyanogen bromide. Diamines **52**–**64** were commercially available, except for **59** [37], **62** [38], and **63** [39], which were obtained as previously reported, and **64**, which was synthesized starting from 4-chloro-N-methyl-3-nitrobenzensulphonamide **65** [40]. Treatment of **65** with 33% ammonia aqueous solution gave the corresponding 4-amino derivative **66**, which was catalytically reduced to the diamine **64**.

Derivatives **27**–**29** were synthesized as depicted in Scheme 3. The 5-nitro- and 4-nitro-substituted benzimidazole-2-amines **43** [34] and **44** [35], respectively, were reacted with di-tert-butyl-dicarbonate to give their respective triply N-Boc protected derivatives, **69** [41] and **70**, as a mixture of regioisomers as a result of benzimidazole tautomerism. Derivative **69** was a mixture of 5-nitro- and 6-nitro-substituted isomers, and **70** was a mixture of 4-nitro and 7-nitro isomers. The regioisomers were not separated but used as such in the next step.

Catalytic hydrogenation of compounds **69** and **70** yielded the corresponding amino derivatives **71** [41] and **72**, each constituted by only one isomer (see Section 3 for details). Compound **71** was reacted with acetyl chloride or benzoyl chloride to give derivatives **73** and **74**, respectively [42].

**Scheme 4:**
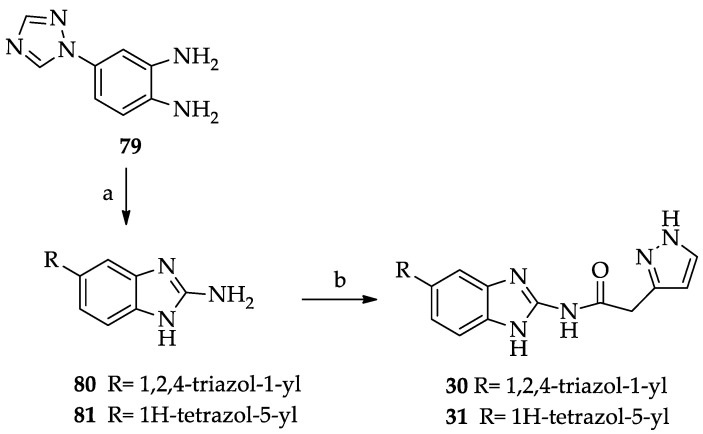
Reagents and conditions: (a) (i) BrCN, MeOH/H_2_O, (ii) NaHCO_3_-saturated solution; (b) 2-(1H-pyrazol-3-yl)acetic acid hydrochloride **68**, Et_3_N, EDCI HCl, OH-BT, anhydrous DMF, rt, N_2_.

Compound **72** was transformed into derivative **75** by reaction with benzoyl chloride. The N-Boc derivatives **73**–**75** were deprotected by treatment with trifluoroacetic acid and then transformed in the hydrochlorides **76**, **77** [42], and **78**. Finally, these derivatives, after treatment with triethylamine, were coupled with pyrazol-3-yl acetic acid hydrochloride **68** in the experimental conditions reported above for **13**–**26**, to give compounds **27**–**29**. Derivatives **30** and **31**, bearing a five-membered heterocyclic substituent at position 5, were prepared as shown in Scheme 4. The synthesis of the 5-(1,2,4-triazol-1-yl) derivative **30** started from the preparation of the suitable 1,2-phenylenediamine **79** [43], which was transformed into the corresponding benzimidazol-2-amino derivative **80** by cyclization with cyanogen bromide. The 5-(tetrazol-5-yl) derivative **81** was prepared as previously described [44]. Derivatives **80** and **81** were reacted with pyrazol-3-yl acetic acid hydrochloride **68** in the experimental conditions employed for **13**–**26**, to give, respectively, compounds **30** and **31**.

**Scheme 5:**
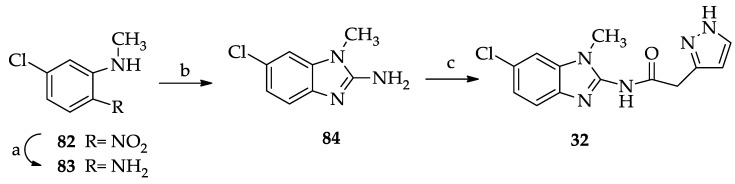
Reagent and conditions: (a) H_2_, Ni/Raney, Parr apparatus, EtOH, 15 psi; (b) (i) BrCN, MeOH/H_2_O, T = 60 °C, (ii) 1M NaOH solution; (c) 2-(3-pyrazolyl)acetic acid hydrochloride **68**, Et_3_N, EDCI HCl, OH-BT, anhydrous DMF, rt, N_2_.

The N-methylbenzimidazole derivative **32** was synthesized as reported in Scheme 5, starting from 5-chloro-2-nitro-N-methylaniline **82** [45], which was catalytically reduced to give the corresponding amino derivative **83**. Cyclization of the latter with cyanogen bromide afforded the benzimidazol-2-amino derivative **84**, which was coupled with pyrazol-3-yl acetic acid **68** to yield the desired final compound **32**.

The structures of the benzimidazole-2-amine derivatives, both intermediates and final compounds, are drawn as single tautomers, i.e., not considering the prototropic exchange of the benzimidazole-NH and pyrazole-NH. Thus, the structures drawn in figures and schemes are those with the substituents numbered as low as possible on both rings.

### 2.2. Structure–Activity Relationship (SAR) Studies

The newly synthesized derivatives **1**–**32** were tested on truncated CK1δ using the luminescent kinase assay Kinase Glo^®^ KIT (Promega Italy S.r.l., Milan, Italy), and the results are reported in Table 1 and Table 2. Derivatives were tested at a fixed dose of 40 μM; then, those showing a residual kinase activity percentage lower than 50% were tested at 10 μM. For compounds exhibiting a residual enzyme activity percentage lower than 50% at the latter concentration, the IC_50_ values were determined.

This study has led to the identification of some compounds (**2**, **3**, **6**, **13**–**24**, **26**, **30**, and **31**) showing CK1δ inhibitor activity in the micromolar and submicromolar range (IC_50_ < 15 μM), the most active being derivative **23** (R = CN), endowed with nanomolar activity (IC_50_ = 98.6 nM).

The first set of derivatives (**1**–**6**) was designed taking Bischof-5 as the lead compound and replacing its thiazole ring with a pyrazole. Moreover, to confer more flexibility to the molecules, the NHCO function linking the aryl pendant was replaced by a CH_2_CO linker. Simple substituents (R) were introduced at the 5-position of the fused benzo ring. The unsubstituted derivative **1** (R = H) was scarcely active (IC_50_ > 40 μM), and the presence of a methyl or a chlorine atom enhanced activity, as it appears from the IC_50_ values of **2** (R = Me) and **3** (R = Cl) (IC_50_ = 14.6 and 1.80 μM, respectively). The chlorine atom is particularly effective in reinforcing the hydrophobic interaction of the molecule with the kinase, and this was confirmed in the set of compounds **4**–**6**, where the introduction of an ortho-OMe group on the benzoyl pendant was detrimental to the inhibitory activity. Derivatives **4**–**6** were less active than **1**–**3**. However, also in this set, the 5-chloro-substituted derivative **6** was the best (IC_50_ = 13.2 μM), whereas compounds **4** (H) and **5** (R = Me) were scarcely active (IC_50_ > 40 μM).

These results prompted us to substantially modify the structure of the compound, and by applying a molecular simplification approach, compounds **7**–**12** were designed. In these derivatives, the lipophilic substituent on the pyrazole was removed to evaluate whether structurally simpler molecules than **1**–**6** retained the ability to bind the kinase. On the fused benzo ring, apart from Cl and Me, other groups with different lipophilic and electronic properties were probed (CF_3_, OMe, and NO_2_). As shown in Table 1, no derivative of this set, tested at 40 μM, was able to inhibit CK1δ with a significant potency (IC_50_ values > 40 μM).

Significantly better results were obtained with the third set of derivatives (**13**–**32**), where the pyrazole ring was distanced through a methylene spacer from the carboxamide bond (Table 2).

This structural modification was suggested by preliminary docking studies (see Section 2.3), which evidenced a better accommodation of these molecules to the kinase ATP site.

The increased flexibility of **13**–**32** was also envisaged to improve their water solubility, compared to that of their more rigid analogs **7**–**12**. As shown in Table 2, most of the compounds **13**–**32** inhibited CK1δ activity, with IC_50_ values in the low μM range, and one (**23**, R = CN) displayed a nanomolar potency (IC_50_ = 98.6 nM).

The first synthesized derivatives of this set were **13**–**18**, bearing the same R substituents of their inferior homologs **7**–**12**. Interestingly, the new compounds were significantly more active (IC_50_ = 0.12–6.27 μM) than **7**–**12** (IC_50_ > 40 μM), thus pointing out the key role of the methylene spacer in improving the complementarity between the inhibitor and the kinase. Regarding the effect of the R substituent on **13**–**18**, the groups affording the highest activity are lipophilic, the best being the 5-NO_2_ (**18**, IC_50_ = 0.12 μM) and the 5-Cl (**15**, IC_50_ = 0.485 μM).

Instead, the presence of the hydrophilic 5-OMe group reduced the CK1δ inhibitor activity (**17**, IC_50_ = 6.27 μM). Compound **16**, bearing the 5-CF_3_ substituent (IC_50_ = 1.74 μM), present in the lead Bishof-5, was as potent as the 5-Me derivative **14** (IC_50_ = 1.64 μM). Due to these encouraging results, further modifications were carried out on the benzimidazole-2-acetamide series. Movement of the NO_2_ group from the 5- to the 4-position gave derivative **19**, which was 10-fold less potent than the regioisomer **18**, although maintaining CK1δ inhibitor activity (IC_50_ = 1.22 μM). Among the new substituents introduced at the 5-position, the most effective was the CN, which led to the most active compound of the series (**23**, IC_50_ = 98.6 nM).

The 5-terbutyl-substituted derivative **20** proved to be able to inhibit CK1δ (IC_50_ = 1.00 μM) with higher potency, compared to the unsubstituted compound **13** (IC_50_ = 4.21 μM). This result would confirm that hydrophobic groups reinforce the interaction with the kinase and indicate that the pocket where the benzo ring is accommodated is roomy enough to allow for the insertion of the terbutyl residue. Coherent to this is the inhibitory effect of the 5,6-dichloro derivative **22** (IC_50_ = 0.98 μM). In contrast, the 5-OCF_3_ lipophilic group gave a compound (**21**, IC_50_ = 5.99 μM) whose CK1δ inhibitory activity was comparable to that of the 5-OMe derivative **17**.

Compounds **24**–**29** feature amide substituents of different sizes and properties on the fused benzo ring. The 5-CONH_2_ substituent proved to be the best among those evaluated, as it afforded a quite good CK1δ inhibitor (**24**, IC_50_ = 2.53 μM). The acidic 5-SO_2_NH_2_ group was chosen for its potential ability to be engaged in an ionic interaction with Lys 38 residue of the selectivity pocket of the kinase. Instead, this group was disadvantageous for CK1δ inhibitor activity. In fact, derivative **25** showed an IC_50_ value >40 μM. Methylation of the sulphonamide group, to give derivative **26** (R = SO_2_NHMe), made a certain activity appear (IC_50_ = 10.7 μM). A detrimental effect was achieved by the 5-NHCOMe (**27**), 5-NHCOPh (**28**), and 4-NHCOPh (**29**) residues, which dropped the ability to inhibit the kinase (IC_50_ > 40 μM). At the 5-position, derivatives **30** and **31** bear, respectively, the 1,2,4-triazol-1-yl and the tetrazol-5-yl residues, both able to form H-bonding interaction, and the latter possessing acidic properties. Both compounds, **30** and **31**, were active as CK1δ inhibitors (**30**, IC_50_ = 2.59 μM; **31**, IC_50_ = 1.54 μM). The rationale for the synthesis of the N-methyl derivative **32** was to evaluate whether the blockade of the tautomerism could reinforce the interaction with the kinase. Instead, the N-methyl-6-chloro derivative **32** showed a significantly reduced activity against CK1δ (IC_50_ = 20.1 μM) compared to its NH analog **15**.

### 2.3. Physicochemical and Pharmacokinetic Parameters

Some physicochemical and pharmacokinetic parameters, such as predicted logP, logS, logBB, blockage of HERG K+ channels, Caco-2 cell permeability, binding to human serum albumin, and oral absorption, were calculated for the most active compounds, **15**, **18**, **22**, **23**, and the reference **PF-670462** (see Section 3.3.10). The results are reported in the Supplementary Materials (Table S1). On the whole, the calculated parameters suggested good pharmacokinetics properties of the new inhibitors, such as good to high oral absorption and good ability to cross the blood–brain barrier.

### 2.4. Molecular Modeling Studies

In the molecular modeling studies conducted on the synthesized compounds, a computational structure-based approach was carried out, starting from the available crystallographic complexes in the Protein Data Bank [46]. A CK1δ 3D structure was selected on the basis of the closest scaffold similarity between the co-crystallized inhibitor and the series under investigation. The selection was made in favor of the CK1δ structure coded as 5OKT, in complex with a 2-aminobenzothiazole-like inhibitor. The docking reliability was validated by redocking the PDB crystallographic ligand (IWP-2) to the protein ATP-binding site. The docking algorithm was able to recreate the crystallo-graphic pose (Figure S1) with a low heavy-atom RMSD value of 2.1 Å (the same range of the crystallographic resolution), reaching the value of 0.86 Å when focusing on the 2-aminobenzothiazole scaffold.

The synthesized compounds were docked at the CK1δ binding site. Two docking runs were made, either neglecting or considering two key crystallographic water molecules in the ATP-binding site. In fact, it is well established that water molecules could play an active role in mediating hydrogen-bonding interactions with the ATP-competitive inhibitors within the binding site [47]. The first run of docking simulations has been performed excluding all water molecules during the ligand-posing step. In parallel, a second docking run has been carried out considering two water molecules (labeled w295 and w296 in the following analysis) in direct contact with the amino acid residues Tyr56, Glu52, and Lys38. As already anticipated, these two water molecules are highly conserved inside the ATP-binding cleft, and they directly contact the inhibitor in several crystallographic structures [47].

The selection of the representative binding poses has been carried out through a combined approach based on the analysis of electrostatic and van der Waals interactions between the ligand and its recognition site, and subsequently by filtering the poses through a pharmacophore model built on the strongest interactions of the co-crystallized inhibitor with CK1δ in structure 5OKT (see Section 3.3 for details). In particular, the pharmacophore filtering step retained only the docking poses capable of preserving a bi-dentate hydrogen bond with Leu85 residue, as well as the positions of the aromatic groups of the benzothiazole ring. An overview of the selected poses is provided in the video included in the Supplementary Materials (Video S1). In the video’s background, heat maps of electrostatic and hydrophobic energies are depicted for each ligand in the series concerning the residues primarily involved in the binding site.

The general binding mode of the compounds in this series highlights the presence of the aromatic benzimidazole scaffold, which is capable of forming a bidentate hydrogen bond with Leu85 and of interacting with the numerous hydrophobic residues of the binding pocket, such as Ile15, Ile23, Ala36, Leu135, and Ile147. Regarding substitutions at positions 4, 5, and 6 of the benzimidazole ring, the presence of substituents with a strong electrostatic component is emphasized, enabling the mediation of hydrogen-bonding interactions with residues like Tyr56 and Lys38. The mentioned residues are common in other previously published computational works [48,49,50]. As anticipated, in addition to these residues, at least two water molecules can play a crucial role in mediating additional hydrogen bonds.

**Figure 3:**
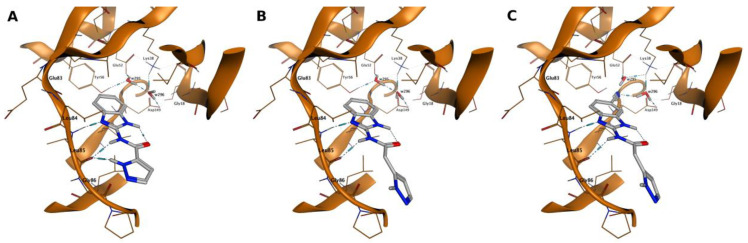
The proposed binding mode of compounds **7** (panel (**A**)), **13** (panel (**B**)), and **23** (panel (**C**)); protein residues mainly involved in binding are shown as sticks.

**Figure 4:**
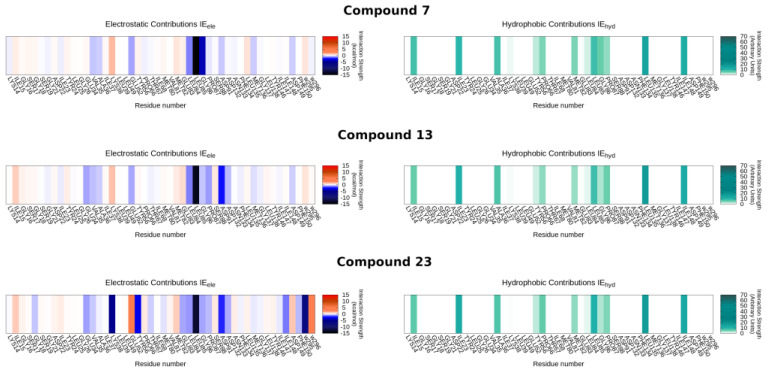
Interaction Energy Fingerprints (IEFs) comparison between compounds **7**, **13**, and **23**; on the left side, the electrostatic contribution comparison is shown, and the hydrophobic one is shown on the right.

To inspect the plausible structure–activity relationship of the series of inhibitors, we have focalized the attention to compounds **7**, **13**, and **23**, combining the binding mode analysis (Figure 3) with the comparison of their Interaction Energy Fingerprint (IEF) profiles (Figure 4). Briefly, compound **7** is a putative representative of compounds **1**–**12**, and compounds **13** and **23** can help to decipher the inhibitory behaviors of compounds **13**–**32**.

The binding pocket of CK1δ is mainly composed of hydrophobic residues, with few residues capable of mediating hydrogen bonds or ionic interactions, such as Tyr56, Lys38, and Glu52. As anticipated, in addition to these residues, there are at least two water molecules that can play a crucial role in mediating additional hydrogen bonds.

The high concentration of internal hydrophobic residues helps explain the enhanced activity values observed for compounds with lipophilic substitutions at position 5, as mentioned in the previous section. This holds true for both the first dataset of compounds (**1**–**12**) and the second cluster (**13**–**32**).

As anticipated, compound **7** (IC_50_ > 40 µM) is representative of compounds **1**–**12** belonging to the pyrazole-amide class of analogs, characterized by a planar conformation (Figure 3A). The planarity of these structures leads to notable steric clashes inside the binding cavity, reducing the pose adaptability within the pocket.

The introduction of a methylene spacer between the pyrazole moiety and the amide group, as in compounds **13**–**32**, improved the corresponding inhibitory activities of the series; and this is clearly exemplified by the activity of compound **13** (IC_50_ = 4.21 µM), which is analogous to compound **7**, with the unique addition of a methylene spacer at the side chain. This elongation increases the flexibility of the side chain and, consequently, might enhance the adaptability of the inhibitor inside the binding cleft.

In addition to this structural consideration, the role played by the substituent in position 5 of the benzimidazole system appears evident from the structure–activity analysis. Compound **23**, chosen as a reference structure, exhibits the highest inhibitory activity in the entire series (IC_50_ = 0.0986 µM) and shares chemical similarities with compounds **18, 19**, and **24**. All these derivatives have a substituent that is able to establish hydrogen bonding with the water molecules inside the binding site, improving the inhibitory activities of the compounds.

However, the correct balance between the substituent’s ability to interact with the water molecules and its volume (steric hindrance properties) contributes to determining the inhibitory capacity of these analogs, as demonstrated by the activities measured for compounds **25**–**29**. Interestingly, compounds **30** and **31**, with triazole and tetrazole, were designed to establish a direct interaction with Lys38. This strategy led to measurable activity in the low micromolar range but not to the expected extent. The displacement of water molecules to achieve a direct interaction, especially water 295, seems to be disfavored compared to mediating a hydrogen bond interaction, emphasizing the importance of water molecules within the binding site.

To better interpret the binding process of this class of inhibitors, molecular dynamics simulations were performed. In particular, SuMD (Supervised Molecular Dynamics) and TTMD (Thermal Titration Molecular Dynamics) were used for studying the binding and unbinding events.

SuMD is an enhanced sampling Molecular Dynamics-based method designed for studying ligand–target association processes at the nanosecond timescale. It integrates classical molecular dynamic simulations with a tabu-like algorithm to increase the probability of observing a binding event [51]. On the other hand, TTMD is a computational approach that conducts classical molecular dynamics simulations at increasing temperatures. It uses temperature to boost the system’s kinetic energy and promote events that would otherwise require much longer timescales [52]. These techniques provide valuable insights into the binding and unbinding kinetics of compounds with the studied scaffold. Compounds **13** and **23** have been selected as representative examples of this class of inhibitors, in particular to interpret at a molecular level the role of the nitrile group in position 5 and the cooperative role of water molecules in stabilizing the positioning of the ligand within the binding pocket.

**Figure 5:**
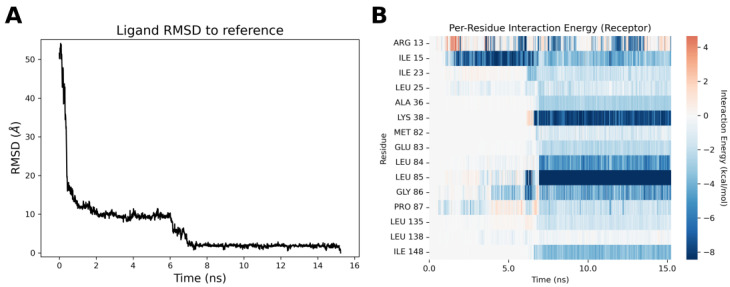
On the left (**A**), the ligand’s RMSD values during the simulation can be seen compared to its reference (docking pose). On the right (**B**), the image of the “Per Residue” analysis was performed on the 15 residues most frequently contacted by the ligand during the simulation.

These analyses typically include measurements of RMSD (Root Mean Square Deviation) compared with the reference structure, which, in this case, is the docking pose obtained earlier. Additionally, per-residue interaction analyses provide insights into the interactions between the ligand and various amino acid residues throughout the simulation. These analyses, in Figure 5 and Video S2, help to determine how the ligand interacts with the protein structure during the binding process.

**Figure 6:**
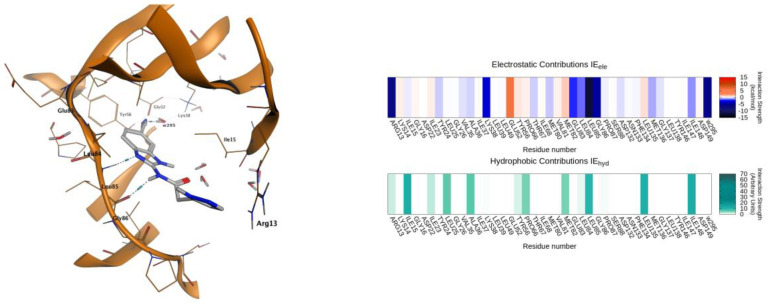
The binding mode of the last frame of the molecular dynamics simulation is on the left, and its heat maps of IE_ele_ and IE_hyd_ are on the right.

The inspection of the ligand-binding trajectory clearly shows a preliminary binding event in proximity to the external portion of the P-loop of the protein, in which a stabilizing interaction with Ile15 takes place. When the ligand enters the binding site, both the ligand pose and its interaction profile are perfectly in agreement with those observed in the docking analyses. In particular, a strong stabilizing interaction with Leu85 of the hinge region is observed, together with a hydrogen bond interaction between a water molecule and the nitrogen nitrile group. It is worth underlining that this portion of the binding cleft is occupied by a water molecule during the whole simulation time, and as in Figure 6, it could represent the water w295 already described in the docking simulations.

**Figure 7:**
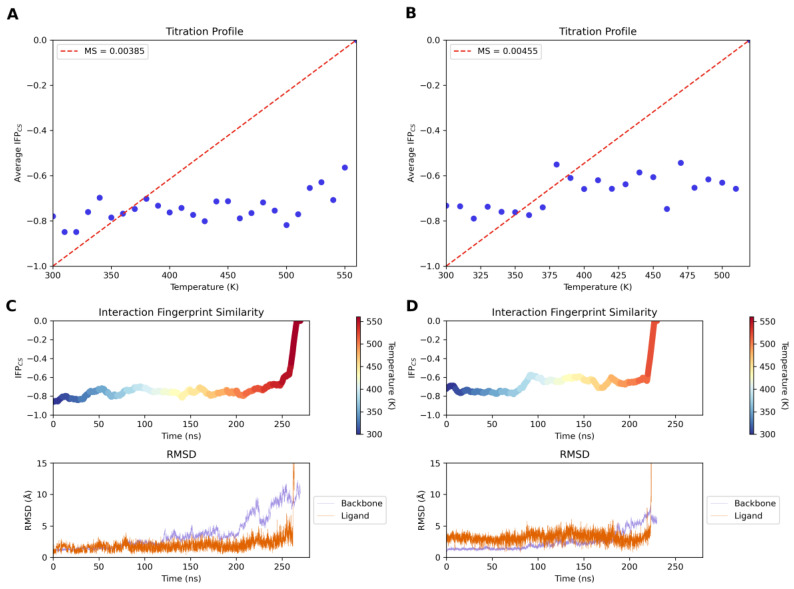
(**A**) Titration profile of compound **23**. (**B**) Titration profile of compound **13**. (**C**) Interaction Fingerprint Similarity and RMSD values of compound **23**. (**D**) Interaction Fingerprint Similarity and RMSD values of compound **13**.

In parallel with the exploration of the binding trajectories of compounds **13** (IC_50_ = 4.21 ± 1.92 µM) and **23** (IC_50_ = 0.0986 ± 0.0394 µM), a molecular dynamics study of the unbinding event was also conducted using TTMD (Thermal Titration Molecular Dynamics) to evaluate the stability of a ligand–protein complex [53]. For both compounds, five simulations were conducted, starting at 300 K and gradually increasing the temperature until the binding mode was lost, resulting in the ligand exiting the binding pocket. The starting point for the dynamic simulations was the docking poses obtained previously. The analyses of the trajectories are shown in Figure 7 and Video S3. The “titration profile” plot reports the average IFP_CS_ value for each TTMD step as a function of the step temperature.

This metric makes it easier to qualitatively compare the stabilities of various protein–ligand complexes. A straight line joining the initial and final states of the simulation is also drawn in the graph, and its slope (termed MS coefficient), quantifying the stability of the protein–ligand complex, is reported in the legend. The second graph depicts the “Interaction Fingerprint Similarity”, which provides information on how the interaction pattern changes over time as compared to the initial reference frame (a colorimetric scale reports temperature). The third and final plots report the time-dependent evolution of the ligand and protein backbone RMSD value [53].

For compound **23**, it is evident that in the reported simulation, similarly to the other replicates, the ligand pose remains stable throughout the temperature gradient explored. The interaction similarity values compared to the initial reference, starting at a value below −0.8, also remain constant throughout the temperature gradient explored until the ligand exits the binding site. This steady trend is also reflected in the ligand’s RMSD values, which remain stable throughout the simulation. Interestingly, compound **23** loses its binding state only at temperatures above 560 K, when a significant destructuring of the tertiary structure of the protein begins to be noticed. On the contrary, compound **13** shows a loss of the interaction profile at a lower temperature than compound **23**, indicating that this ligand may have a shorter residence time due to the lack of the nitrile substituent.

These findings suggest that the presence of specific hydrogen bond-accepting substituents at position 5, like the cyano group, plays a crucial role in enhancing the binding affinity and inhibitory activity of the ligand by mediating interactions with internal water molecules within the binding site. This highlights the significance of considering both the protein–ligand interactions and solvent-mediated interactions for a comprehensive understanding of ligand binding and activity.

## 3. Materials and Methods

### 3.1. Chemistry

#### General Methods

All the commercially available reagents and solvents were used as purchased from Merck without further purification. Analytical silica gel plates 60 F_254_ (Merck Life Science S.r.l., Milan, Italy) and silica gel 60 (Merck, 70-230 mesh) were used for analytical TLC and column chromatography, respectively. All melting points were determined on a Gallenkamp melting point apparatus and were uncorrected. Compounds were named following IUPAC rules as applied by ChemDrawUtra 9.0. Elemental analyses were performed on unknown compounds with a Flash E1112 Thermofinnigan elemental analyzer for C, H, and N, and the results were within ± 0.4% of the theoretical values. All final compounds revealed purity not less than 95%. Catalytic reductions were performed in a Parr hydrogenation apparatus (Parr Instrument Company, Moline, IL, USA). NMR spectra were recorded on a Bruker Avance 400 spectrometer (400 MHz, Bruker Italia S.r.l., Milan, Italy). The chemical shifts are reported in δ (ppm) and are relative to the central peak of the solvent, which was CDCl_3_ or DMSOd6. The following abbreviations are used: s = singlet, d = doublet, t = triplet, q = quartet, m = multiplet, br = broad, and ar = aromatic protons. Scanned ^1^H-NMR spectra of compounds **1**–**32** are reported in the Supplementary Materials.

General procedure for the synthesis of N-(1H-benzimidazol-2yl)-1-(2-arylethyl-2-oxo)-1H-pyrazole-3-carboxamides **1**–**6**.

A mixture of the suitable N1-substituted pyrazole-3-carboxylic acid **36** or **37** (4.2 mmol), 1-hydroxybenzotriazole (2.5 mmol), and EDCI HCl (4.2 mmol) in anhydrous DMF (about 1.5 mL) was stirred at r.t. for 15 min under nitrogen atmosphere, then the commercially available benzimidazol-2-amine **38** or the suitably synthesized 2-benzimidazol-2-amino derivative **39** or **40** [34] (2.5 mmol) was added. The mixture was stirred at r.t. for about 16 h, then cooled at T = 0 °C and diluted with H_2_O (20 mL). The solid was collected by filtration, washed with H_2_O (about 5–8 mL), dried, and purified by silica gel column chromatography.

N-(1H-Benzimidazol-2yl)-1-(2-oxo-2-phenylethyl)-1H-pyrazole-3-carboxamide (**1**). Column eluent CHCl_3_ 9/MeOH 1. Yield 10%_;_ mp = 220–221 °C (cyclohexane/EtOAc).^1^H NMR (DMSOd_6_) 12.22 (br s, 1H, NH), 11.20 (br s, 1H, NH) 8.08 (d, 2H, ar, J = 7.7 Hz), 7.92 (s, 1H, pyrazole proton), 7.75 (t, 1H, ar, J = 7.3 Hz), 7.62 (t, 2H, ar, J = 7.6 Hz), 7.47 (br s, 2H, ar), 7.12–7.10 (m, 3H, 1 pyrazole proton + 2 ar), 6.03 (s, 2H, CH_2_). Elemental analysis calcd for C_19_H_15_N_5_O_2_: C, 66.08; H, 4.38; N, 20.28; found C, 66.36; H, 4.50; N, 20.33.

N-(5(6)-Methyl-1H-benzimidazol-2yl)-1-(2-oxo-2-phenylethyl)-1H-pyrazole-3-carboxamide (**2**). Column eluent CHCl_3_ 9/MeOH 1. Yield 15%; mp 199–200 °C. ^1^H NMR (DMSOd_6_) 12.12 (br s, 1H, NH), 11.47 (br s, 1H, NH), 8.08 (d, 2H, ar, J = 7.1 Hz), 7.91 (br s, 1H, pyrazole proton), 7.75 (t, 1H, ar, J = 7.3 Hz), 7.63 (t, 2H, ar, J = 7.3 Hz), 7.38–7.23 (m, 2H, benzimidazole protons), 7.08 (br s, 1H, pyrazole proton), 6.94 (d, 1H, ar, J = 7.4 Hz), 6.02 (s, 2H, CH_2_), 2.38 (s, 3H, CH_3_). Elemental analysis calcd for C_20_H_17_N_5_O_2_: C, 66.84; H, 4.77; N, 19.49; found C, 67.04; H, 4.82; N, 19.55.

N-(5(6)-Chloro-1H-benzimidazol-2yl)-1-(2-oxo-2-phenylethyl)-1H-pyrazole-3-carboxamide (**3**). Column eluent CHCl_3_ 8.5/CH_3_CN 1.5. Yield 20%; mp = 200–203 °C. ^1^H NMR (DMSOd_6_) 12.39 (br s, 1H, NH), 11.49 (br s, 1H, NH), 8.08 (d, 2H, ar, J = 7.6 Hz), 7.94 (d, 1H, pyrazole proton, J = 2.1 Hz), 7.75 (t, 1H, ar, J = 7.4 Hz), 7.62 (t, 2H, ar, J = 7.6 Hz), 7.53–7.45 (m, 2H, ar), 7.13 (m, 2H, 1 ar + 1 pyrazole proton), 6.04 (s, 2H, CH_2_). Elemental analysis calcd for C_19_H_14_ClN_5_O_2_: C, 60.09; H, 3.72; N, 18.44; found C, 60.16; H, 3.81; N, 18.51.

N-(1H-Benzimidazol-2yl)-1-(2-(2-methoxyphenyl)-2-oxoethyl)-1H-pyrazole-3-carboxamide (**4**). Column eluent CHCl_3_ 9.5/MeOH 0.5. Yield 10%; mp = 168–170 °C. ^1^H NMR (DMSOd_6_) 12.20 (br s, 1H, NH), 7.92 (d, 1H, pyrazole proton, J = 1.9 Hz), 7.78 (d, 1H, ar, J = 7.6 Hz), 7.67 (t, 1H, ar, J = 7.2 Hz), 7.47 (br s, 2H, ar), 7.29 (d, 1H, ar, J = 8.4 Hz), 7.16–7.04 (m, 4H, 3ar + 1 pyrazole proton), 5.77 (s, 2H, CH_2_), 4.01 (s, 3H, CH_3_). Elemental analysis calcd for C_20_H_17_N_5_O_3_: C, 63.99; H, 4.56; N, 18.66; found C, 64.10; H, 4.67; N, 18.71.

N-(5(6)-Methyl-1H-benzimidazol-2yl)-1-(2-(2-methoxyphenyl)-2-oxoethyl)-1H-pyrazole-3-carboxamide (**5**). Column eluent CHCl_3_ 6.5/cyclohexane 2/MeOH 1.5. Yield 10%; mp = 201–203 °C. ^1^H NMR (DMSOd_6_) 12.07 (br s, 1H, NH), 11.27 (br s, 1H, NH), 7.91 (br s, 1H, pyrazole proton), 7.77 (d, 1H, ar, J = 7.7 Hz), 7.67 (t, 1H, ar J = 7.8 Hz), 7.39–7.20 (m, 3H, ar), 7.11 (t, 1H, ar, J = 7.3 Hz), 7.04 (s, 1H, pyrazole proton), 6.93 (d, 1H, ar, J = 7.9 Hz), 5.75 (s, 2H, CH_2_), 4.00 (s, 3H, OCH_3_), 2.38 (s, 3H, CH_3_). Elemental analysis calcd for C_21_H_19_N_5_O_3_: C, 64.77; H, 4.92; N, 17.98; found C, 64.59; H, 4.99; N, 18.02.

N-(5(6)-Chloro-1H-benzimidazol-2yl)-1-(2-(2-methoxyphenyl)-2-oxoethyl)-1H-pyrazole-3-carboxamide (**6**). Column eluent CHCl_3_ 9/ CH_3_CN 1. Yield 61%; mp = 219–221 °C. ^1^H NMR (DMSOd_6_) 12.37 (br s, 1H, NH), 11.41 (br s, 1H, NH), 7.94 (d, 1H, pyrazole proton, J = 2.3 Hz), 7.78 (dd, 1H, ar, J = 7.7, 1.7 Hz), 7.71–7.64 (m, 1H, ar), 7.51–7.47 (m, 2H, ar), 7.29 (d, 1H, ar, J = 8.4 Hz), 7.15–7.07 (m, 3H, 2ar + 1 pyrazole proton), 5.77 (s, 2H, CH_2_), 4.01 (s, 3H, CH_3_). Elemental analysis calcd for C_20_H_16_ClN_5_O_3_: C, 58.61; H, 3.94; N, 17.09; found C, 58.66; H, 4.02; N, 17.13.

General procedure for the synthesis of N-(benzimidazol-2-yl)pyrazole-3(5)-carboxamide derivatives **7**–**12**.

A mixture of the commercial benzimidazol-2-amine **38** (2 mmol) or the suitably synthesized benzimidazol-2-amino derivatives **39**–**43** [34] (2 mmol), pyrazole-3-carboxylic acid **67** (3 mmol), 1-hydroxybenzotriazole (2 mmol), and EDCI hydrochloride (3.3 mmol) in anhydrous DMF (about 1 mL) was stirred at r.t. under nitrogen atmosphere for 16 h. After cooling at 0 °C, the mixture was diluted with H_2_O (circa 20 mL), and the suspended solid was collected by filtration, washed with abundant H_2_O (5–8 mL), dried, and recrystallized. In the case of compound **10** (R = CF_3_), the crude solid was treated with boiling acetone (70 mL) for 10 min, then the mixture was cooled at r.t., and the suspended solid was collected by filtration and recrystallized.

N-(1H-Benzimidazol-2-yl)-1H-pyrazole-3(5)-carboxamide (**7**). Yield 60%; mp > 300 °C (EtOH/2-Methoxyethanol). ^1^H NMR (DMSOd_6_) 12.29 (br s, 2H, 2NH), 7.86 (br s, 1H, pyrazole proton), 7.47 (dd, 2H, ar, J = 5., 3.2 Hz), 7.12 (dd, 2H, ar, J = 5.8, 3.1 Hz), 7.02 (s, 1H, pyrazole proton). Elemental analysis calcd for C_11_H_9_N_5_O: C, 58.14; H, 3.99; N, 30.82; found C, 58.22; H, 4.07; N, 30.89.

N-(5(6)-Methyl-1H-benzimidazol-2-yl)-1H-pyrazole-3(5)-carboxamide (**8**). Yield 56%; mp> 300 °C (2-Methoxyethanol). ^1^H NMR (DMSOd_6_) 12.37 (br s, 2H, 2NH), 7.83 (br s, 1H, pyrazole proton), 7.34 (d, 1H, ar, J = 8.0 Hz), 7.26 (s, 1H, ar), 7.04–6.89 (m, 2H, 1 ar + 1 pyrazole proton), 2.38 (s, 3H, CH_3_). Elemental analysis calcd for C_12_H_11_N_5_O: C, 59.74; H, 4.60; N, 29.03; found C, 59.81; H, 4.66; N, 29.11.

N-(5(6)-Chloro-1H-benzimidazol-2-yl)-1H-pyrazole-3(5)-carboxamide (**9**). Yield 21%; mp > 300 °C (2-Methoxyethanol). ^1^H NMR (DMSOd_6_) 13.60 (br s, 1H, NH), 12.42 (br s, 1H, NH), 11.50 (br s, 1H, NH), 7.89 (br s, 1H, pyrazole proton), 7.55–7.41 (m, 2H ar), 7.14 (d, 1H ar, J = 8.5 and 1.8 Hz), 7.05 (br s, 1H, pyrazole proton). Elemental analysis calcd for C_11_H_8_ClN_5_O: C, 50.49; H, 3.08; N, 26.76; found C, 50.55; H, 3.10; N, 26.79.

1H-Pyrazole-N-(5(6)-trifluoromethyl-1H-benzimidazol-2-yl)-3(5)-carboxamide (**10**). Yield 35%; mp> 300 °C (DMF). ^1^H NMR (DMSOd_6_) 13.62 (br, 1H, NH), 12.64 (br s, 1H, NH), 11.70 (br s, 1H, NH), 7.91 (br s, 1H, ar or pyrazole proton), 7.82 (s, 1H, ar or pyrazole proton), 7.66 (d, 1H, ar, J = 8.3 Hz), 7.46 (d, 1H, ar, J = 8.3 Hz), 7.08 (s, 1H, pyrazole proton). Elemental analysis calcd for C_12_H_8_F_3_N_5_O: C, 48.82; H, 2.73; N, 23.72; found C, 48.88; H, 2.77; N, 23.77.

N-(5(6)-Methoxy-1H-benzimidazol-2-yl)-1H-pyrazole-3(5)-carboxamide (**11**). Yield 21%; mp> 300 °C (EtOH/2-Methoxyethanol).^1^H NMR (DMSOd_6_) 13.50 (br s, 1H, NH), 12.08 (br s, 1H, NH), 7.85 (br s, 1H, pyrazole proton), 7.35 (d, 1H, ar, J = 8.4 Hz), 7.02 (br s, 2H, 1ar + 1 pyrazole proton), 6.75 (d, 1H, ar, J = 8.6 Hz), 3.76 (s, 3H, CH_3_). Elemental analysis calcd for C_12_H_11_N_5_O_2_: C, 56.03; H, 4.31; N, 27.22; found C, 56.11; H, 4.38; N, 27.29.

N-(5(6)-Nitro-1H-benzimidazol-2-yl)-1H-pyrazole-3(5)-carboxamide (**12**). Yield 20%; mp > 300 °C (EtOH/DMF). ^1^H NMR (DMSOd_6_) 13.65 (br s, 1H, NH), 12.78 (br s, 1H, NH), 11.90 (br s, 1H, NH), 8.37 (s, 1H, ar), 8.08 (d, 1H, ar, J = 6.6 Hz), 7.91 (br s, 1H, pyrazole proton), 7.64 (d, 1H, ar, J = 8.7 Hz), 7.08 (br s, 1H, pyrazole proton). Elemental analysis calcd for C_11_H_8_N_6_O_3_: C, 48.53; H, 2.96; N, 30.87; found C, 48.59; H, 3.02; N, 30.95.

General procedure for the synthesis of N-(Benzimidazol-2-yl)(pyrazol-3(5)-yl)acetamide derivatives **13**–**26**.

A mixture of 2-(3-pyrazolyl) acetic acid hydrochloride **68** [33] (5 mmol) and triethylamine (5 mmol) in anhydrous DMF (about 2–3 mL) was stirred at r.t. for 15 min under a nitrogen atmosphere. Then, EDCI hydrochloride (7 mmol) and 1-OH-benzotriazole (3 mmol) were added. After 10 min, the commercial benzimidazol-2-amine **38** (2 mmol) or the suitable benzimidazol-2-amino derivatives **39**–**51** (2 mmol) were added, and the mixture stirred at r.t. for several hours (16–96 h). The benzimidazol-2-amine **39**–**43** [34], **44** [35], **47** [34], and **48** [36] were prepared as previously described. The new compounds **45**, **46**, and **49**–**51** were synthesized as described below, i.e., by cyclizing the suitable 1,2-phenylenediamines **57**–**64** with cyanogen bromide.

After cooling at 0 °C, the reaction mixture was diluted with H_2_O (about 25 mL), and the suspended solid was collected by filtration, washed with H_2_O (2–3 mL), dried, and purified by recrystallization. Derivative **16** was purified by column chromatography. In the case of derivatives **14** (R = Me) and **26** (R = SO_2_NHMe), dilution of the reaction mixture with H_2_O did not afford any solid, thus the compounds were isolated by extraction with CH_2_Cl_2_ (30 mL × 3). The organic phase was washed with brine (30 mL), anhydrified (Na_2_SO_4_), and evaporated at reduced pressure to give a solid that was treated with Et_2_O (5–10 mL), collected by filtration, and purified by recrystallization (**14**) or by column chromatography (**26**). To isolate derivative **15**, the crude compound (460 mg) was treated with boiling EtOH (50 mL), and the solid obtained after cooling at r.t. was collected by filtration and recrystallized.

N-(1H-benzimidazol-2-yl)-2-(1H-pyrazol-3(5)-yl)acetamide (**13**). Reaction time: 24 h. Yield 10%; mp = 295–297 °C (2-Methoxyethanol/Et_2_O).^1^H NMR (DMSOd_6_) 12.64 (br s, 1H, NH), 12.04 (br s, 1H, NH), 11.69 (br s, 1H, NH), 7.60 (br s, 1H, pyrazole proton), 7.43 (dd, 2H, ar, J = 5.8, 3.1 Hz), 7.07 (dd, 2H, ar, J = 5.8, 3.1 Hz), 6.23 (s, 1H, pyrazole proton), 3.80 (s, 2H, CH_2_). Elemental analysis calcd for C_12_H_11_N_5_O: C, 59.74; H, 4.60; N, 29.03; found C, 59.78; H, 4.66; N, 29.11.

N-(5(6)-Methyl-1H-benzimidazol-2-yl)-2-(1H-pyrazol-3(5)-yl)acetamide (**14**). Reaction time: 20 h. Yield 21%; mp = 257 °C dec (CH_3_CN). ^1^H NMR (DMSOd_6_) 12.60 (br s, 1H), 11.90 (br s, 1H), 11.62 (br s, 1H), 7.60 (br s, 1H, pyrazole proton), 7.30 (d, 1H, ar, J = 8.0 Hz), 7.23 (s, 1H, ar), 6.90 (d, 1H, ar, J = 8.0 Hz), 6.22 (s, 1H, pyrazole proton), 3.78 (s, 2H, CH_2_), 2.36 (s, 3H, CH_3_). Elemental analysis calcd for C_13_H_13_N_5_O: C, 61.17; H, 5.13; N, 27.43; found C, 60.98; H, 5.22; N, 27.51.

N-(5(6)-Cloro-1H-benzimidazol-2-yl)-2-(1H-pyrazol-3(5)-yl)acetamide (**15**). Reaction time: 16 h. Yield 40%; mp = 286–288 °C (2-Methoxyethanol/EtOAc). ^1^H NMR (DMSOd_6_) 12.65 (br s, 1H, NH), 12.23 (br s, 1H, NH), 11.81 (br s, 1H, NH), 7.61 (br s, 1H, pyrazole proton), 7.47–7.43 (m, 2H, ar, J = 8.4 Hz), 7.10 (d, 1H, ar, J = 8.4 Hz), 6.23 (s, 1H, pyrazole proton), 3.81 (s, 2H, CH_2_). Elemental analysis calcd for C_12_H_10_ClN_5_O: C, 52.28; H, 3.66; N, 25.40; found C, 52.37; H, 3.71; N, 25.46.

2-(1H-Pyrazol-3(5)-yl)-N-(5(6)-trifluoromethyl-1H-benzimidazol-2-yl)acetamide (**16**). Reaction time: 16 h. Purified on column chromatography (eluent CH_2_Cl_2_ 9.4/MeOH 0.6). Yield 38%; mp = 275–277 °C. ^1^H NMR (DMSOd_6_) 12.65 (br s, 1H, NH), 12.45–12.43 (m, 1H, NH), 11.92 (br s, 1H, NH), 7.79–7.59 (m, 3H, 2ar + pyrazole proton), 7.42 (d, 1H, ar, J = 8.2 Hz), 6.24 (s, 1H, pyrazole proton), 3.81 (br s, 2H, CH_2_). Elemental analysis calcd for C_13_H_10_F_3_N_5_O: C, 50.49; H, 3.26; N, 22.65; found C, 50.56; H, 3.33; N, 22.70.

N-(5(6)-Methoxy-1H-benzimidazol-2-yl)-2-(1H-pyrazol-3(5)-yl)acetamide (**17**). Reaction time: 16 h.Yield 15%; mp = 239–240 °C (EtOH/2Methoxyethanol). ^1^H NMR (DMSOd_6_) 12.62 (br s, 1H, NH), 11.88 (br s, 1H, NH), 11.61 (br s, 1H, NH), 7.60 (br s, 1H, pyrazole proton), 7.30 (d, 1H, ar, J = 8.6 Hz), 6.99 (s, 1H, ar), 6.70 (d, 1H, ar, J = 8.6 Hz), 6.22 (s, 1H, pyrazole proton), 3.78 (s, 2H, CH_2_), 3.74 (s, 3H, CH_3_). Elemental analysis calcd for C_13_H_13_N_5_O_2_: C, 57.56; H, 4.83; N, 25.82; found C, 57.64; H, 4.98; N, 25.91.

N-(5(6)-Nitro-1H-benzimidazol-2-yl)(1H-pyrazol-3(5)-yl)-2-acetamide (**18**). Reaction time: 96 h. Yield 25%; mp = 272–274 °C (EtOH/DMF). ^1^H NMR (400 MHz, DMSOd_6_) 12.69 (br s, 1H, NH); 12.11 (br s, 1H, NH), 8.33 (s, 1H, ar), 8.06 (dd, 1H, ar, J = 8.8 and 2.2 Hz), 7.61–7.59 (m, 2H, 1 ar + pyrazole proton), 6.25 (s, 1H, pyrazole proton), 3.87–3.85 (m, 2H CH_2_). Elemental analysis calcd for C_12_H_10_N_6_O_3_: C, 50.35; H, 3.52; N, 29.36; found C, 50.41; H, 3.58; N, 29.40.

N-(4(7)-Nitro-1H-benzimidazol-2-yl)(1H-pyrazol-3(5)-yl)-2-acetamide (**19**). Reaction time: 72 h. Yield 25%; mp = 280–282 °C (EtOH). ^1^H NMR (DMSOd_6_) 12.75–12.76 (m, 1H. NH), 12.37–12.28 (m, 1H, NH), 12.00 (s, 1H, NH), 8.01–7.99 (m, 2H, ar), 7.67 (br s, 1H, pyrazole proton), 7.25–7.25 (two triplets, 1H, ar), 6.25 (s, 1H, pyrazole proton), 3.87–3.85 (s, 2H, CH_2_). Elemental analysis calcd for C_12_H_10_N_6_O_3_: C, 50.35; H, 3.52; N, 29.36; found C, 50.44; H, 3.61; N, 29.44.

N-(5(6)-terButyl-1H-benzimidazol-2-yl)(1H-pyrazol-3(5)-yl)-2-acetamide (**20**). Reaction time: 24 h. Yield 20%; mp = 269–264 °C (acetonitrile). ^1^H NMR (DMSOd_6_) 12.61 (br s, 1H, NH), 11.87 (br s, 1H, NH), 7.62 (br s, 1H, ar or pyrazole proton), 7.46 (br s, 1H, ar or pyrazole proton), 7.34 (d, 1H, ar, J = 8.1 Hz), 7.16 (d, 1H, ar, J = 8.1 Hz), 3.79 (s, 2H, CH_2_), 1.32 (s, 9H, 3CH_3_). Elemental analysis calcd for C_16_H_19_N_5_O: C, 64.63; H, 6.44; N, 23.55; found C, 64.67; H, 6.51; N, 23.59.

2-(1H-Pyrazol-3(5)-yl)-N-(5(6)-trifluoromethoxy-1H-benzimidazol-2-yl)acetamide (**21**). Reaction time: 24 h. Yield 25%; mp = 270–274 °C (EtOH). ^1^H NMR (DMSOd_6_) 12.65 (br s, 1H, NH), 12.28 (br s, 1H, NH), 11.81 (br s, 1H, NH), 7.67 (br s, 1H, pyrazole proton), 7.50 (d, 1H, ar, J = 8.3 Hz), 7.41 (s, 1H, ar), 7.07 (d, 1H, ar, J = 8.3 Hz), 6.23 (s, 1H, pyrazole proton), 3.82 (s, 2H, CH_2_). Elemental analysis calcd for C_13_H_10_F_3_N_5_O_2_: C, 48.01; H, 3.10; N, 21.53; found C, 48.08; H, 3.12; N, 21.55.

N-(5,6-Dichloro-1H-benzimidazol-2-yl)-2-(1H-pyrazol-3(5)-yl)acetamide (**22**). Reaction time: 96 h. Yield 28%; mp > 300 °C (EtOH/2Methoxyethanol). ^1^H NMR (DMSOd_6_) 12.67 (br s, 1H, NH), 12.31 (s, 1H, NH), 11.89 (s, 1H, NH), 7.67–7.63 (m, 3H, 2ar + 1 pyrazole proton), 6.23 (s, 1H, pyrazole proton), 3.81 (s, 2H, CH_2_). Elemental analysis calcd for C_12_H_9_Cl_2_N_5_O: C, 46.47; H, 2.93; N, 22.58; found C, 46.55; H, 3.02; N, 22.63.

N-(5(6)-Cyano-1H-benzimidazol-2-yl)(1H-pyrazol-(3)5-yl)-2-acetamide (**23**). Reaction time: 24 h. Yield 19%; mp = 267–268 °C (CH_3_CN). ^1^H NMR (DMSOd_6_) 12.67 (br s, 1H, NH), 12.56 (br s, 1H, NH), 11.97 (br s, 1H, NH), 7.91–7.84 (br s, 1H, ar), 7.68–7.54 (m, 2H, 1 pyrazole proton + 1ar), 7.45 (d, 1H, ar, J = 8.0 Hz), 6.24 (s, 1H, pyrazole proton), 3.83 (s, 2H, CH_2_). Elemental analysis calcd for C_13_H_10_N_6_O: C, 58.64; H, 3.79; N, 31.56; found C, 58.72; H, 3.85; N, 31.59.

2-(2–1H-Pyrazol-3(5)-yl)acetamido)-1H-benzimidazole-5(6)-carboxamide (**24**). Reaction time: 24 h. Yield 20%; mp = 290–292 °C (EtOH/2Methoxyethanol).^1^H NMR (DMSOd_6_) 12.64 (s, 1H, NH), 12.26 (s, 1H, NH), 11.78 (s, 1H, NH), 8.00 (s, 1H, ar), 7.87 (br s, 1H, carboxamide proton), 7.67 (d, 1H, ar, J = 1.3 Hz), 7.65 (d, 1H, ar, J = 7.7 Hz), 7.44 (br s, 1H, carboxamide proton), 7.15 (s, 1H, pyrazole proton), 6.24 (s, 1H, pyrazole proton), 3.81 (br s, 2H, CH_2_). Elemental analysis calcd for C_13_H_12_N_6_O_2_: C, 54.93; H, 4.25; N, 29.56; found C, 55.07; H, 4.31; N, 29.59

2-(1H-Pyrazol-3(5)-yl)-N-(5(6)-sulfamoyl-1H-benzimidazol-2-yl)acetamide (**25**). Reaction time: 72 h. Yield 18%; mp = 285–287 °C (EtOH/2Methoxyethanol). ^1^H NMR (DMSOd_6_) 12.66 (br s, 1H, NH), 12.47 (br s, 1H, NH), 11.91 (br s, 1H, NH), 7.95 (br s, 1H, ar), 7.67 (br s, 1H, pyrazole proton), 7.61–7.54 (m, 2H, ar), 7.21 (br s, 2H, NH_2_), 6.24 (br s, 1H, pyrazole proton), 3.81 (br s, 2H, CH_2_). Elemental analysis calcd for C_12_H_12_N_6_O_3_S: C, 44.99; H, 3.78; N, 26.24; found C, 45.13; H, 3.81; N, 26.33.

N-(5(6)-(N-Methylsulfamoyl-1H-benzimidazol-2-yl)-2-(1H-pyrazol-3(5)-yl)-acetamide (**26**). Reaction time: 48 h. Purified on column chromatography (eluent CH_2_Cl_2_ 9/MeOH 1).Yield 10%; mp = 238–240 °C. ^1^H NMR (DMSOd_6_) 12.57 (br s, 1H, NH), 12.42 (br s, 1H, NH), 11.85, 7.86 (br s, 1H ar), 7.63–7.52 (m, 3H, 2 ar + pyrazole proton), 7.20 (br s, 2H, NH_2_), 6.24 (s, 1H, pyrazole proton), 3.81 (s, 2H, CH_2_), 2.39 (s, 3H, CH_3_). Elemental analysis calcd for C_13_H_14_N_6_O_3_S: C, 46.70; H, 4.22; N, 25.14; found C, 46.75; H, 4.28; N, 25.21.

General procedure for the synthesis of N-(Benzimidazol-2-yl)(pyrazol-3(5)-yl)acetamide derivatives **27**–**29**.

A mixture of 2-(3-pyrazolyl) acetic acid hydrochloride **68** [33] (5.6 mmol) and triethylamine (5.6 mmol) in anhydrous DMF (about 2–3 mL) was stirred at r.t. for 15 min under nitrogen atmosphere. Then, EDCI hydrochloride (7 mmol) and 1-OH-benzotriazole (3.5 mmol) were added. After 10 min, the suitable 2-amino-benzimidazole derivative hydrochlorides **76**, **77** [42], or **78** (4.2 mmol) were added, and the mixture was stirred at r.t. for about 16 h. After cooling at 0 °C, the mixture was diluted with H_2_O (about 25 mL), and the suspended solid was collected by filtration, washed with H_2_O (2–3 mL), dried, and purified by recrystallization.

N-(5(6)-Acetamido-1H-benzimidazol-2-yl)(1H-pyrazol-3(5)-yl)acetamide (**27**). Yield 12%; (EtOH). ^1^H NMR (DMSOd_6_) 12.65 (br s, 1H, NH), 11.97 (br s, 1H, NH), 11.64 (br s, 1H, NH), 9.84 (br s, 1H, NH acetamide), 7.85 (s, 1H, ar), 7.62 (br s, 1H, ar or pyrazole proton), 7.31 (d, 1H, ar, J = 8.5 Hz), 7.17 (br s, 1H, ar or pyrazole proton), 6.22 (s, 1H, pyrazole proton), 3.78 (s, 2H, CH_2_), 2.03 (s, 3H, CH_3_). Elemental analysis calcd for C_14_H_14_N_6_O_2_: C, 56.37; H, 4.73; N, 28.17; found C, 56.45; H, 4.86; N, 28.24.

N-(2-(2-(1H-Pyrazol-3-yl)acetamido)-1H-benzimidazol-3(5)-yl)benzamide (**28**). Yield 20%; mp = 292–293 °C (EtOH/2-Methoxyethanol). ^1^H NMR (DMSOd_6_) 12.58 (br s, 1H, NH), 11.98 (br s, 1H, NH), 11.58 (br s, 1H, NH), 10.12 (br s, 1H, NH benzamide), 7.98–8.01 (m, 3H, ar), 7.54–7.53 (m, 4H, 3ar + 1 pyrazole proton), 7.41–7.39 (m, 2H, ar), 6.24 (s, 1H, pyrazole proton), 3.81 (s, 2H, CH_2_). Elemental analysis calcd for C_19_H_16_N_6_O_2_: C, 63.32; H, 4.48; N, 23.32; found C, 63.37; H, 4.56; N, 23.41.

N-(2-(2-(1H-Pyrazol-3-yl)acetamido)-1H-benzimidazol-4(7)-yl)benzamide (**29**). Yield 28%; mp = 280 °C dec (EtOH). ^1^H NMR (DMSOd_6_) 12.63 (br s, 1H, NH), 12.17 (br s, 1H, NH), 11.89 (br s, 1H, NH), 10.57 (s, 1H, NH benzamide), 7.99 (br s, 2H, ar), 7.64–7.54 (m, 5H, 4ar + 1 pyrazole proton), 7.29 (d, 1H, ar, J = 7.6 Hz), 7.12 (br s, 1H, ar), 6.22 (s, 1H, pyrazole proton), 3.81 (s, 2H, CH_2_). Elemental analysis calcd for C_19_H_16_N_6_O_2_: C, 63.32; H, 4.48; N, 23.32; found C, 63.39; H, 4.54; N, 23.43.

General procedure for the synthesis of the 5(6)-heterocyclic-substituted N-(1H-benzimidazol-2-yl)(1H-pyrazol-3(5)-yl)acetamides **30** and **31**.

A mixture of 2-(3-pyrazolyl)acetic acid hydrochloride **68** [33] (5 mmol) and triethylamine (5 mmol) in anhydrous DMF (about 2–3 mL) was stirred at r.t. for 15 min under a nitrogen atmosphere. Then, EDCI hydrochloride (7 mmol) and 1-OH-benzotriazole (3 mmol) were added. The latter was not used when the tetrazol-1-yl-benzimidazol-2-amine **81** was reacted. After 10 min, the suitable benzimidazol-2-amine **80** or **81** [44] (2 mmol) was added, and the mixture was stirred at r.t. for 72 h, or heated at 60 °C for 48 h, to obtain, respectively, **30** and **31**. After cooling at 0 °C, the mixture was diluted with H_2_O (about 25 mL) and, in the case of **30**, a solid precipitate that was collected by filtration, washed with H_2_O (2–3 mL), dried, and purified by recrystallization. In the case of compound **31**, dilution of the reaction mixture with H_2_O did not afford a solid, but the compound was isolated after extraction with EtOAc (20 mL × 3). The organic phase was washed with brine (30 mL), anhydrified (Na_2_SO_4_), and evaporated at reduced pressure to give a solid that was treated with Et_2_O (5–10 mL) and collected by filtration and purified by recrystallization.

N-(5(6)-(1H-1,2,4-triazol-1-yl)-1H-benzimidazol-2-yl)(1H-pyrazol-3(5)-yl)acetamide (**30**). Yield 25%; mp = 269–271 °C (EtOAc). ^1^H NMR (DMSOd_6_) 12.63 (br s, 1 H, NH), 12.29 (br s, 1 H, NH), 11.83 (br s, 1H, NH), 9.20 (br s, 1H, triazole proton), 8.20 (s, 1H, triazole proton), 7.88 (s, 1H, ar), 7.67–7.57 (m, 3H, 2ar + pyrazole proton), 7.58 (s, 2H, ar), 6.25 (s, 1H, pyrazole proton), 3.82 (s, 2H, CH_2_). Elemental analysis calcd for C_14_H_12_N_8_O: C, 54.54; H, 3.92; N, 36.35; found C, 54.61; H, 3.99; N, 36.41.

N-(5(6)-(1H-tetrazol-5-yl)-1H-benzimidazol-2-yl)(1H-pyrazol-3(5)-yl)acetamide (**31**). Yield 18%; mp > 300 °C. ^1^H NMR (DMSOd_6_) 12.60 (b s, 1H, NH), 12.45 (br s, 1H, NH), 11.88 (br s, 1H, NH), 8.16 (b s, 1 H, ar), 7.81 (s, 1 H, ar), 7.63 (br s, 2H, 1ar + pyrazole proton), 6.25 (s, 1H, pyrazole proton), 3.83 (s, 2H, CH_2_). Elemental analysis calcd for C_13_H_11_N_9_O: C, 50.48; H, 3.58; N, 40.76; found C, 50.52; H, 3.64; N, 40.85.

N-(6-Chloro-1-methyl-benzimidazol-2-yl)(1H-pyrazol-3(5)-yl)acetamide (**32**). A mixture of 2-(3-pyrazolyl)acetic acid hydrochloride **68** [33] (2.2 mmol) and triethylamine (2.2 mmol) in anhydrous DMF (about 2–3 mL) was stirred at r.t. for 15 min under a nitrogen atmosphere. Then, EDCI hydrochloride (3 mmol), 1-OH-benzotriazole (1.3 mmol), and after 10 min, the suitably synthesized 6-chloro-1-methyl-benzimidazol-2-amine **84** (1.5 mmol) were added. The mixture was stirred for 30 h, and then, further, compound **68** (0.7 mmol) and EDCI hydrochloride (1.4 mmol) were added. After 42 h, the mixture was cooled at 0 °C and diluted with H_2_O (about 25 mL). The precipitated solid was collected by filtration and purified on column chromatography (eluent CH_2_Cl_2_ 9/MeOH 1). Yield 15%; mp = 239–240 °C. ^1^H NMR (DMSOd_6_) 12.61 (br s, 1H, NH), 10.85 (br s, 1H, NH), 7.64 (br s, 2H, ar), 7.51 (br s, 1H, pyrazole proton), 7.21 (d, 1H, ar, J = 8.2 Hz), 6.20 (s, 1H, pyrazole proton), 3.76 (s, 2H, CH_2_), 3.57 (s, 3H, CH_3_). Elemental analysis calcd for C_13_H_12_ClN_5_O: C, 53.89; H, 4.17; N, 24.17; found C, 53.97; H, 4.21; N, 24.24.

General procedure for the synthesis of ethyl 1-(2-aryl-2-oxoethyl)-1H-pyrazole-3-carboxylates (**34**–**35**).

Commercially available ethyl 1H-pyrazole-3-carboxylate **33** (6.0 mmol) and the suitable 1-aryl-2-bromoethanone (6.6 mmol) were reacted in anhydrous CH_3_CN (60 mL) and in the presence of K_2_CO_3_ (7.2 mmol) at r.t. for 16 h. The solid was filtered off, and the mother solution was evaporated at reduced pressure to give a solid, which was collected by filtration. Compound **34** [32] was recrystallized from cyclohexane/EtOAc, and **35** was purified by column chromatography (eluent cyclohexane/EtOAc 6:4).

Ethyl 1-(2-oxo-2-phenylethyl)-1H-pyrazole-3-carboxylate (**34**). Yield 90%; mp = 98–100 °C. ^1^H NMR (DMSOd_6_) 8.05 (d, 2H, ar, J = 7.3 Hz), 7.86 (d, 1H, pyrazole proton, J = 2.3 Hz), 7.73 (t, 1H, ar, J = 7.4 Hz), 7.61 (t, 2H, ar, J = 7.7 Hz), 6.82 (d, 1H, pyrazole proton, J = 2.3 Hz), 6.00 (s, 2H, CH_2_), 4.27 (q, 2H, CH_2_, J = 7.1 Hz), 1.29 (t, 3H, CH_3_, J = 7.1 Hz). Elemental analysis calcd for C_14_H_14_N_2_O_3_: C, 65.11; H, 5.46; N, 10.85; found C, 65.28; H, 5.68; N, 10.67.

Ethyl 1-(2-(2-methoxyphenyl)-2-oxoethyl)-1H-pyrazole-3-carboxylate (**35**).Yield 80%; mp = 130–134 °C. ^1^H NMR (DMSOd_6_) 7.87 (d, 1H, pyrazole proton, J = 2.2 Hz), 7.76 (dd, 1H ar, J = 7.8, 1.4 Hz), 7.65 (t, 1H, ar, J = 7.5 Hz), 7.27 (d, 1H, ar, J = 8.4 Hz), 7.10 (t, 1H, ar, J = 7.5 Hz), 6.79 (d, 1H, pyrazole proton, J = 2.2 Hz), 5.73 (s, 2H, CH_2_), 4.27 (q, 2H, CH_2,_ J = 7.1 Hz), 3.99 (s, 3H, OCH_3_), 1.29 (t, 3H, CH_3_, J = 7.1 Hz). Elemental analysis calcd for C_15_H_16_N_2_O_4_: C, 62.49; H, 5.59; N, 9.72; found C, 62.68; H, 5.78; N, 9.51.

General procedure for the synthesis of 1-(2-aryl-2-oxoethyl)-1H-pyrazole-3-carboxylic acids **36**–**37**.

A total of 1M NaOH solution (95 mL) was added dropwise at 0 °C to a cooled (T = 0 °C) solution of the ester **34** or **35** (19 mmol) in EtOH (200 mL). The mixture was heated at 50 °C for 2 h and then concentrated at about one-third of its volume by evaporation of the solvent at reduced pressure. Then, the solution was diluted with about the same volume of H_2_O and acidified to pH = 2 with 6N HCl. The yellow solid that precipitated was collected by filtration, washed with water, and dried. The compounds were pure enough to be used for the next step without further purification.

1-(2-Oxo-2-phenylethyl)-1H-pyrazole-3-carboxylic acid (**36**). Yield 74%; mp = 197–200 °C. ^1^H NMR (DMSOd_6_) 12.66 (br s, 1H, OH) 8.05 (d, 2H, ar, J = 7.4 Hz), 7.83 (d, 1H, pyrazole proton, J = 2.3 Hz), 7.73 (t, 1H, ar, J = 7.4 Hz), 7.60 (t, 2H, ar, J = 7.7 Hz), 6.76 (d, 1H, pyrazole proton, J = 2.3 Hz), 5.96 (s, 2H, CH_2_).

1-(2-(2-Methoxyphenyl)-2-oxoethyl)-1H-pyrazole-3-carboxylic acid (**37**). Yield 65%; mp = 188–191 °C. ^1^H NMR (DMSOd_6_) 12.66 (s, 1H, OH), 8.05 (d, 7.83 (d, 1H, pyrazole proton, J = 2.2 Hz), 7.75 (dd, 1H, ar, J = 7.7, 1.5 Hz), 7.69–7.62 (m, 1H ar), 7.27 (d, 1H, ar, J = 8.4 Hz), 7.10 (t, 1H, ar, J = 7.5 Hz), 6.74 (d, 1H, pyrazole proton, J = 2.2 Hz), 5.70 (s, 2H, CH_2_), 3.98 (s, 3H, CH_3_).

5-(6)Terbutyl-1H-benzimidazol-2-amine (**45**). BrCN (3.37 mmol) was added to a solution of commercially available 4-tertbutyl-1,2-phenylendiamine **58** (3.04 mmol) in a 1:1 mixture of H_2_O and MeOH (50 mL), and the mixture was heated at 60 °C for 5 h. About half of the solvent was eliminated under vacuum, and the mixture was extracted with EtOAc (50 mL × 5). Evaporation at reduced pressure gave a red oil, which was treated with H_2_O (10 mL). The mixture was basified to pH = 8–9 with a NaHCO_3_-saturated solution to obtain a solid, which was collected by filtration and used for the next step without further purification. Yield 45%. mp = 201–203 °C. ^1^H NMR (DMSOd_6_) 10.50 (br s, 1H, NH), 7.11 (s, 1 H, ar), 7.00 (d, 1H, ar, J = 7.6 Hz); 6.91 (s, 1 H, ar, J = 7.6 Hz), 6.01 (s, 2H, NH_2_), 1.29 (s, 9 H, 3CH_3_). Elemental analysis calcd for C_11_H_15_N_3_: C, 69.81; H, 4.65; N, 10.76; found C, 62.23; H, 4.37; N, 10.51.

5-(6)Trifluoromethoxy-1H-benzimidazol-2-amine (**46**). BrCN (19.7 mmol) was added to a solution of 4-trifluoromethoxy-1,2-phenylenediamine **59** [37] (19.5 mmol) in H_2_O (40 mL) and CH_3_CN (5 mL). The mixture was heated at reflux for 4 h, and after the addition of a second crop of BrCN (0.9 mmol), it was left at r.t. overnight. After the elimination of about half of the solvent at reduced pressure, the solution was basified to pH = 8 with NaHCO_3_-saturated solution. The obtained solid was collected by filtration and used for the next step without further purification. Yield 88%. ^1^H NMR (DMSOd_6_) 10.85 (s, 1H, NH), 7.11 (d, 1H, ar, J = 8.4 Hz), 7.03 (s, 1H, ar), 6.8 (d, 1H, ar, J = 7.5 Hz), 6.37 (s, 2H, NH_2_).

2-Amino-1H-benzimidazole-5(7)-carboxamide (**49**). BrCN (3.37 mmol) was added to a solution of 3,4-diaminobenzamide **62** [38] (2.25 mmol) in MeOH (15 mL). The mixture was stirred at r.t. overnight, then the second portion of BrCN (1.12 mmol) was added. After 3 h, the solvent was eliminated under vacuum, the obtained solid was dissolved in H_2_O (8 mL), and the solution was basified to pH = 9 with a NaHCO_3-_saturated solution. A solid slowly precipitated, and after about 10 h, it was collected by filtration and recrystallized from acetonitrile. Yield 35%; mp = 294–295 °C. ^1^H NMR (DMSOd_6_) 10.89 (br s, 1H, NH), 7.75 (br s, 1H, CONH_2_), 7.67 (s, 1H, ar), 7.49 (d, 1H, ar, J = 8.2 Hz), 7.09 (d, 1H, ar, J = 8.2 Hz), 7.02 (s, 1 H, CONH_2_), 6.44 (s, 2 H, NH_2_). Elemental analysis calcd for C_8_H_8_N_4_: C, 54.54; H, 4.58; N, 31.80; found C, 54.38; H, 4.42; N, 31.69.

General procedure for the synthesis of 2-amino-1H-benzimidazole-5(7)-sulfonamide (**50**) and 2-amino-N-methyl-1H-benzimidazole-5(7)-sulfonamide (**51**).

The title compounds were synthesized by reacting the suitable 3,4-diaminobenzensulphonamide **63** [39] or **64** (5.34 mmol) with BrCN (5.34 mmol) in a 1:1 mixture of H_2_O and MeOH (60 mL) heated at 50 °C for 5 h. After evaporation of about half the solvent, the mixture was basified to pH = 8 with NaHCO_3-_saturated solution, and the suspension was extracted with EtOAc (30 mL × 3). The organic phase was anhydrified (Na_2_SO_4_), and the solvent evaporated at reduced pressure to give a solid (**50**), which was recrystallized from MeCN, or an oily residue (**51**), which was used as such for the next step.

Compound **50**. Yield 75%; m.p. 238–240 °C. ^1^H NMR (DMSOd_6_) 11.03 (s, 1H, NH), 7.55 (s, 1 H, ar), 7.37 (d, 1H, ar, J = 8.1 Hz), 7.17 (d, 1H, ar, J = 8.1 Hz), 7.05 (s, 2H, NH_2_), 6.52 (s, 2H, NH_2_). Elemental analysis calcd for C_7_H_8_N_4_O_2_S: C, 39.62; H, 3.80; N, 26.40; found C, 39.41; H, 3.99; N, 26.18.

Compound **51**. Yield 90%. ^1^H NMR (DMSOd_6_) 11.05 (br s, 1H, NH), 7.49 (d, 1H, ar, J1.6 Hz), 7.31 (d, 1H, ar, J = 1.6 and 8.2 Hz), 7.21 (d, 1H, ar, J = 8.2 Hz),7.07 (q, 1 H, NH, J = 5.1 Hz), 6.55 (s, 2H, NH_2_), 2.34 (d, 3H, CH_3_, J = 5.1 Hz).

3,4-Diamino-N-methylbenzensulphonamide (**64**). Pd/C (10%, 0.09 g) was added to a solution of 4-amino-N-methyl-3-nitrobenzenesulphonamide **65** (3.91 mmol) in EtOH (150 mL), and the mixture was hydrogenated in a Parr apparatus at 35 psi for 15 h. The catalyst was filtered off, and the solvent was evaporated at reduced pressure to give an oil that solidified spontaneously. The compound was pure enough to be used in the next step without further purification. Yield 80%. ^1^H NMR (DMSOd_6_) 6.90 (d, 1 H, ar, J = 2.1 Hz); 6.83–6.79 (m, 2H, 1 ar + NH), 6.56 (d, 1H, ar, J = 8.1 Hz), 5.20 (s, 2H, NH_2_), 4.83 (s, 2H, NH_2_), 2.33 (d, 3 H, CH_3_, J = 5.2 Hz).

General procedure for the synthesis of nitro-substituted 1-tert-butoxycarbonyl-2-(N,N-bis-tert-butoxycarbonylamino)-benzimidazole derivatives **69** and **70**.

The new compound **70** was prepared in the same conditions described for **69** [41]. Briefly, di-tert-butyl dicarbonate (16.8 mmol) was added to a suspension of the suitable 2-aminobenzimidazole derivative **43** [34] or **44** [35] (5.6 mmol) in anhydrous THF (55 mL). The mixture was stirred at r.t. for 5 h, then small amounts of impurities were filtered off, and a second crop of di-tert-butyl dicarbonate (16.8 mmol) was added to the filtrate, followed by 4-dimethylaminopyridine (0.6 mmol). The mixture was stirred at r.t. overnight, then the solvent was evaporated at reduced pressure to give a solid (**70**), which was collected and used for the next step, or a brownish oil (**69**), which solidified on air and was purified by silica gel column chromatography (eluted with a gradient of n-hexane/EtOAc from 10:1 to 5:2). Compounds **69** and **70** were a mixture of two regioisomers (**69**, 5-and 6-nitro substituted; **70**, 4- and 7-nitro substituted).

1-Tert-butoxycarbonyl-2-(N,N-bis-tert-butoxycarbonylamino)-5- and 6-nitrobenzimidazole (**69**). Yield 54%. ^1^H NMR (CDCl_3_), the ratio of the two isomers was about 9:1 (from ^1^H-NMR spectrum of the crude). Isomer I, more abundant, 8.67 (s, 1H, ar), 8.35 (d, 1H, ar, J = 7.0 Hz), 8.17 (d, 1H, ar, J = 9.1 Hz), 1.71 (s, 9H, CH_3_), 1.50 (s, 18H, CH_3_). Isomer II, less abundant, 8.93 (s, 1H, ar), 8.26 (d, 1H, 8.8 Hz), 7.84 (d, 1H; ar, J = 8.8 Hz), 1.76 (s, 9H, CH_3_), 1.40 (s, 18H, CH_3_).

1-Tert-butoxycarbonyl-2-(N,N-bis-tert-butoxycarbonylamino)-4(7)-nitrobenzimidazole (**70**). Yield 70%. The ratio of the two isomers was about 7:1 (from ^1^H-NMR spectrum of the crude). ^1^H NMR (DMSOd_6_) of the more abundant isomer: 8.40 (d, 1H, J = 8.3 Hz), 8.25 (d, 1H, ar, J = 8.2 Hz), 7.73 (t, 1H, ar, J = 8.3 Hz), 1.65 (s, 9H, CH_3_), 1.39 (s, 18H, CH_3_). ^1^H NMR (DMSOd_6_) of the less abundant isomer: 8.12 (d, 1H, ar, J = 8.2 Hz), 8.08 (d, 1H, ar, J = 8.2 Hz), 7.48 (t, 1H, ar, J = 8.2 Hz), 1.54 (s, 9H, CH_3_), 1.49 (s, 18H, CH_3_).

General procedure for the synthesis of amino-substituted 1-tert-Butoxycarbonyl-2-(N,N-bis-tert-butoxycarbonylamino)-benzimidazole derivatives **71** and **72**.

The newly synthesized compound **72** was prepared as previously described for **71** [41]. Briefly, compounds **69** or **70** (4.1 mmol), both used as a mixture of the two isomers, were suspended with 10% Pd/C (0.2 g) in MeOH (100 mL), and the mixture was hydrogenated in a Parr apparatus at 35 psi for 8 h. The catalyst was filtered off, and the solvent was evaporated to give a residue that was taken up with a few Et_2_O (2–3 mL) and collected by filtration. Each amino derivative, **71** and **72**, was constituted by one compound, since the less abundant isomer was lost in the mother liquor. Both derivatives, **71** and **72**, were pure enough to be used for the next step without further purification. We did not determine whether the compounds were 5- or 6-substituted, because their subsequent deprotection generated one isomeric product.

1-Tert-butoxycarbonyl-2-(N,N-bis-tert-butoxycarbonylamino)-5- or 6-aminobenzimidazole (**71**). Yield 87%. ^1^H NMR (CDCl_3_) 7.77 (d, 1H, ar, J = 8.7 Hz), 7.03 (s, 1H, ar), 6.79 (d, 1H, ar, J = 8.7 Hz), 3.73 (s, 2H, NH_2_), 1.67 (s, 9H, CH_3_), 1.42 (s, 18H, CH_3_).

1-Tert-butoxycarbonyl-2-(N,N-bis-tert-butoxycarbonylamino)-4 or 7-aminobenzimidazole (**72**).Yield 75%. ^1^H NMR (DMSOd_6_) 6.86 (d, 1H, J = 7.9 Hz), 6.72 (dd, 1H, ar, J = 8.1, 3.9 Hz), 6.44 (s, 1H, ar, J = 7.7 Hz), 4.80 (s, 2H, NH_2_), 1.64 (s, 9H, CH_3_), 1.41 (s, 18H, CH_3_).

General procedure for the synthesis of 4- or 5-amido substituted 1-tert-Butoxycarbonyl-2-(N,N-bis-tert-butoxycarbonylamino)-benzimidazole derivatives **73**–**75**.

The suitable acyl chloride (5.3 mmol) and triethylamine (5.3 mmol) were added in sequence to a solution of the amino derivative **71** or **72** (4.5 mmol) in anhydrous CH_2_Cl_2_ (20 mL). The mixture was stirred at r.t. overnight and then quenched with H_2_O (20 mL). The organic phase was washed with H_2_O (15 mL × 3) and then anhydrified (Na_2_SO_4_). Evaporation of the solvent at reduced pressure gave a solid, which was collected, dried, and used for the next step without further purification.

Tert-butyl 5-acetamido-2-((N,N-bis-tertbutoxycarbonyl)amino)-1H-benzimidazole-1-carboxylate (**75**). Yield 92%. ^1^H NMR (DMSOd_6_) 10.13 (s, 1H, NH), 8.08 (s, 1H, ar), 7.85 (d, 1H, ar, J = 8.9 Hz), 7.54 (d, 1H, ar, J = 8.9 Hz), 2.08 (s, 3H, CH_3_), 1.62 (s, 9H, CH_3_), 1.35 (s, 18H, CH_3_).

Tert-butyl 5-benzamido-2-((N,N-bis-tertbutoxycarbonyl)amino)-1H-benzimidazole-1-carboxylate (**76**). Yield 90%. ^1^H NMR (CDCl_3_) 8.02–7.92 (m, 4H), 7.73 (d, 1H, ar, J = 8.7 Hz), 7.59–7.51 (m, 3H), 1.69 (s, 9H, CH_3_), 1.43 (s, 18H, CH_3_).

Tert-butyl 4-benzamido-2-((N,N-bis-tert-butoxycarbonyl)amino)-1H-benzimidazole-1-carboxylate (**77**). Yield 86%. ^1^H NMR (DMSOd_6_) 10.15 (s, 1H, NH), 8.15 (d, 2H, ar), 8.03 (d, 1H, ar), 7.92 (d, 1H, ar), 7.64–7.47 (m, 4H), 1.64 (s, 9H, CH_3_), 1.38 (s, 18H, CH_3_).

General procedure for the synthesis of 4- or 5-amido substituted 2-aminobenzimidazole hydrochlorides **76**–**78**.

A solution of the suitable BoC-derivatives **73**–**75** (4.0 mmol) in trifluoroacetic acid (2 mL) and CH_2_Cl_2_ (2 mL) was stirred at r.t. overnight. Evaporation of the solvents at reduced pressure gave an oily residue, which was taken up with a saturated methanol solution of hydrochloric acid (2 mL). The solution was stirred at r.t. for 30 min, then the solvent was eliminated at reduced pressure, yielding the desired benzimidazol-2-amine hydrochlorides.

5-Acetamido-2-amino-1H-benzimidazole hydrochloride (**76**) [42]. Yield 96%. ^1^H NMR (DMSOd_6_) 12.28 (br s, 2H), 10.06 (s, 1H, NHCO), 8.32 (s, 2H, NH_2_), 7.91 (s, 1H, ar), 7.24–7.22 (m, 2H, ar), 2.05 (s, 3H, CH_3_).

N-(2-Amino-1H-benzimidazol-5-yl)benzamide hydrochloride (**77**) [42]. Yield 100%. ^1^H NMR (DMSOd_6_) 12.53 (br s, 2H), 10.39 (s, 1H, NHCO), 8.46 (s, 2H, NH_2_), 8.03 (s, 1H, ar), 7.96 (d, 2H, ar, J = 8.5 Hz), 7.62–7.54 (m, 4H, ar), 7.33 (d, 1H, ar, J = 8.6 Hz).

N-(2-Amino-1H-benzimidazol-4-yl)benzamide hydrochloride (**78**). Yield 100%. ^1^H NMR (DMSOd_6_) 12.82 (s, 1H), 12.04 (s, 1H), 10.67 (s, 1H, NHCO), 8.41 (s, 2H, NH_2_), 8.07 (d, 2H, ar, J = 8.5 Hz), 7.66–7.50 (m, 3H, ar), 7.40–7.38 (m, 1H, ar), 7.24–7.20 (m, 2H, ar).

5-(1,2,4-Triazol-1-yl)benzimidazol-2-amine (**80**). BrCN (5.70 mmol) was added to a solution of 4-(1,2,4-triazol-1-yl)-1,2-diaminobenzene **79** (2.28 mmol) in a 1:1 mixture of H_2_O and MeOH (50 mL). The mixture was heated at 50 °C for 6 h, then about half of the solvent was evaporated at reduced pressure. After being cooled to room temperature, the mixture was alkalinized to pH = 8 with NaHCO_3_-saturated solution and extracted with EtOAc (25 mL × 10). The organic phase was anhydrified with Na_2_SO_4,_ and the solvent was evaporated at reduced pressure to give an oily residue that crystallized upon treatment with a few drops of Et_2_O. The solid was collected by filtration and recrystallized from EtOAc and a few drops of MeOH. Yield 76%. mp = 128–130 °C. ^1^H NMR (DMSOd_6_) 11.17 (br s, 1 H, NH), 9.13 (s, 1 H, triazole proton), 8.16 (s, 1 H, triazole proton), 7.53 (s, 1 H, ar); 7.34 (d, 1H, ar, J = 8.3 Hz), 7.21 (d, 1H, ar, J = 8.3 Hz); 6.52 (s, 2 H, NH_2_). Elemental analysis calcd for C_9_H_8_N_6_: C, 53.99; H, 4.03; N, 41.98; found C, 53.75; H, 3.99; N, 41.75.

5-Chloro-N1-methylbenzene-1,2-diamine (**83**). 5-Chloro-2-nitro-N-methylaniline **82** [45] (2.68 mmol) was dissolved in boiling ethanol (50 mL), then Raney nickel (2400 slurry in H_2_O, 2 mL) was added, and the mixture was hydrogenated in a Parr apparatus at 15 psi for 2 h. The catalyst was filtered off, and the solvent was evaporated to give an oily residue that was used for the next step. Yield 85%. ^1^H NMR (DMSOd_6_) 6.50 (d, 1H, ar, J = 8.38 Hz), 6.40 (dd, 1 H, ar, J = 8.4 and 2.3 Hz), 6.30 (d, 1H, J = 2.3 Hz), 4.90 (q, 1H, NH, J = 4.9 Hz), 4.58 (s, 2H, NH_2_), 2.69 (d, 3H, CH_3_, J = 4.9 Hz).

6-Chloro-1-methyl-benzimidazol-2-amine (**84**). BrCN (5.58 mmol) was added to a solution of the diamine **83** (2.23 mmol) in a 1:1 mixture of MeOH/H_2_O (40 mL). The solution was heated at 60 °C for 3 h, then about half of the solvent was evaporated at reduced pressure. After being cooled to room temperature, the mixture was neutralized with 1M NaOH. The solid obtained, collected by filtration, was pure enough to be used for the next step. Yield 74%. ^1^H NMR (DMSOd_6_) 7.23 (s, 1H, ar), 7.09 (d, 1H, ar, J = 8.2 Hz), 6.93 (d, 1H, J = 8.2 Hz), 6.52 (s, 2 H, NH_2_), 3.49 (s, 3 H, CH_3_).

### 3.2. CK1δ Activity Assays

The inhibitory activity of synthesized compounds on CK1δ has been carried out following a procedure for a luminescence-based assay (KinaseGlo^®^ kit, Promega Italy Srl, Milan, Italy) already reported in the literature [50,54]. Luminescent assays were performed in white 96-well plates in a 40 μL final volume. The buffer used is a milliq water solution of 50 mM HEPES (pH 7.5), 1 mM EDTA, 1 mM EGTA, and 15 mM MgCl_2_. In a typical assay, 10 μL of inhibitor solution (desired concentrations were obtained by diluting in buffer a starting 10 mM solution in DMSO) and 10 μL of enzyme solution (truncated CK1δ, recombinant human, GST-tagged, aa 1-294, Merck Millipore, Merck KGaA, Darmstadt, Germany) were added to the well, followed by 20 μL of assay buffer containing casein substrate (casein solution from bovine milk, 5% in water, Sigma-Aldrich, Merck KGaA, Darmstadt, Germany) and ATP, at a final concentration of 0.05% and 2 μM, respectively. The final enzyme concentration was 6.5 nM. Compound **PF-670462** (IC_50_ = 4 nM) [24] at a concentration of 40 μM was used as a positive control for CK1δ, and a DMSO/buffer solution was used as a negative control. The final DMSO concentration in the reaction mixture did not exceed 1%. After 60 min of incubation at 30 °C, the enzymatic reactions were stopped with the addition of 40 μL of KinaseGlo reagent (Promega Italy Srl, Milan, Italy). The luminescence signal (relative light unit, RLU) was recorded after 10 min at 25 °C using a Tecan Infinite M100. First, residual enzyme activity percentage was determined in duplicate at 40 μM for each inhibitor with respect to DMSO/buffer only, and IC_50_ values were determined for those compounds showing enzyme residual activity lower than 50% at that concentration. IC_50_ values were evaluated using ten different inhibitor concentrations ranging from 100 to 0.026 μM. Data were analyzed using Excel and GraphPad Prism software (version 8.0).

### 3.3. Molecular Modeling Studies

#### 3.3.1. Hardware and Software Overview

Most of the molecular modeling operations were carried out using the MOE suite [55].

Docking simulations were performed using PLANTS (Protein Ligand ANT System) [56]. The computation of MMFF94 partial charges was conducted through MOPAC [57], which is integrated within the MOE suite.

Analyses of docking poses, including energy calculations, visual inspections, and pharmacophoric filtering, were conducted using the tools available in the MOE suite.

The molecular modeling studies were performed on an 8 CPU Linux workstation (Intel^®^ Xeon^®^ CPU E5-1620 3.70 GHz).

Molecular dynamics simulations were carried out on an in-house Linux GPU cluster composed of 20 NVIDIA devices ranging from a GTX2080Ti to an RTX3090.

#### 3.3.2. Structure Preparation

The three-dimensional structure of the selected complex for analysis was retrieved from the Protein Data Bank [46] (PDB ID: 5OKT) and subsequently prepared using the tools provided by the MOE 2022.2 suite [55]. Upon importing the structure into the MOE environment, the “Structure Preparation” tool was utilized. This step is essential for addressing various issues within the chosen protein chain, which encompass the addition of missing atoms, the capping of N/C-termini, and the management of tautomeric forms and multiple conformations of specific residues. Furthermore, any gaps in the protein structure were reconstructed using a homology-based approach. The determination of protonation states was conducted using MOE’s “Protonate3D” tool, which added hydrogen atoms and assigned ionization states to each residue based on a fixed pH of 7.4. The most probable tautomer was calculated based on hydrogen-bonding contributions that significantly contributed to the structure’s stability. Fractional occupancies of residues were selected based on the highest probability. Finally, partial charges were computed using the AMBER14 force field, and the hydrogen atoms added during the reconstruction phases underwent an energy minimization process using the same force field.

#### 3.3.3. Molecular Docking

The compound database underwent a comprehensive preparation process for computational analysis, utilizing various tools from the OpenEye suite [58]. Firstly, the most probable tautomeric state was assigned to each ligand within the database, by the usage of Tautomers [59]. Following this, three-dimensional coordinates of the various molecules were generated with Omega [60], with a specific focus on minimizing their energy. The protonation states of ionizable groups were then determined with Fixpka [61] to ensure accuracy. Lastly, to facilitate subsequent analysis, partial charges were assigned to the atoms within the ligand database thanks to Molcharge [62].

Molecular docking was performed using PLANTS, with the empirical CHEM-PLP scoring function. The conformational search radius was set at 10 Å relative to the center of mass of the crystallographic ligand (PDB ID: 5OKT).

Following this, atomic partial charges were computed for both ligand poses, using the MMFF94 method, and receptors, using the Amber14EHT force field. The contributions of electrostatic and van der Waals forces to the binding energy were calculated using MOE.

#### 3.3.4. Pharmacophoric Filter

The pharmacophore is a set of crucial features that ensure optimal intermolecular interactions between a ligand and its reference protein structure [63]. Here, a pharmacophoric model was built with MOE using the crystallographic complex 5OKT. This resulted in the features describing the aromatic scaffold of the ligand and a bi-dentate hydrogen bond between the ligand and Leu85. In particular, donor and acceptor features were positioned on the ligand’s 2-aminobenzothiazole NH and N; and acceptor and donor features were positioned on Leu85 carbonyl and amino groups. In all cases, the dimension of the feature sphere was 2 Å.

#### 3.3.5. Interaction Energy Fingerprints (IEF)

Electrostatic interactions were assessed based on the non-bonded energy term of the force field, utilizing the Coulombic function, and they are expressed in kcal/mol. On the other hand, the hydrophobic contribution was calculated using a hydrophobic interaction term associated with a dimensionless score. This calculation is based on the analysis of contact surfaces carried out using MOE 2022.02 software, employing a specific scoring function.

To visually represent the results on a “per residue” basis, Heat Maps were utilized and generated with GNUPLOT. These graphical representations employ specific color scales to visualize the calculated interactions between each amino acid of the molecular target (x-axis) and each ligand (y-axis). For IEele (Electrostatic Interaction Energy), colors ranging from blue to red represent energy values from negative to positive. As for IEhyd (Hydrophobic Interaction Energy), colors from white to dark green depict scores ranging from 0 to positive values.

#### 3.3.6. Docking Video Maker

An internal tool called MMsDocking video maker has been exploited to automatically create a video that displays the most pertinent docking data, including docking poses, per-residue IEhyd and IEele data, and experimental binding data, in order to make the visualization and analysis of the data from the docking simulations easier. After obtaining images using the following method, videos were mounted using MEncoder [64]. The backdrop heat maps were created using GNUPLOT 5.2.8 [65], based on per-residue IEhyd and IEele data that were computed using MOE. Using the open-source RDKit [66] cheminformatics tools, two-dimensional representations of the molecules were produced. CHIMERA [67] was utilized to reconstruct docking pose representations within the binding location.

#### 3.3.7. System Setup for MD Simulations and Equilibration Protocol

All protein–ligand complexes were prepared according to previously described procedures and subsequently processed using Visual Molecular Dynamics (VMD) 1.9.2 [68] and the AmberTools22 package [69,70]. The protein atoms were parameterized employing the ff14SB [71] force field, and the General Amber Force Field (GAFF) [72] was used to parameterize the ligands. Partial charges were assigned to the ligands using the AM1-BCC method [73].

Each investigated system was solvated within a cubic box with a 15 Å buffer, utilizing the TIP3P [74] water model. The appropriate number of sodium and chloride ions was added to neutralize the system and achieve a salt concentration of 0.154 M. Before commencing molecular dynamics (MD) simulations, each system underwent an energy minimization consisting of 500 steps using the conjugate gradient algorithm to remove collisions.

Subsequently, each minimized system underwent a two-phase equilibration protocol. In the first phase, a 0.1 ns simulation was performed in the canonical ensemble (NVT), with harmonic positional restraints applied to both protein and ligand atoms. The second phase consisted of a 0.5 ns simulation in the isothermal–isobaric ensemble (NPT), applying the same restraints only to the ligand’s position and the basic protein structure.

All MD simulations presented in this work, both in the equilibration and production phases, were executed with a 2 fs integration time step, maintaining a constant temperature of 310 K for the SuMD simulations and 300 K for the TTMD simulations, using a Langevin thermostat [75], restraining hydrogen bond lengths through the M-SHAKE [76] algorithm, and calculating long-range electrostatic interactions via the particle mesh Ewald method [77] with cubic spline interpolation. A 9.0 Å cutoff was employed for real-space electrostatic and van der Waals interactions, with a switching distance of 7.5 Å. Simulations in the NPT ensemble were conducted by maintaining constant pressure at 1 atm using a Monte Carlo barostat [78].

All MD simulations were conducted using the ACEMD16 3.5.1 engine, based on the open-source OpenMM 7 library [79].

#### 3.3.8. Supervised Molecular Dynamics (SuMD) Simulations

Supervised Molecular Dynamics (SuMD) is a computational method that allows for the exploration of the ligand–target molecular recognition pathway on a reduced time scale. This process involves a series of unbiased Molecular Dynamics (MD) simulations, followed by an assessment of the simulation’s progress using an algorithm like tabu search. In each of these MD simulations, referred to as “SuMD-steps”, the system is simulated in the canonical ensemble at a constant temperature of 310 K. A 500-picosecond (ps) SuMD-step was employed in this work.

At the end of each SuMD-step, the distance between the center of mass of the ligand and the binding site is calculated along the trajectory, and the distance data are then fitted using a linear function. If the slope of the resulting linear fit is negative, indicating that the ligand is moving closer to the binding site, the “SuMD-step” is deemed productive and retained for generating the final trajectory. The concluding state of the simulation is used as the starting point for the subsequent step. Conversely, if the slope is positive, suggesting that the ligand is not approaching the binding site, the “SuMD-step” is considered unproductive and is thus discarded. In this case, the step is repeated by randomly adjusting particle velocities using the Langevin thermostat, with the final coordinates from the end of the previous “SuMD-step” retained.

The supervision algorithm is deactivated when the distance between the two centers of mass falls below a threshold value (in this instance, 10 Å). Beyond this point, the simulation continues for an additional 10 nanoseconds (ns) of classical molecular dynamics.

Ligand–receptor interaction energy alongside the SuMD trajectory was obtained through the “NAMD Energy” plugin for VMD, which exploits the NAMD [80] package to retrieve an estimate of the interaction energy defined as the sum of the van der Waals and electrostatic contribution calculated according to the force field.

Root Mean Square Deviation (RMSD) of the ligand’s heavy-atom coordinates was computed during the trajectory, using the docking pose as a reference.

Electrostatic interactions were computed on a “Per Residue” basis and plotted in a heatmap displaying time on the x-axis and the 15 most contacted residues on the y-axis and using a color scale to represent the intensity of the interaction, ranging from blue to red for negative to positive values.

All the geometric analyses were performed making use of the MDAnalysis [81,82] Python library; and all the plots were generated with the Matplotlib [83] module.

#### 3.3.9. Thermal Titration Molecular Dynamics (TTMD) Simulations

Thermal Titration Molecular Dynamics (TTMD) is a molecular dynamics protocol developed for the qualitative assessment of protein–ligand unbinding kinetics [53]. The TTMD methodology involves a series of short classic molecular dynamics simulations, termed “TTMD-steps”, conducted at progressively increasing temperatures. The temperature increment serves to increase the kinetic energy of the system, thereby reducing the simulation time required to observe protein–ligand unbinding events compared to conventional molecular dynamics simulations. To track the simulation’s progress, the preservation of the native binding mode is assessed using a scoring function based on the Protein–Ligand Interaction Fingerprint (PLIF) [84].

The following protocol is implemented as a Python 3.10 code, utilizing the NumPy, MDAnalysis [81,82], Open Drug Discovery Toolkit (ODDT) [85], and Scikit-learn packages.

In this case, the initial temperature was set at 300 K, and the simulation was interrupted when the ligand native binding mode and its interaction fingerprint were lost. The temperature increased by 10K between each “TTMD-step”, and the duration of each simulation window was set at 10 ns. The choice of the temperature ramp was based on the preservation of the protein’s native fold throughout the simulation, which was monitored by assessing the backbone Root Mean Square Deviation (RMSD).

#### 3.3.10. Physicochemical and Pharmacokinetic Parameters

Some physicochemical and pharmacokinetic parameters (such as predicted logP, logS, logBB, blockage of HERG K+ channels, Caco-2 cell permeability, binding to human serum albumin, and oral absorption) were computed for the most active compounds, **15**, **18**, **22**, **23** (Table S1 in the Supplemetary Materials), with QikProp of the Schrödinger package [86].

## 4. Conclusions

This work has produced a small library of new benzimidazol-2-amino derivatives (**1**–**32**) designed as ATP-competitive CK1δ inhibitors, which have attracted attention for their potential therapeutic application in cancer and neurodegenerative diseases. Three sets of compounds were synthesized bearing different substituents on the fused benzo ring and diverse pyrazole-containing acyl moieties on the 2-amino group. The best inhibitors proved to be compounds featuring the (1H-pyrazol-3-yl)-acetyl residue on the 2-amino group (derivatives **13**–**32**), since most of them showed inhibitor activity in the low micromolar range. The most advantageous substituent on the fused benzo ring was 5-CN, which afforded nanomolar potency (23, IC_50_ = 98.6 nM), followed by 5-NO_2_ (19, IC_50_ = 0.12 μM), 5-Cl (15, IC_50_ = 0.485 μM), and 5,6-diCl (22, IC_50_ = 0.98 μM).

SAR analysis highlighted the structural requirements for targeting CK1δ. Molecular modeling studies at the CK1δ crystal structure were performed to rationalize the observed activity data. Molecular docking and molecular dynamics simulations (specifically SuMD and TTMD) were used to compare the most potent compound (23, IC_50_ = 98.6 nM) with 5-CN and the unsubstituted analogue (13, IC_50_ = 4.21 μM), and the analysis ascribed the potency increase to interaction between the cyano group and two crystallographic water molecules in the binding site. These results pointed out that both protein–ligand and solvent-mediated interactions should be taken into consideration for a comprehensive understanding of ligand binding and activity.

Based on these findings, further modifications will be achieved on this benzimidazole series, either at the level of the benzofused ring or on the lateral chain, where the pyrazole residue will be replaced, among others, with unsaturated nitrogen containing rings to improve the compound’s solubility.

## Figures and Tables

**Table 1:** Inhibition activity of compounds **1**–**12** against CK1δ.

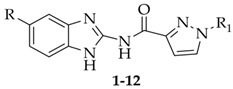			
	R	R	IC_50_ (μM) ^a^
**1**	H	CH_2_COC_6_H_5_	>40
**2**	Me	CH_2_COC_6_H_5_	14.6 ± 3.5
**3**	Cl	CH_2_COC_6_H_5_	1.80 ± 0.50
**4**	H	CH_2_COC_6_H_4-_2-OMe	>40
**5**	Me	CH_2_COC_6_H_4-_2-OMe	>40
**6**	Cl	CH_2_COC_6_H_4-_2-OMe	13.2 ± 4.7
**7**	H	H	>40
**8**	Me	H	>40
**9**	Cl	H	>40
**10**	CF_3_	H	>40
**11**	OMe	H	>40
**12**	NO_2_	H	>40

^a^ Data represent the mean ± SE of three independent experiments performed in duplicate.

**Table 2:** Inhibition activity of compounds **13**–**32** against CK1δ.

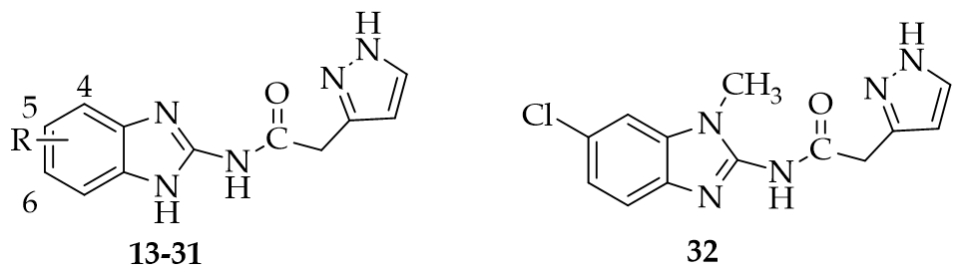		
	R	IC_50_ (μM) ^a^
**13**	H	4.21 ± 1.92
**14**	5-Me	1.64 ± 0.31
**15**	5-Cl	0.485 ± 0.310
**16**	5-CF_3_	1.74 ± 0.32
**17**	5-OMe	6.27 ± 0.95
**18**	5-NO_2_	0.12 ± 0.083
**19**	4-NO_2_	1.22 ± 0.23
**20**	5-terBu	1.00 ± 0.18
**21**	5-OCF_3_	5.99 ± 0.87
**22**	5,6-diCl	0.98 ± 0.35
**23**	5-CN	0.0986 ± 0.0394
**24**	5-CONH_2_	2.53 ± 0.38
**25**	5-SO_2_NH_2_	>40
**2** **6**	5-SO_2_NHMe	10.7 ± 2.3
**27**	5-NHCOMe	>40
**28**	5-NHCOPh	>40
**29**	4-NHCOPh	>40
**30**	5-(1,2,4-triazol-1-yl)	2.59 ± 0.5
**31**	5-(tetrazol-5-yl)	1.54 ± 0.29
**32**	-	20.1 ± 7.3

^a^ Data represent the mean ± SE of three independent experiments performed in duplicate.

## Data Availability

Data are contained within the article and Supplementary Materials.

## Supplementary Materials

The following supporting information can be downloaded at: https://www.mdpi.com/article/10.3390/ph17040468/s1, Video S1: Selected docking poses and relative color-coded per-residue interaction energy fingerprint (electrostatic and hydrophobic) for all the compounds.; Video S2: SuMD binding trajectory of compound **23**; Video S3: Comparison of TTMD unbinding molecular dynamics trajectories of compound **23** and **13**; Figure S1: Comparison between the docking pose of the reference crystallographic ligand IWP-2 (blue) with its relative X-ray conformation (grey) within CK1δ (orange) binding site (PDB ID: 5OKT); Table S1: Physicochemical and pharmacokinetic parameters of derivatives **15**, **18**, **22**, **23**; Scanned ^1^H-NMR spectra of compounds **1**–**32**.

## References

[1] Xu P., Ianes C., Gärtner F., Liu C., Burster T., Bakulev V., Rachidi N., Knippschild U., Bischof J. (2019). Structure, regulation, and (patho-)physiological functions of the stress-induced protein kinase CK1 delta (CSNK1D). Gene.

[2] Lohler J., Hirner H., Schmidt B., Kramer K., Fischer D., Thal D.R., Leithauser F., Knippschild U. (2009). Immunohistochemical characterization of cell-type specific expression of CK1δelta in various tissues of young adult BALB/c mice. PLoS ONE.

[3] Behrend L., Stoter M., Kurth M., Rutter G., Heukeshoven J., Deppert W., Knippschild U. (2000). Interaction of casein kinase 1 delta (CK1δelta) with post-Golgi structures, microtubules, and the spindle apparatus. Eur. J. Cell. Biol..

[4] Cunningham P.S., Ahern S.A., Smith L.C., da Silva Santos C.S., Wager T.T., Bechtold D.A. (2016). Targeting of the circadian clock via CK1δ/ε to improve glucose homeostasis in obesity. Sci. Rep..

[5] Cruciat C.-M. (2014). Casein kinase 1 and Wnt/-catenin signaling. Curr. Opin. Cell. Biol..

[6] Schittek B., Sinnberg T. (2014). Biological functions of casein kinase 1 isoforms and putative roles in tumorigenesis. Mol. Cancer.

[7] Knippschild U., Krüger M., Richter J., Xu P., García-Reyes B., Peifer C., Halekotte J., Bakulev V., Bischof J. (2014). The CK1 family: Contribution to cellular stress response and its role in carcinogenesis. Front. Oncol..

[8] Fulcher L.J., Sapkota G.P. (2020). Functions and regulation of the serine/threonine protein kinase CK1 family: Moving beyond promiscuity. Biochem. J..

[9] Richter J., Rudeck S., Kretz A.L., Kramer K., Just S., Henne-Bruns D., Hillenbrand A., Leithauser F., Lemke J., Knippschild U. (2016). Decreased CK1δ expression predicts prolonged survival in colorectal cancer patients. Tumor Biol..

[10] Eng G.W.L., Edison, Virshup D.M. (2017). Site-specific phosphorylation of casein kinase 1 δ (CK1δ) regulates its activity towards the circadian regulator PER2. PLoS ONE.

[11] Yasojima K., Kuret J., DeMaggio A.J., McGeer E., McGeer P.L. (2000). Casein kinase 1 delta mRNA is upregulated in Alzheimer disease brain. Brain Res..

[12] Li G., Yin H., Kuret J. (2004). Casein kinase 1 delta phosphorylates tau and disrupts its binding to microtubules. J. Biol. Chem..

[13] Martínez-González L., Rodríguez-Cueto C., Cabezudo D., Bartolomé F., Andrés-Benito P., Ferrer I., Gil C., Martín-Requero Á., Fernández-Ruiz J., Martínez A. (2020). Motor neuron preservation and decrease of in vivo TDP-43 phosphorylation by protein CK-1 kinase inhibitor treatment. Sci. Rep..

[14] Gao J., Wang L., Huntley M.L., Perry G., Wang X. (2018). Pathomechanisms of TDP-43 in neurodegeneration. J. Neurochem..

[15] Martinez-Gonzalez L., Cuevas E.P., Tosat-Bitrián C., Nozal V., Gil C., Palomo V., Martín-Requero Á., Martinez A. (2023). TTBK1 and CK1 inhibitors restore TDP-43 pathology and avoid disease propagation in lymphoblast from Alzheimer’s disease patients. Front. Mol. Neurosci..

[16] Alquezar C., Salado I.G., de la Encarnacion A., Perez D.I., Moreno F., Gil C., de Munain A.L., Martinez A., Martin-Requero A. (2016). Targeting TDP-43 phosphorylation by casein kinase-1δ inhibitors: A novel strategy for the treatment of frontotemporal dementia. Mol. Neurodegener..

[17] He S., Wang F., Yung K.K.L., Zhang S., Qu S. (2021). Effects of α-synuclein-associated post-translational modifications in Parkinson’s disease. ACS Chem. Neurosci..

[18] Okochi M., Walter J., Koyama A., Nakajo S., Baba M., Iwatsubo T., Meijer L., Kahle P.J., Haass C. (2000). Constitutive phosphorylation of the Parkinson’s disease-associated alpha-synuclein. J. Biol. Chem..

[19] Jin Y., Li F., Sonoustoun B., Kondru N.C., Yuka A., Martens Y.A., Qiao W., Heckman M.G., Ikezu T.C., Li Z. (2022). APOE4 exacerbates α-synuclein seeding activity and contributes to neurotoxicity in Alzheimer’s disease with Lewy body pathology. Acta Neuropathol..

[20] Paleologou K.E., Oueslati A., Shakked G., Rospigliosi C.C., Kim H.Y., Lamberto G.R., Fernandez C.O., Schmid A., Chegini F., Gai W.P. (2010). Phosphorylation at S87 is enhanced in synucleinopathies, inhibits alpha-synuclein oligomerization, and influences synuclein-membrane interactions. J. Neurosci..

[21] Madsen D.A., Schmidt S.I., Blaabjerg M., Meyer M. (2021). Interaction between parkin and α-synuclein in PARK2-mediated Parkinson’s disease. Cells.

[22] Catarzi D., Varano F., Vigiani E., Lambertucci C., Spinaci A., Volpini R., Colotta V. (2022). Casein kinase 1 delta inhibitors as promising therapeutic agents for neurodegenerative disorders. Curr. Med. Chem..

[23] Varano F., Catarzi D., Calenda S., Vigiani E., Colotta V. (2022). CK1 delta inhibition: An emerging strategy to combat neurodegenerative diseases. Future Med. Chem..

[24] Badura L., Swanson T., Adamowicz W., Adams J., Cianfrogna J., Fisher K., Holland J., Kleiman R., Nelson F., Reynolds L. (2007). An inhibitor of casein kinase Iε induces phase delays in circadian rhythms under free-running and entrained conditions. J. Pharmacol. Exp. Ther..

[25] Adler P., Mayne J., Wlaker K., Ning Z., Figeys D.J. (2019). Therapeutic targeting of casein kinase 1δ/ε in an Alzheimer’s disease mouse model. J. Proteome Res..

[26] Long A., Zhao H., Huang X. (2012). Structural basis for the interaction between casein kinase 1 delta and a potent and selective inhibitor. J. Med. Chem..

[27] Peifer C., Abadleh M., Bischof J., Hauser D., Schattel V., Hirner H., Knippschild U., Laufer S. (2009). 3,4-Diaryl-isoxazoles and -imidazoles as potent dual inhibitors of p38alpha mitogen-activated protein kinase and casein kinase 1delta. J. Med. Chem..

[28] Halekotte J., Witt L., Ianes C., Krüger M., Bührmann M., Rauh D., Pichlo C., Brunstein E., Luxenburger A., Baumann U. (2017). Optimized 4,5-diarylimidazoles as potent/selective inhibitors of protein kinase CK1δ and their structural relation to p38α MAPK. Molecules.

[29] Bibian M., Rahaim R.J., Choi J.Y., Noguchi Y., Schürer S., Chen W., Nakanishi S., Licht K., Rosenberg L.H., Li L. (2013). Development of highly selective casein kinase 1/1 (CK1) inhibitors with potent antiproliferative properties. Bioorg. Med. Chem. Lett..

[30] Bischof J., Leban J., Zaja M., Grothey A., Radunsky B., Othersen O., Strobl S., Vitt D., Knippschild U. (2012). 2-Benzamido-N-(1H-benzo[d]imidazol-2-yl)thiazole-4-carboxamide derivatives as potent inhibitors of CK1δelta/epsilon. Amino Acids.

[31] Richter J., Bischof J., Zaja M., Kohlhof H., Othersen O., Vitt D., Alscher V., Pospiech I., García-Reyes B., Berg S. (2014). Difluoro-dioxolo-benzoimidazol-benzamides as potent inhibitors of CK1δ and ε with nanomolar inhibitory activity on cancer cell proliferation. J. Med. Chem..

[32] Wright S.W., Arnold E.P., Yang X. (2018). Steric redirection of alkylation in 1H-pyrazole-3-carboxylate esters. Tetrahedron Lett..

[33] Jones R.G., Mann M.J. (1953). New methods of synthesis of S-aminoethylpyrazoles. J. Am. Chem. Soc..

[34] Manuel L.R., Porter C.C., Kueh F.A., Jr Jensen N.P., Schmitt S.M., Windholz T.B., Beattie T.R., Carty J.A., Christensen B.G., Shen T.Y. (1970). Inhibition of adrenal phenethanolamine /V-methyltransferase by substituted benzimidazoles. J. Med. Chem..

[35] Bella M., Milata V., Larina L.I. (2011). 2-Amino-X-nitrobenzimidazoles as precursors of food-borne carcinogens: A new approach to IQ synthesis. J. Het. Chem..

[36] Frei R., Breitbach A.S., Blackwell H.E. (2012). 2-Aminobenzimidazole derivatives strongly inhibit and disperse pseudomonas aeruginosa biofilms. Angew. Chem. Int. Ed..

[37] Liu W., Lau F., Liu K., Wood H.B., Zhou G., Chen Y., Li Y., Akiyama T.E., Castriota G., Einstein M. (2011). Benzimidazolones: A new class of selective peroxisome proliferator-activated receptor γ (PPARγ) modulators. J. Med. Chem..

[38] Arienti K.L., Brunmark A., Axe F.U., McClure K., Lee A., Blevitt J., Neff D.K., Huang L., Crawford S., Pandit C.R. (2005). Checkpoint kinase inhibitors: SAR and radioprotective properties of a series of 2-arylbenzimidazoles. J. Med. Chem..

[39] Püsküll M.O., Yıldız S., Göker H. (2010). Synthesis and antistaphylococcal activity of N-substituted-1H-benzimidazole-sulphonamides. Arch. Pharm. Chem. Life Sci..

[40] Hofmans S., Vanden Berghe T., Devisscher L., Hassannia B., Lyssens S., Joossens J., Van Der Veken P., Vandenabeele P., Augustyns K. (2016). Novel ferroptosis inhibitors with improved potency and ADME properties. J. Med. Chem..

[41] Kikuchi K., Hannak R.B., Guo M.-J., Kirbyd A.J., Hilvert D. (2006). Toward bifunctional antibody catalysis. Bioorg. Med. Chem..

[42] Huigens III R.W., Reyes S., Reed C.S., Bunders C., Rogers S.A., Steinhauer A.T., Melander C. (2010). The chemical synthesis and antibiotic activity of a diverse library of 2-aminobenzimidazole small molecules against MRSA and multidrug-resistant *A. baumannii*. Bioorg. Med. Chem..

[43] Güngór T., Fouquet A., Teuton J.-M., Provost D., Gazes M., Cloarec A. (1992). Cardiotonic agents. Synthesis and cardiovascular properties of novel 2-arylbenzimidazoles and azabenzimidazole. J. Med. Chem..

[44] Duffin G.R., Lyddiatt A., Dolan V.J., Angus K.L. (2012). Benzimidazole Compounds and Their Use as Chromatographic Ligands.

[45] Voskresensky S., Makosza M. (2000). Selective one-pot N-monomethylation of 2-nitroanilines under Ptc conditions. Synth. Commun..

[46] Berman H.M., Westbrook J., Feng Z., Gilliland G., Bhat T.N., Weissig H., Shindyalov I.N., Bourne P.E. (2000). The Protein Data Bank. Nucleic Acids Res..

[47] Sciabola S., Benedetti P., D’Arrigo G., Torella R., Baroni M., Cruciani G., Spyrakis F. (2019). Discovering new casein kinase 1δ inhibitors with an innovative molecular dynamics enabled virtual screening workflow. ACS Med. Chem. Lett..

[48] Karthikeyan C., Jharia P., Waiker D.K., Nusbaum A.C., Amawi H., Kirwen E.M., Christman R., Arudra S.K.C., Meijer L., Tiwari A.K. (2017). N-(1H-Pyrazol-3-Yl)quinazolin-4-amines as a novel class of casein kinase 1δ/ε inhibitors: Synthesis, biological evaluation and molecular modeling studies. Bioorg. Med. Chem. Lett..

[49] Cozza G., Gianoncelli A., Montopoli M., Caparrotta L., Venerando A., Meggio F., Pinna L.A., Zagotto G., Moro S. (2008). Identification of novel protein kinase CK1 delta (CK1delta) inhibitors through structure-based virtual screening. Bioorg Med. Chem. Lett..

[50] Grieco I., Bissaro M., Tiz D.B., Perez D.I., Perez C., Martinez A., Redenti S., Mariotto E., Bortolozzi R., Viola G. (2021). Developing novel classes of protein kinase CK1δ inhibitors by fusing [1,2,4] triazole with different bicyclic heteroaromatic systems. Eur. J. Med. Chem..

[51] Sabbadin D., Salmaso V., Sturlese M., Moro S. (2018). Supervised molecular dynamics (SuMD) approaches in drug design. Methods Mol. Biol..

[52] Menin S., Pavan M., Salmaso V., Sturlese M., Moro S. (2023). Thermal titration molecular dynamics (TTMD): Not your usual post-docking refinement. Int. J. Mol. Sci..

[53] Pavan M., Menin S., Bassani D., Sturlese M., Moro S. (2022). Qualitative estimation of protein-ligand complex stability through thermal titration molecular dynamics simulations. J. Chem. Inf. Model..

[54] Salado I.G., Redondo M., Bello M.L., Perez C., Liachko N.F., Kraemer B.C., Miguel L., Lecourtois M., Gil C., Martinez A. (2014). Protein kinase CK-1 inhibitors as new potential drugs for amyotrophic lateral sclerosis. J. Med. Chem..

[55] Molecular Operating Environment (MOE)|MOEsaic|PSILO. https://www.chemcomp.com/Products.htm.

[56] Korb O., Stützle T., Exner T.E. (2006). PLANTS: Application of Ant Colony Optimization to Structure-Based Drug Design. Lect. Notes Comput. Sci..

[57] Stewart J.J.P. (2007). Optimization of parameters for semiempirical methods V: Modification of NDDO approximations and application to 70 elements. J. Mol. Model..

[58] Molecular Modeling Software|OpenEye Scientific. https://www.eyesopen.com/.

[59] Tautomers—Applications. https://docs.eyesopen.com/applications/quacpac/tautomers/tautomers.html.

[60] OMEGA 4.2.2.0—Applications. https://docs.eyesopen.com/applications/omega/index.html.

[61] FixpKa—Applications. https://docs.eyesopen.com/applications/quacpac/fixpka/fixpka.html.

[62] MolCharge—Applications. https://docs.eyesopen.com/applications/quacpac/molcharge/molcharge.html.

[63] Seidel T., Schuetz D.A., Garon A., Langer T. (2019). The Pharmacophore Concept and Its Applications in Computer-Aided Drug Design. Prog. Chem. Org. Nat. Prod..

[64] MPlayer—The Movie Player. http://www.mplayerhq.hu/design7/news.html.

[65] Gnuplot Homepage. http://www.gnuplot.info/index.html.

[66] RDKit. http://www.rdkit.org/.

[67] Pettersen E.F., Goddard T.D., Huang C.C., Couch G.S., Greenblatt D.M., Meng E.C., Ferrin T.E. (2004). UCSF Chimera—A visualization system for exploratory research and analysis. J. Comput. Chem..

[68] Humphrey W., Dalke A., Schulten K. (1996). VMD: Visual molecular dynamics. J. Mol. Graph..

[69] Case D.A., Cheatham T.E., Darden T., Gohlke H., Luo R., Merz K.M., Onufriev A., Simmerling C., Wang B., Woods R.J. (2005). The Amber biomolecular simulation programs. J. Comput. Chem..

[70] Case D.A., Walker R.C., Cheatham T.E., Simmerling C., Roitberg A., Merz K.M., Luo R., Li P., Darden T., Sagui C. Amber 2022 Reference Manual. https://ambermd.org/doc12/Amber22.pdf.

[71] Maier J.A., Martinez C., Kasavajhala K., Wickstrom L., Hauser K.E., Simmerling C. (2015). Ff14SB: Improving the accuracy of protein side chain and backbone parameters from Ff99SB. J. Chem. Theory Comput..

[72] Wang J., Wolf R.M., Caldwell J.W., Kollman P.A., Case D.A. (2004). Development and Testing of a General Amber Force Field. J. Comput. Chem..

[73] Jakalian A., Jack D.B., Bayly C.I. (2002). Fast, Efficient Generation of High-Quality Atomic Charges. AM1-BCC Model: II. Parameterization and Validation. J. Comput. Chem..

[74] Jorgensen W.L., Chandrasekhar J., Madura J.D., Impey R.W., Klein M.L. (1983). Comparison of simple potential functions for simulating liquid water. J. Chem. Phys..

[75] Davidchack R.L., Handel R., Tretyakov M.V. (2009). Langevin thermostat for rigid body dynamics. J. Chem. Phys..

[76] Kräutler V., Van Gunsteren W.F., Hünenberger P.H. (2001). A fast SHAKE algorithm to solve distance constraint equations for small molecules in molecular dynamics simulations. J. Comput. Chem..

[77] Kholmurodov K., Smith W., Yasuoka K., Darden T., Ebisuzaki T.A. (2000). Smooth-particle mesh Ewald Method for DL_POLY Molecular Dynamics Simulation Package on the Fujitsu VPP700. J. Comput. Chem..

[78] Faller R., De Pablo J.J. (2002). Constant pressure hybrid molecular dynamics–Monte Carlo simulations. J. Chem. Phys..

[79] Eastman P., Swails J., Chodera J.D., McGibbon R.T., Zhao Y., Beauchamp K.A., Wang L.P., Simmonett A.C., Harrigan M.P., Stern C.D. (2017). OpenMM 7: Rapid development of high performance algorithms for molecular dynamics. PLoS Comput. Biol..

[80] Phillips J.C., Hardy D.J., Maia J.D.C., Stone J.E., Ribeiro J.V., Bernardi R.C., Buch R., Fiorin G., Hénin J., Jiang W. (2020). Scalable molecular dynamics on CPU and GPU architectures with NAMD. J. Chem. Phys..

[81] Gowers R.J., Linke M., Barnoud J., Reddy T.J.E., Melo M.N., Seyler S.L., Domanski J., Dotson D.L., Buchoux S., Kenney I.M. MDAnalysis: A Python Package for the Rapid Analysis of Molecular Dynamics Simulations. Proceedings of the 15th Python in Science Conference.

[82] Michaud-Agrawal N., Denning E.J., Woolf T.B., Beckstein O. (2011). MDAnalysis: A Toolkit for the analysis of molecular dynamics simulations. J. Comput. Chem..

[83] Hunter J.D. (2007). Matplotlib: A 2D Graphics Environment. Comput. Sci. Eng..

[84] Pavan M., Menin S., Bassani D., Sturlese M., Moro S. (2022). Implementing a scoring function based on interaction fingerprint for Autogrow4: Protein Kinase CK1δ as a case study. Front. Mol. Biosci..

[85] Wójcikowski M., Zielenkiewicz P., Siedlecki P. (2015). Open Drug Discovery Toolkit (ODDT): A New Open-Source Player in the Drug Discovery Field. J. Cheminform..

[86] (2021). Schrödinger Release 2021-1: QikProp.

