# Supplementary material for: Structural Investigations on 2-Amidobenzimidazole Derivatives as New Inhibitors of Protein Kinase CK1 Delta
Source: Pharmaceuticals (Basel). 2024 Apr 7;17(4):468. doi: 10.3390/ph17040468 (PMC11054282; doi:10.3390/ph17040468)
Supplement: Supplementary file 1 [file pharmaceuticals-17-00468-s001.zip › Supplementary_Materials.pdf]

Supplementary Materials

Structural investigations on 2-amidobenzimidazole derivatives as new inhibitors of protein kinase CK1 delta

Sara Calenda, Daniela Catarzi, Flavia Varano, Erica Vigiani, Rosaria Volpini, Catia Lambertucci, Andrea Spinaci, Letizia Trevisan, Ilenia Grieco, Stephanie Federico, Giampiero Spalluto, Gianluca Novello, Veronica Salmaso, Stefano Moro, Vittoria Colotta

Table of Contents

- **Figure S1**. Comparison between the docking pose of the reference crystallographic ligand IWP-2 (blue) with its relative X-ray conformation (grey) within CK1δ (orange) binding site (PDB ID: 5OKT)………………………… *Page S1*
- **Table S1.** Physicochemical and pharmacokinetic parameters of derivatives **15**, **18**, **22**, **23**………………………… *Page S2*
- **Scanned ^{1}H-NMR spectra of derivatives 1-32**………………………… *Page S3*

Figure S1

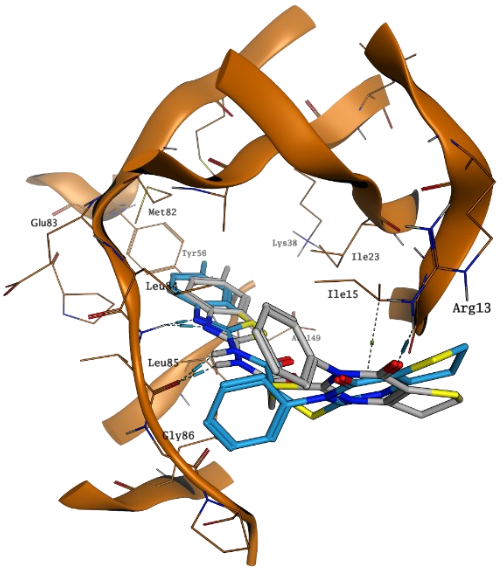

Figure S1. Comparison between the docking pose of the reference crystallographic ligand IWP-2 (blue) with its relative X-ray conformation (grey) within CK1δ (orange) binding site (PDB ID: 5OKT).

Table S1. *Physicochemical and pharmacokinetic parameters of derivatives* ***15, 18, 22, 23***

Compound ID	**15**	**18**	**22**	**23**	**PF-670462***
logP (o/w)	2.814	1.557	3.160	1.573	3.882
logS	-4.421	-3.776	-4.989	-4.631	-5.839
logHERG	-5.369	-5.304	-5.198	-5.507	-5.324
Caco-2	376.9	43.2	319.4	81.8	753.7
logBB	-0.869	-2.058	-0.812	-1.798	-0.503
logKHSA	0.200	0.027	0.300	-0.075	0.687
HOA (%)	89 %	65 %	90 %	70 %	100

** Positive control used in the CK1δ Activity Assays.*

Legenda

- Good (green)
- Average (yellow)
- Bad (red)

	**Range or recommended values**
logP (o/w): Predicted octanol/water partition coefficient.	−2.0 – 6.5
logS: Predicted aqueous solubility (mol dm^{-3}).	−6.5 – 0.5
logHERG: Predicted IC_{50} value for blockage of HERG K+ channels.	−5
Caco-2: Predicted apparent Caco-2 cell permeability in nm/sec.	<25 poor, >500 great
logBB: Predicted brain/blood partition coefficient.	−3.0 – 1.2
logKHSA: Prediction of binding to human serum albumin.	−1.5 – 1.5
HOA (%): Prediction of human oral absorption on 0 to 100% scale.	>80% is high, <25% is poor

^{1}H NMR scanned spectra (DMSOd6) of derivatives 1-32.

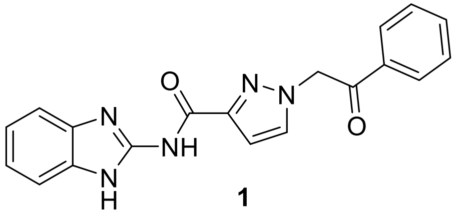
N
NH
O
N
N
H
O

1

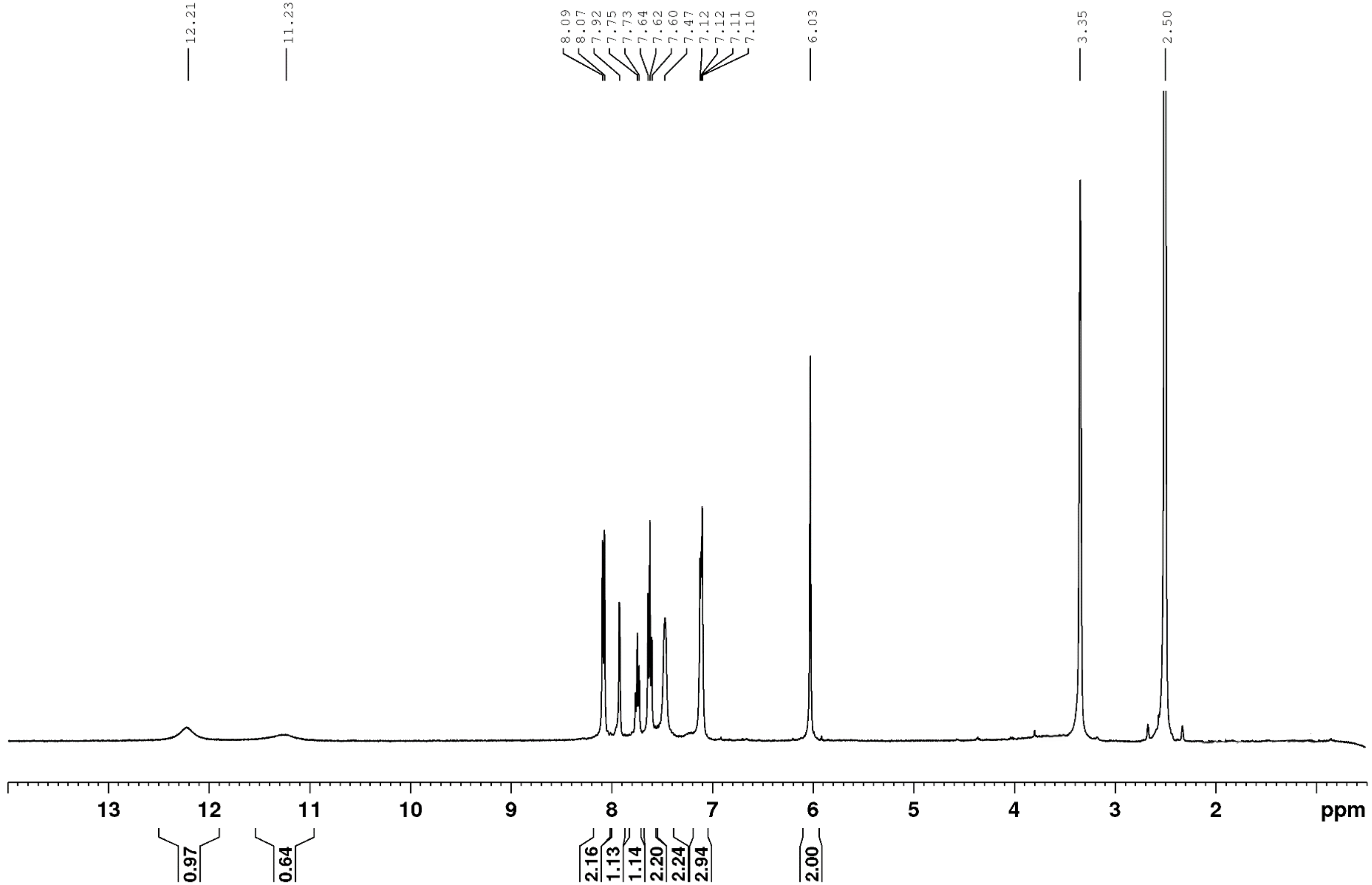
12.21
11.23
8.09
8.07
7.92
7.75
7.73
7.64
7.62
7.60
7.47
7.12
7.12
7.11
7.10
6.03
3.35
2.50
13 12 11 10 9 8 7 6 5 4 3 2 ppm
0.97
0.64
2.16
1.13
1.14
2.20
2.24
2.94
2.00

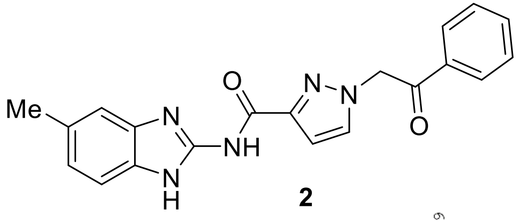
Me
N
NH
N
H
O
N
N
O

2

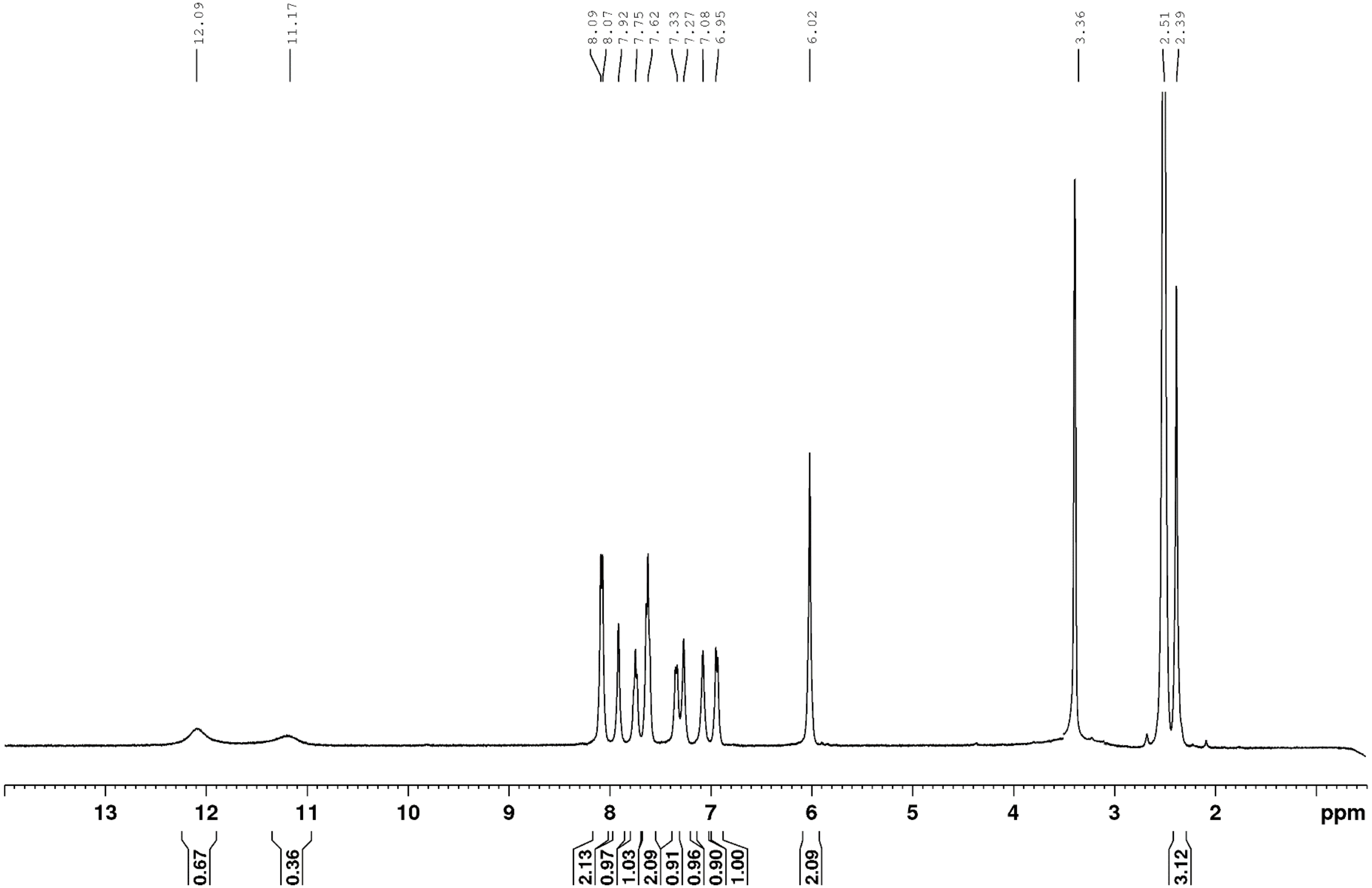
12.09
11.17
8.09
8.07
7.92
7.75
7.62
7.33
7.27
7.08
6.95
6.02
3.36
2.51
2.39
13
12
11
10
9
8
7
6
5
4
3
2
ppm
0.67
0.36
2.13
0.97
1.03
2.09
0.91
0.96
0.90
1.00
2.09
3.12

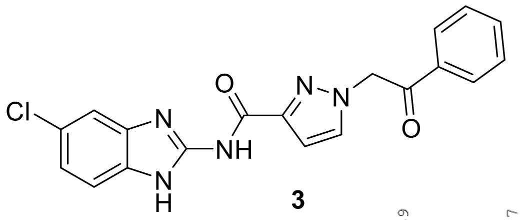
Cl
N
NH
N
H
O
N
N
O

3

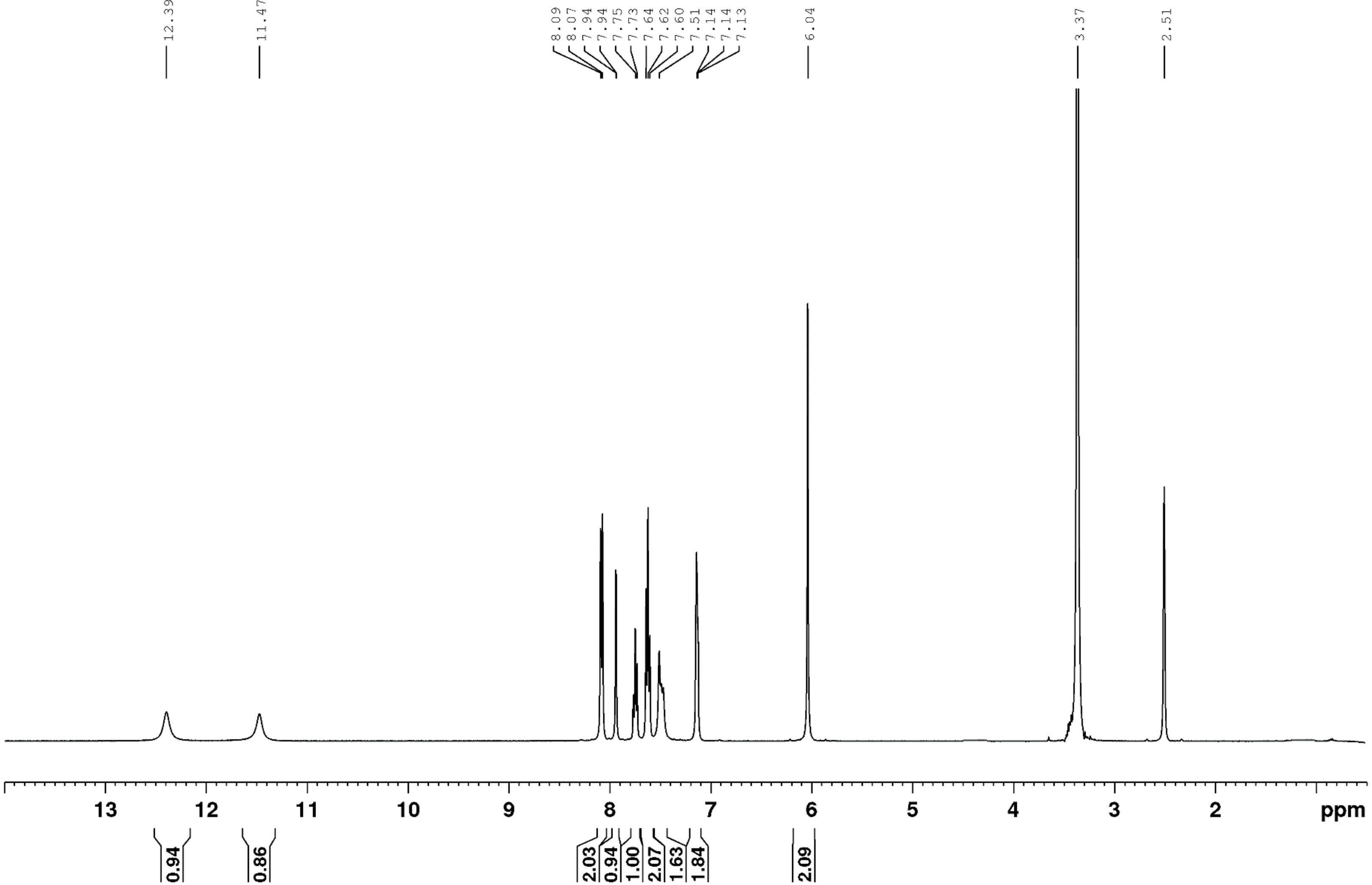
12.39
11.47
8.09
8.07
7.94
7.94
7.75
7.73
7.64
7.62
7.60
7.51
7.14
7.14
7.13
6.04
3.37
2.51
13
12
11
10
9
8
7
6
5
4
3
2
ppm
0.94
0.86
2.03
0.94
1.00
2.07
1.63
1.84
2.09

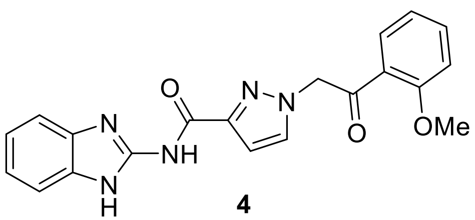

4

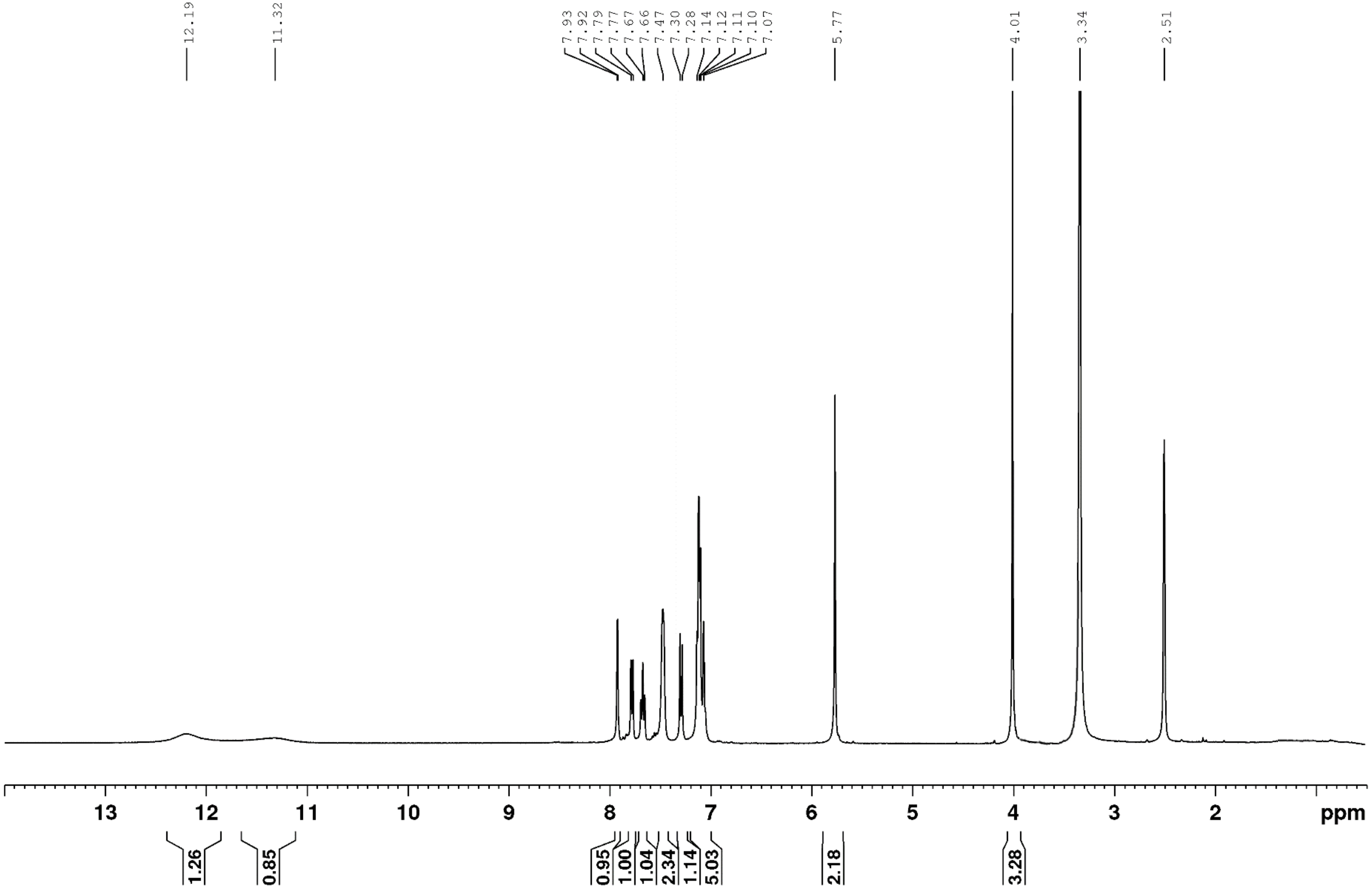
12.19
11.32
7.93
7.92
7.79
7.77
7.67
7.66
7.47
7.30
7.28
7.14
7.12
7.11
7.10
7.07
5.77
4.01
3.34
2.51
13
12
11
10
9
8
7
6
5
4
3
2
ppm
1.26
0.85
0.95
1.00
1.04
2.34
1.14
5.03
2.18
3.28

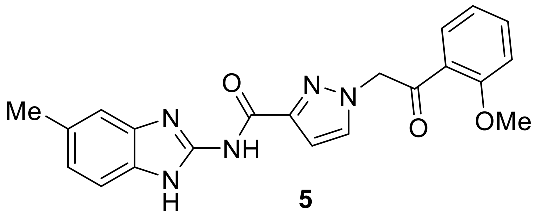
Me
N
NH
N
H
O
N
N
O
OMe

5

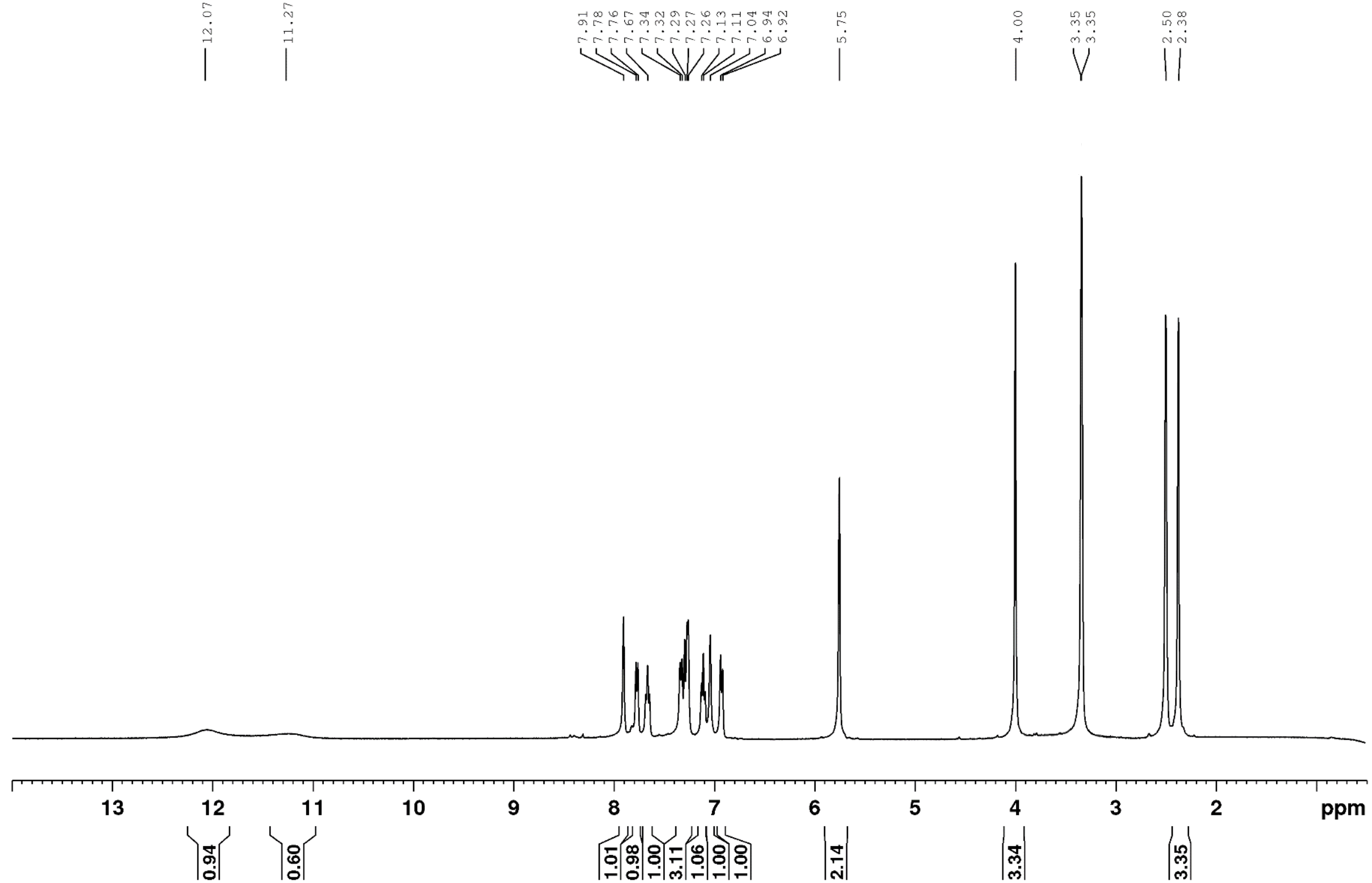
12.07
11.27
7.91
7.78
7.76
7.67
7.34
7.32
7.29
7.27
7.26
7.13
7.11
7.04
6.94
6.92
5.75
4.00
3.35
3.35
2.50
2.38
13
12
11
10
9
8
7
6
5
4
3
2
ppm
0.94
0.60
1.01
0.98
1.00
3.11
1.06
1.00
1.00
2.14
3.34
3.35

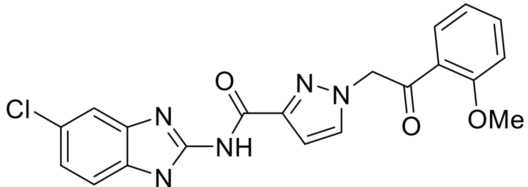

Cl
N
NH
N
H
O
N
N
O
OMe

6

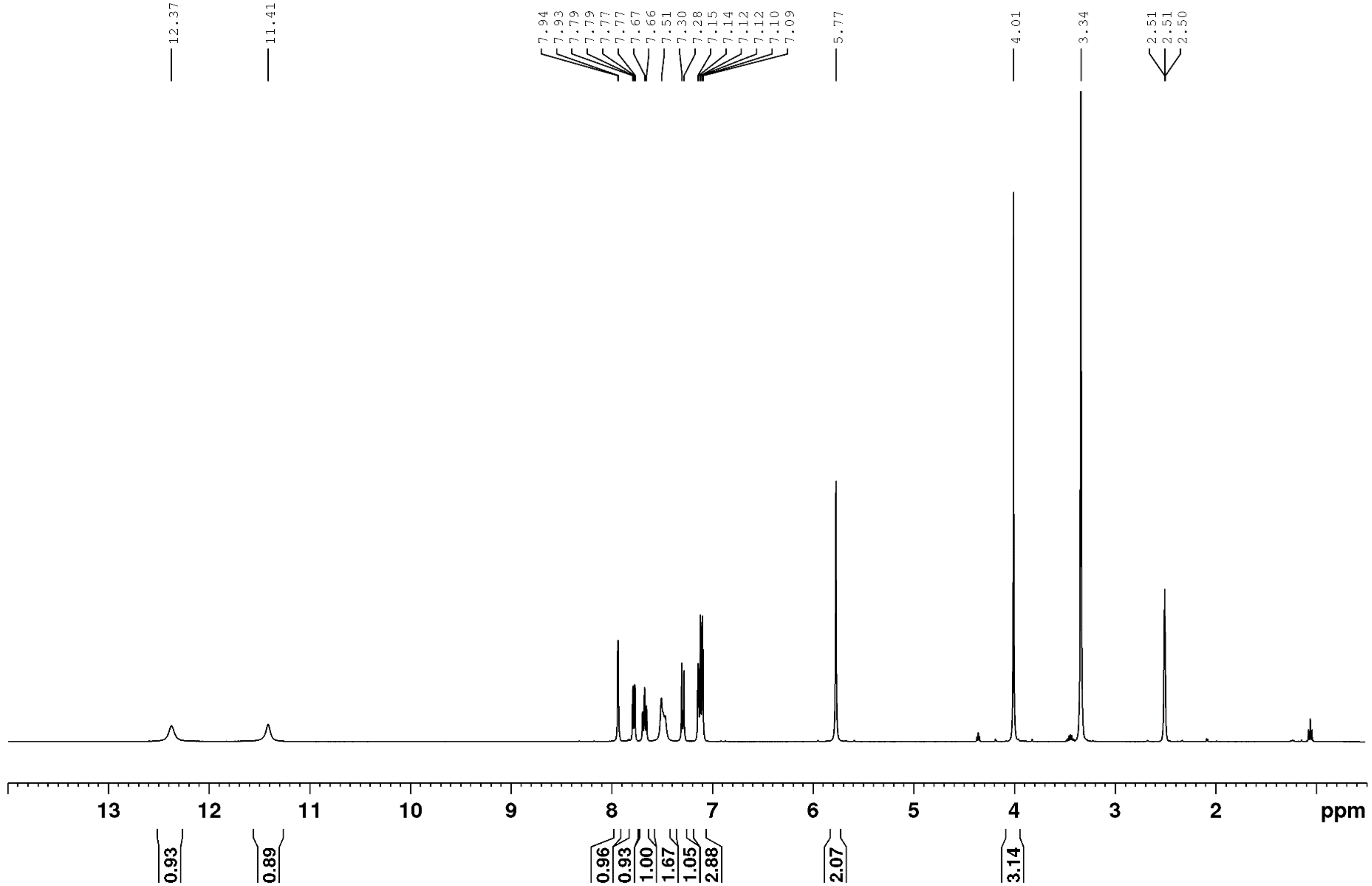

12.37
11.41
7.94
7.93
7.79
7.79
7.77
7.77
7.67
7.66
7.51
7.30
7.28
7.15
7.14
7.12
7.12
7.10
7.09
5.77
4.01
3.34
2.51
2.51
2.50
13
12
11
10
9
8
7
6
5
4
3
2
ppm
0.93
0.89
0.96
0.93
1.00
1.67
1.05
2.88
2.07
3.14

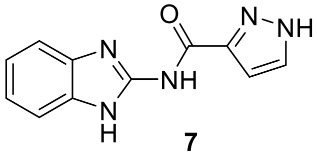
O
N
NH
N
NH
N
H

7

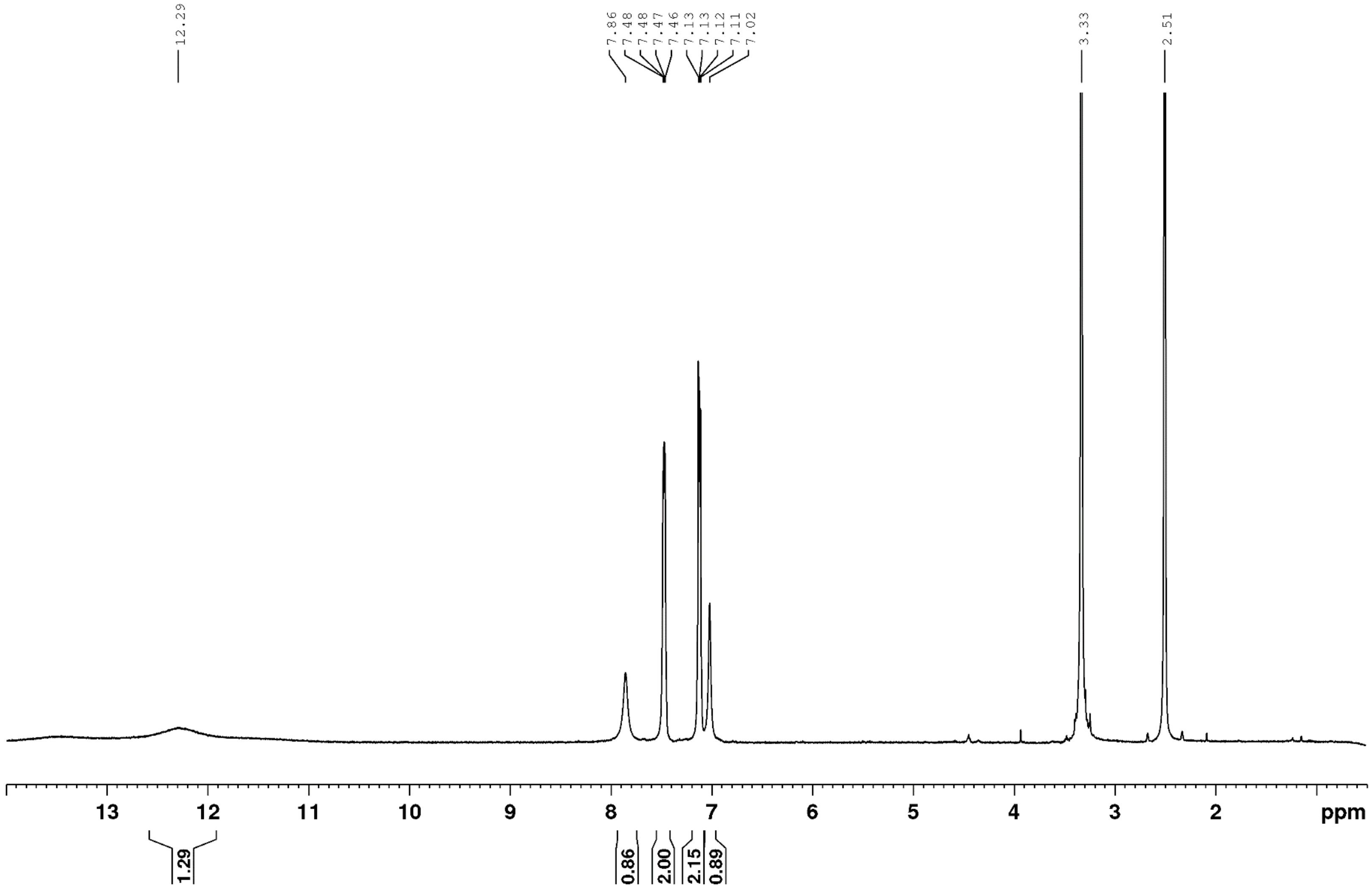
12.29
7.86
7.48
7.48
7.47
7.46
7.13
7.13
7.12
7.11
7.02
3.33
2.51
13
12
11
10
9
8
7
6
5
4
3
2
ppm
1.29
0.86
2.00
2.15
0.89

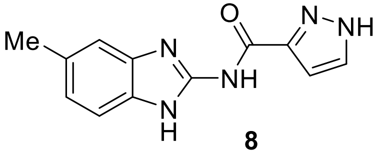
Me
N
NH
N
H
O
N
NH

8

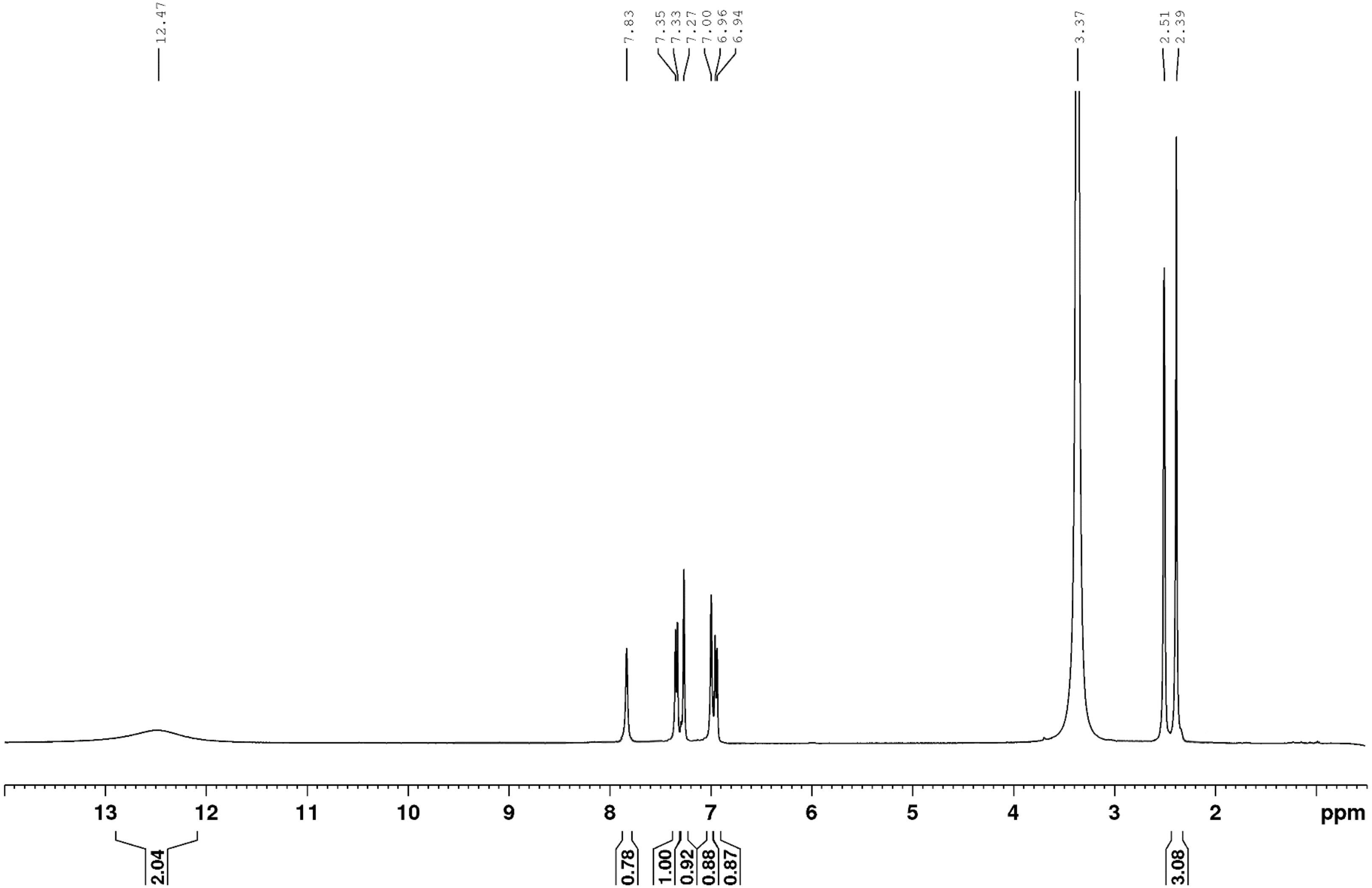
12.47
7.83
7.35
7.33
7.27
7.00
6.96
6.94
3.37
2.51
2.39
13 12 11 10 9 8 7 6 5 4 3 2 ppm
2.04
0.78
1.00
0.92
0.88
0.87
3.08

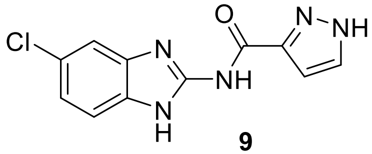
Cl
N
NH
N
H
O
N
NH

9

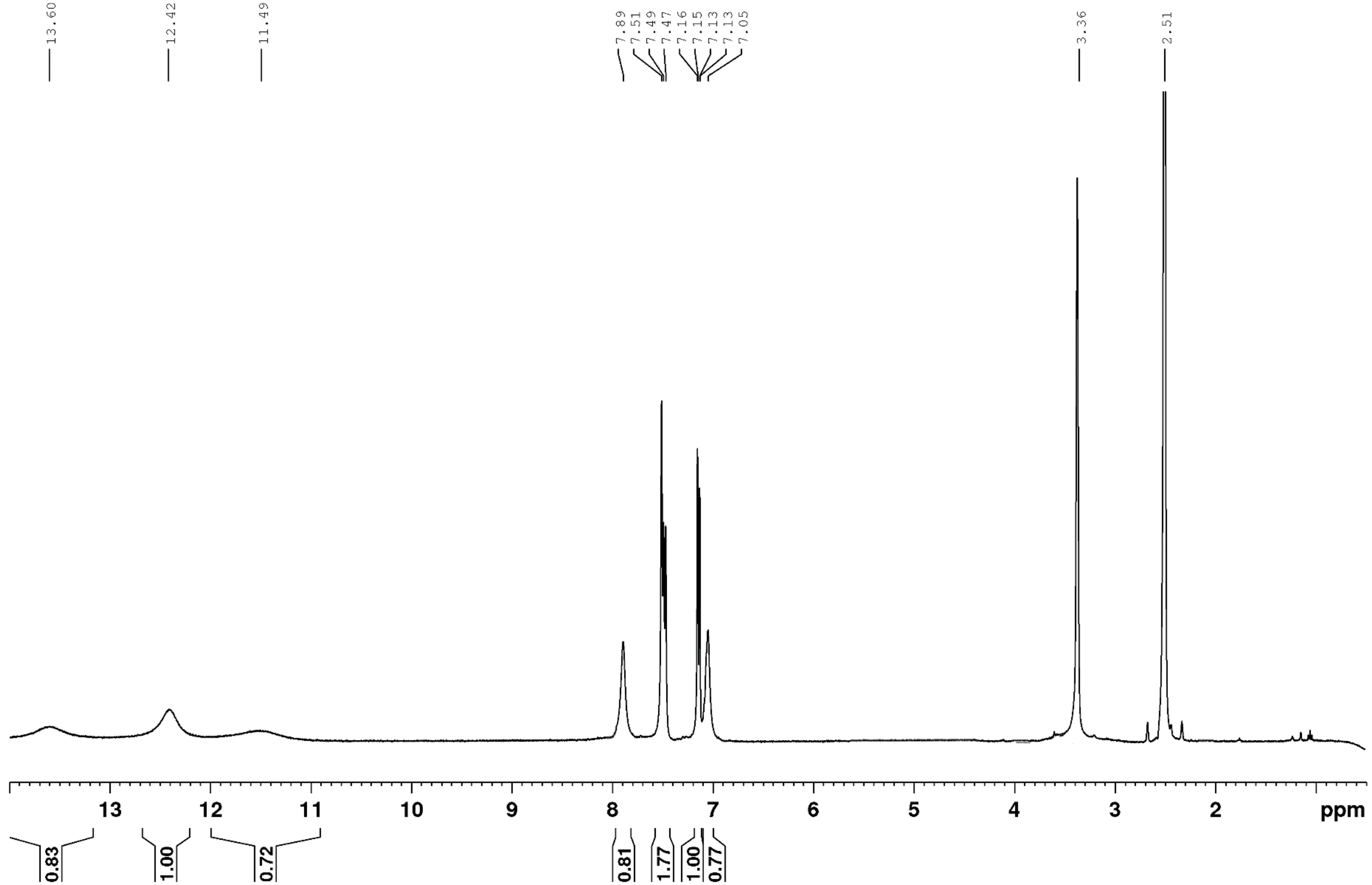
13.60
12.42
11.49
7.89
7.51
7.49
7.47
7.16
7.15
7.13
7.13
7.05
3.36
2.51
13
12
11
10
9
8
7
6
5
4
3
2
ppm
0.83
1.00
0.72
0.81
1.77
1.00
0.77

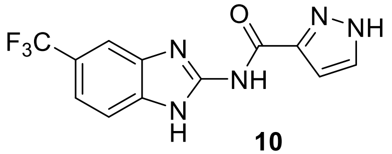
F3C
N
O
N
NH
NH
N
H

10

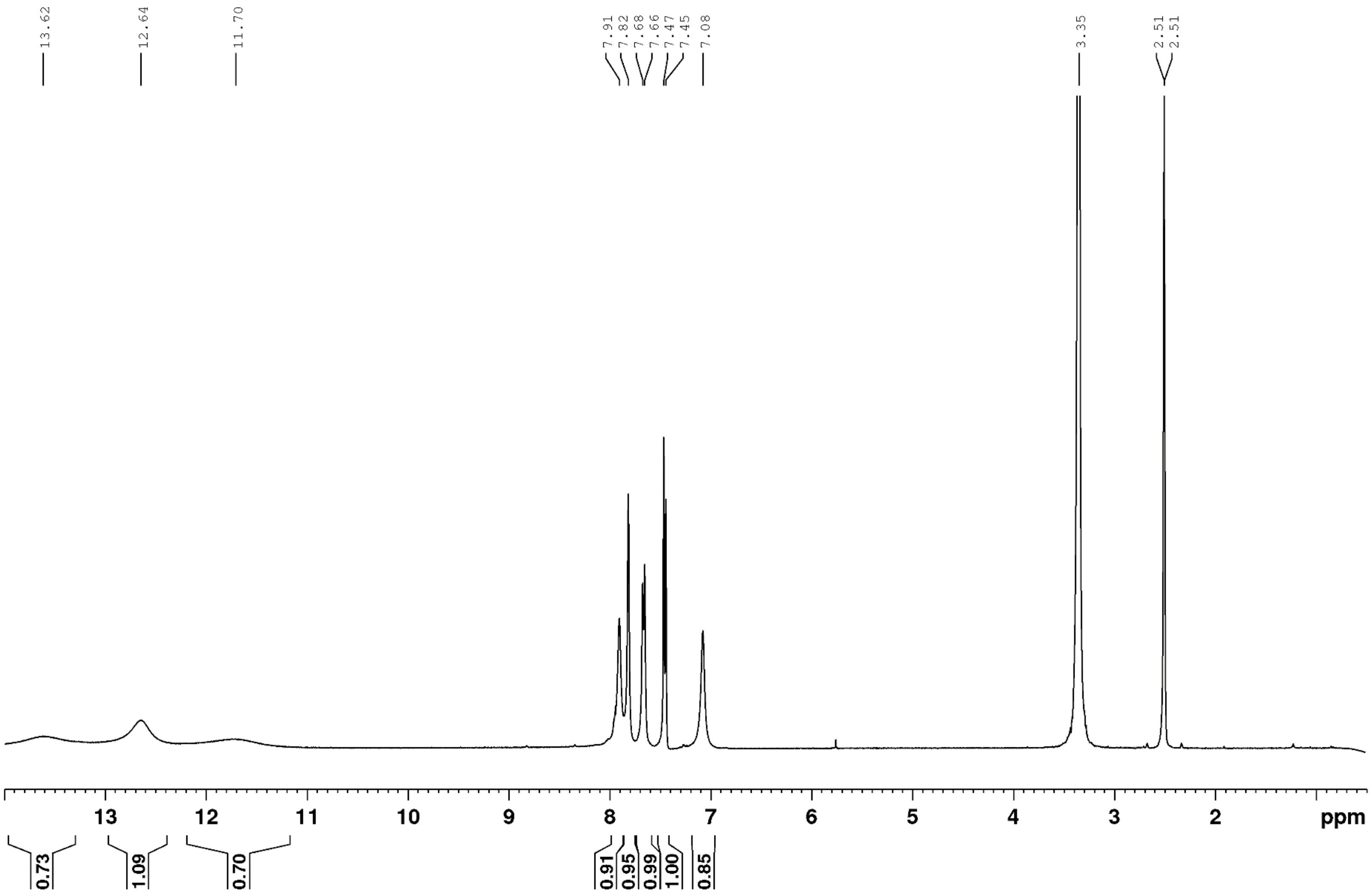
13.62
12.64
11.70
7.91
7.82
7.68
7.66
7.47
7.45
7.08
3.35
2.51
2.51
13
12
11
10
9
8
7
6
5
4
3
2
ppm
0.73
1.09
0.70
0.91
0.95
0.99
1.00
0.85

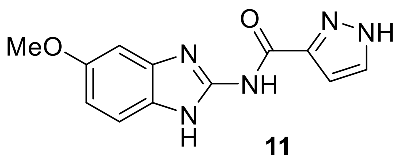

11

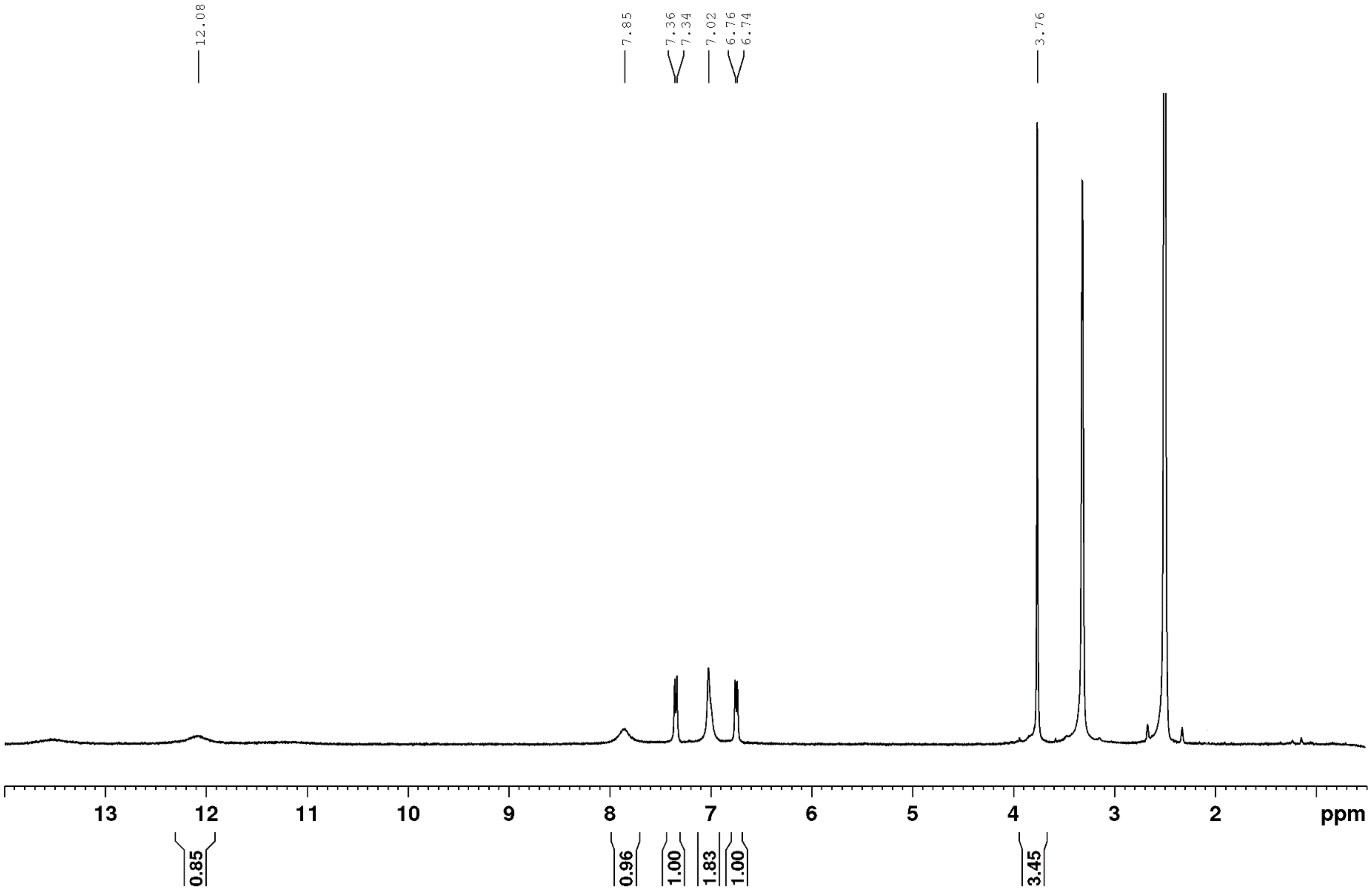
12.08
7.85
7.36
7.34
7.02
6.76
6.74
3.76
13
12
11
10
9
8
7
6
5
4
3
2
ppm
0.85
0.96
1.00
1.83
1.00
3.45

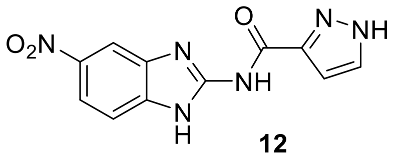

O2N
N
NH
N
H
O
N
NH

12

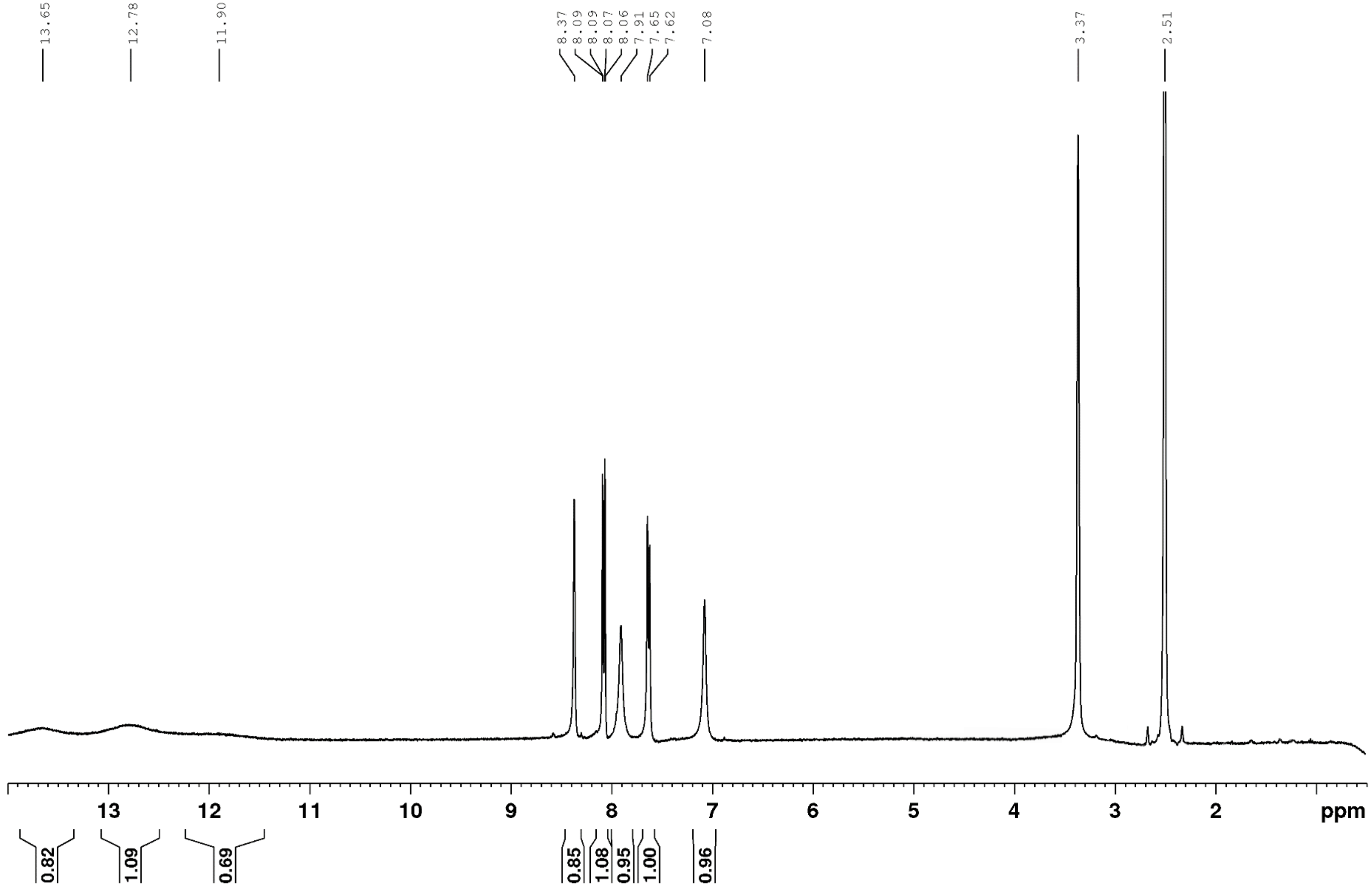

13.65
12.78
11.90
8.37
8.09
8.09
8.07
8.06
7.91
7.65
7.62
7.08
3.37
2.51
13
12
11
10
9
8
7
6
5
4
3
2
ppm
0.82
1.09
0.69
0.85
1.08
0.95
1.00
0.96

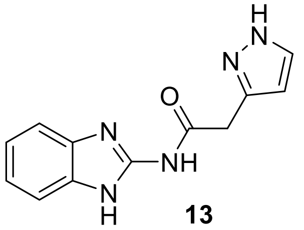
N
NH
N
H
O
N
N
H

13

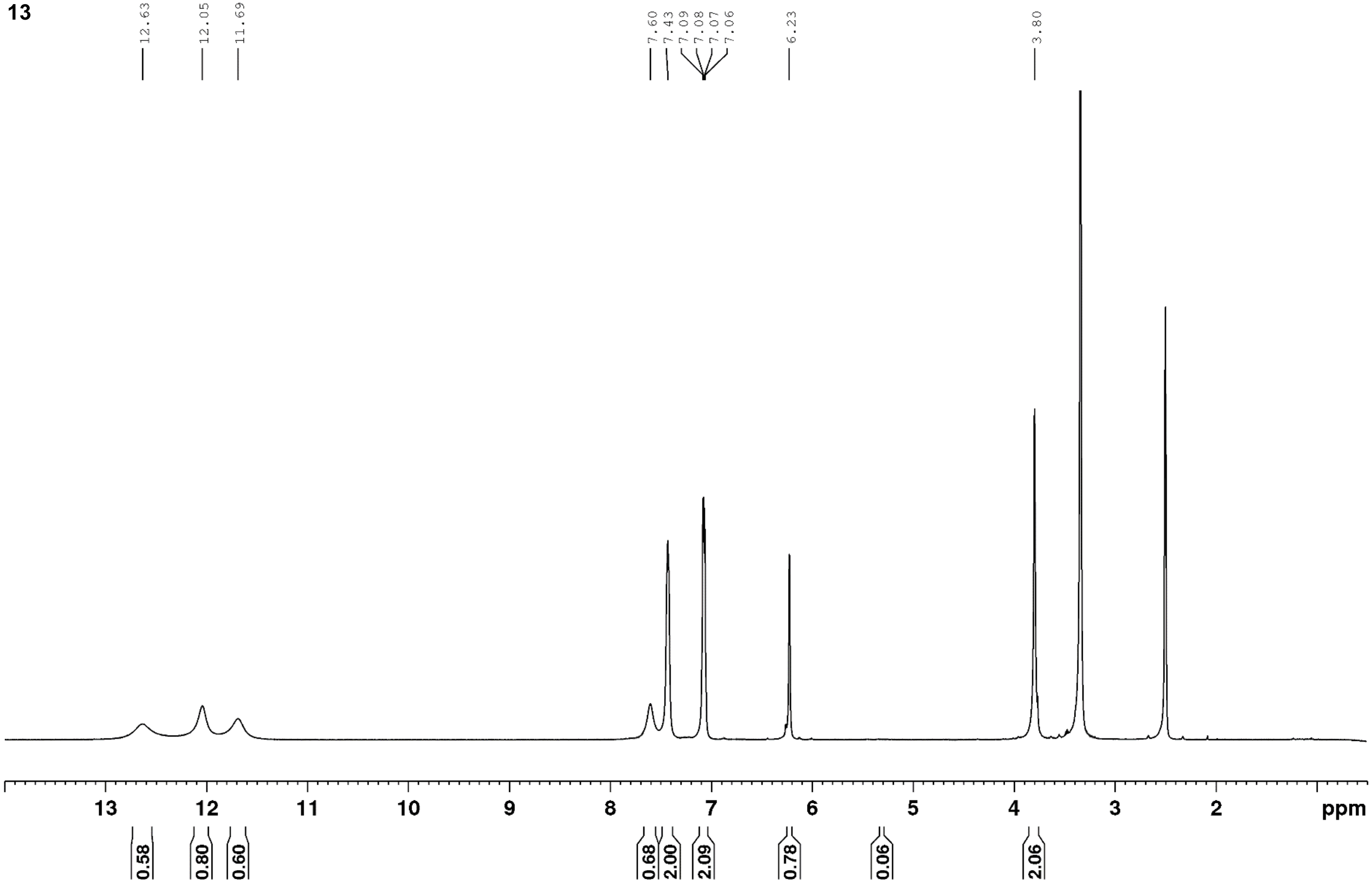
12.63
12.05
11.69
7.60
7.43
7.09
7.08
7.07
7.06
6.23
3.80
13
12
11
10
9
8
7
6
5
4
3
2
ppm
0.58
0.80
0.60
0.68
2.00
2.09
0.78
0.06
2.06

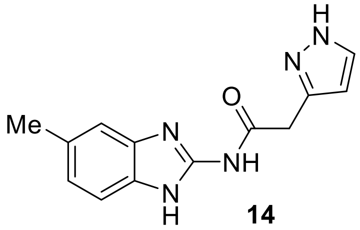

Me
N
NH
H
O
N
H
N

14

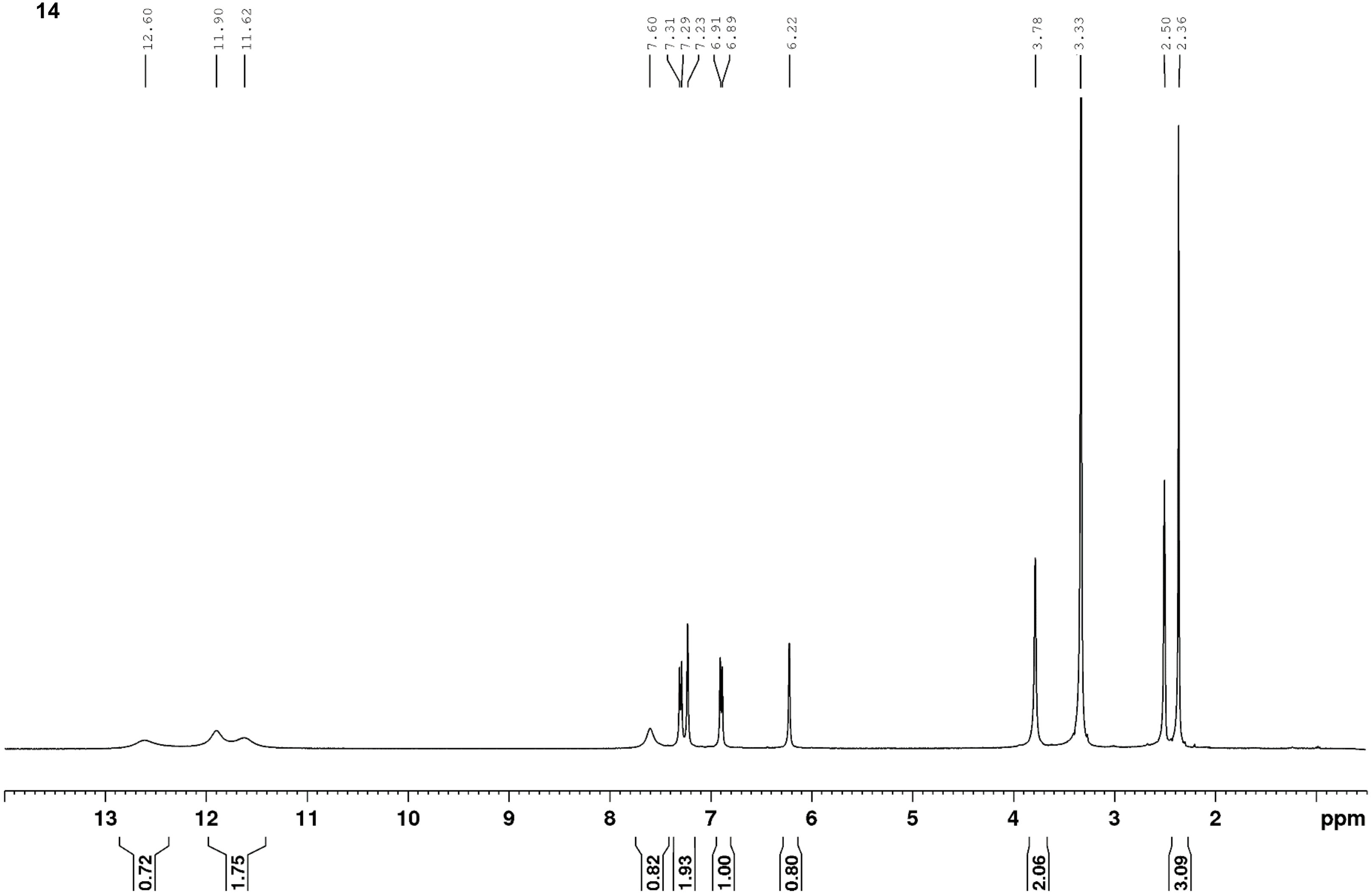

12.60
11.90
11.62
7.60
7.31
7.29
7.23
6.91
6.89
6.22
3.78
3.33
2.50
2.36
13
12
11
10
9
8
7
6
5
4
3
2
ppm
0.72
1.75
0.82
1.93
1.00
0.80
2.06
3.09

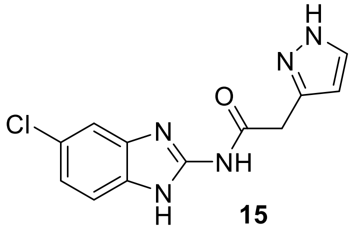

15

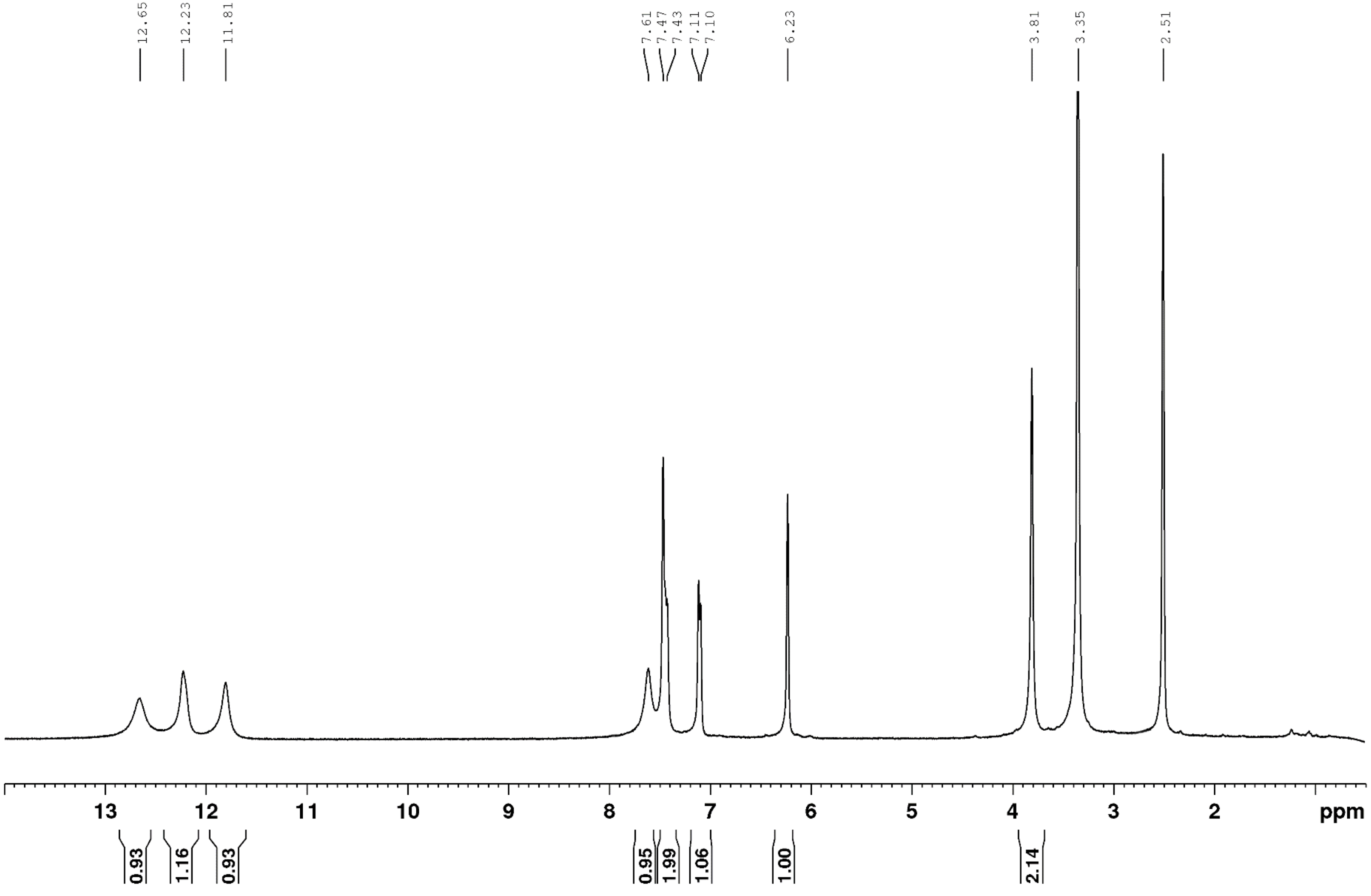

12.65
12.23
11.81
7.61
7.47
7.43
7.11
7.10
6.23
3.81
3.35
2.51
13
12
11
10
9
8
7
6
5
4
3
2
ppm
0.93
1.16
0.93
0.95
1.99
1.06
1.00
2.14

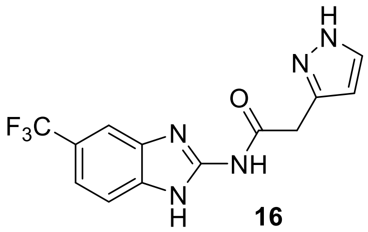
F3C
N
NH
O
N
N
H
H

16

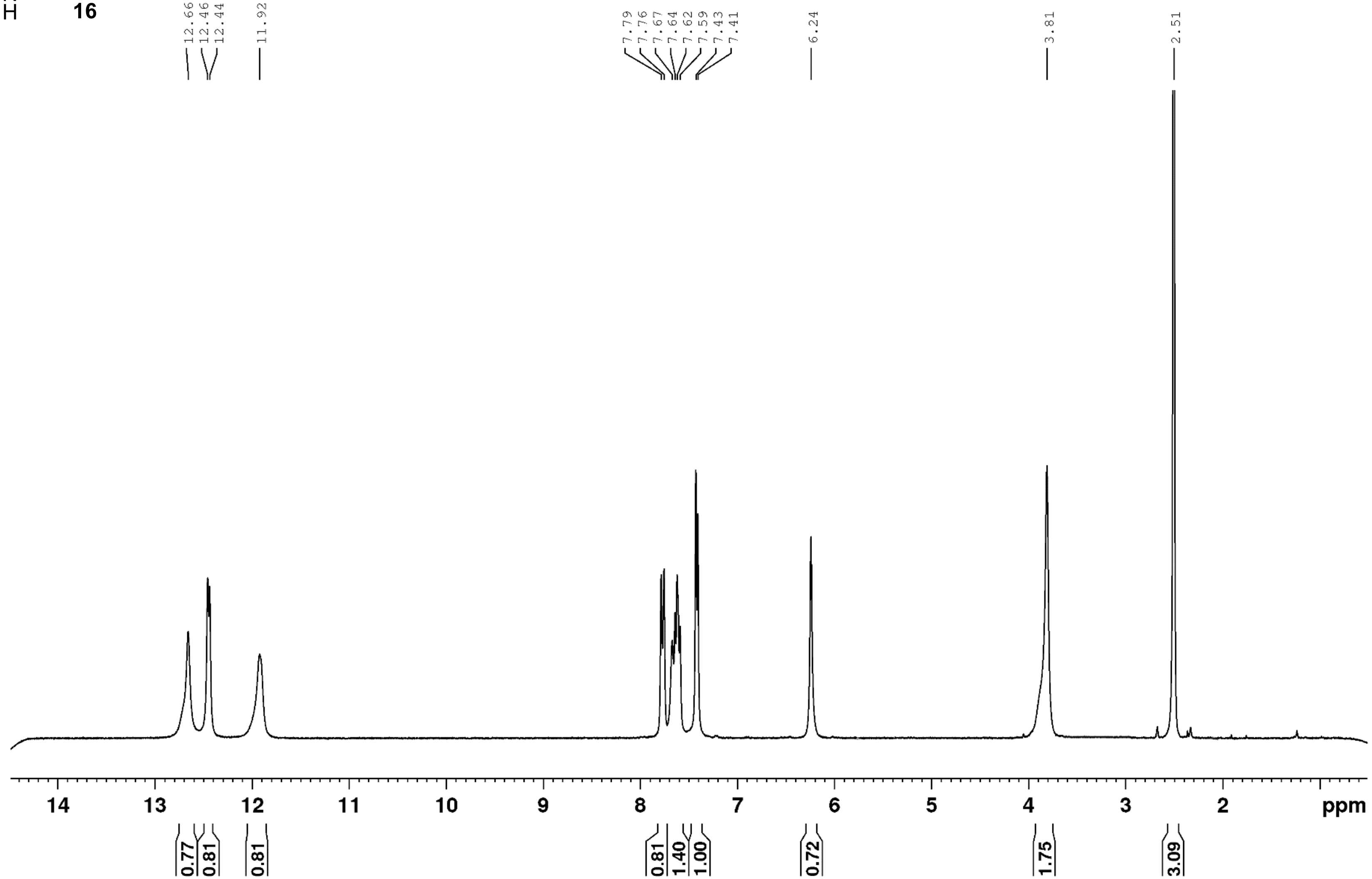
12.66
12.46
12.44
11.92
7.79
7.76
7.67
7.64
7.62
7.59
7.43
7.41
6.24
3.81
2.51
14 13 12 11 10 9 8 7 6 5 4 3 2 ppm
0.77
0.81
0.81
0.81
1.40
1.00
0.72
1.75
3.09

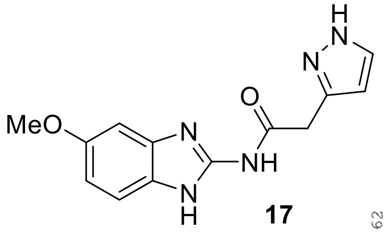
MeO
N
NH
N
H
O
N
N
H

17

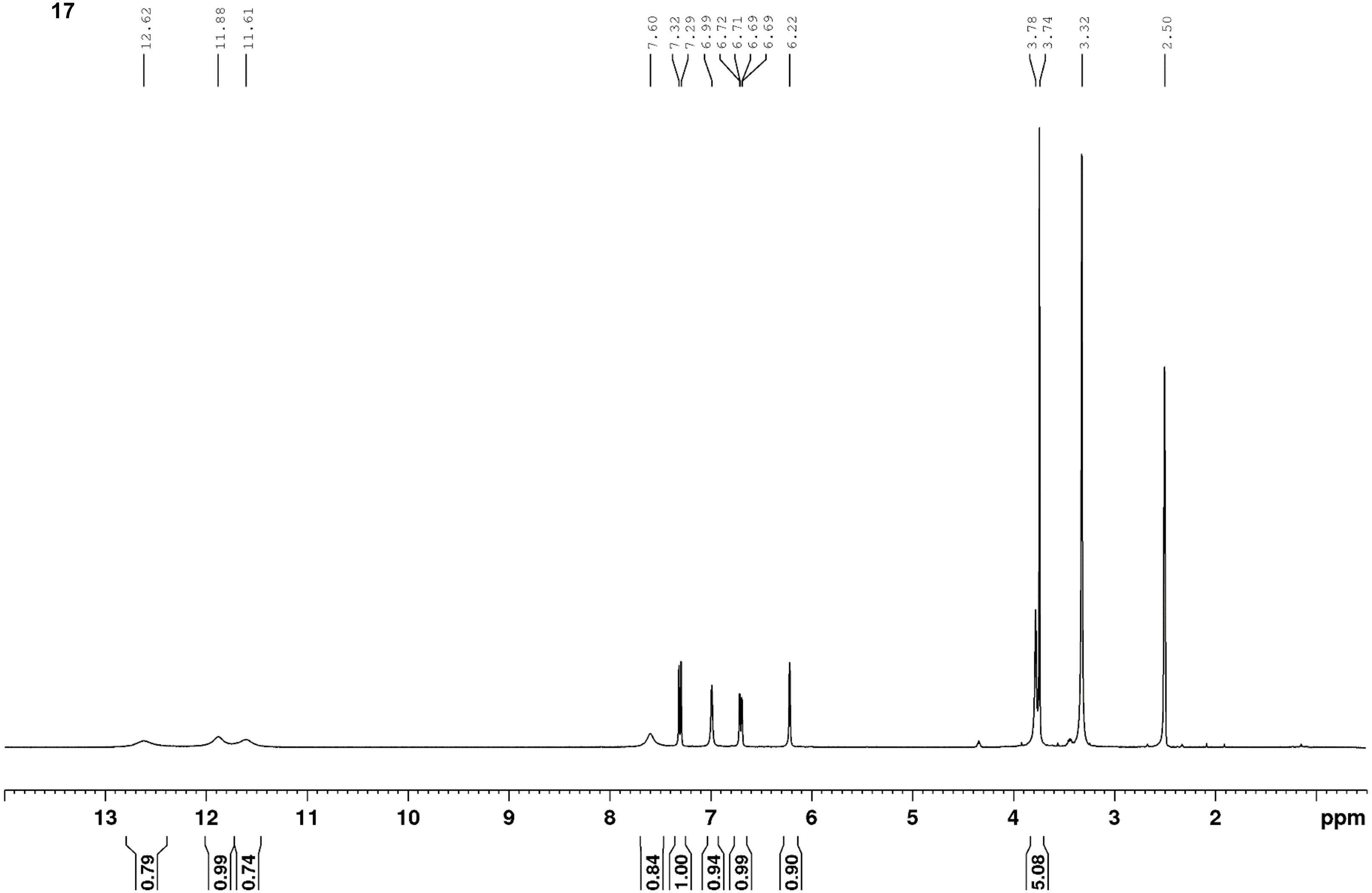
12.62
11.88
11.61
7.60
7.32
7.29
6.99
6.72
6.71
6.69
6.69
6.22
3.78
3.74
3.32
2.50
13
12
11
10
9
8
7
6
5
4
3
2
ppm
0.79
0.99
0.74
0.84
1.00
0.94
0.99
0.90
5.08

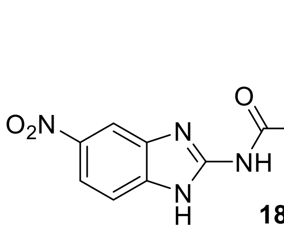

O2N
N
NH
N
H
O
N
N
H

18

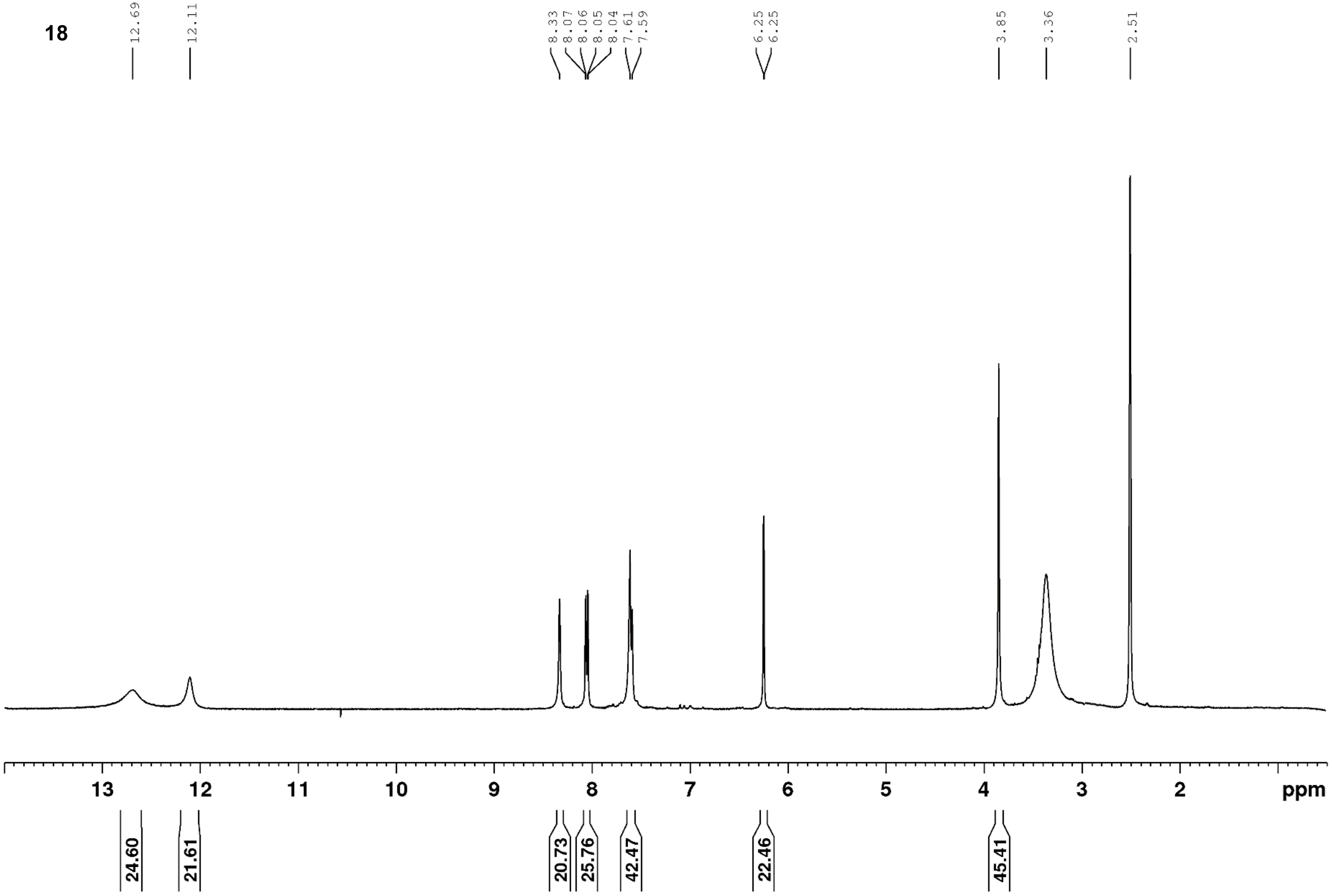

12.69
12.11
8.33
8.07
8.06
8.05
8.04
7.61
7.59
6.25
6.25
3.85
3.36
2.51
13
12
11
10
9
8
7
6
5
4
3
2
ppm
24.60
21.61
20.73
25.76
42.47
22.46
45.41

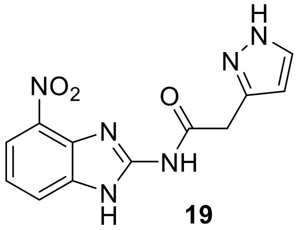
NO2
N
NH
O
N
N
H
N
H

19

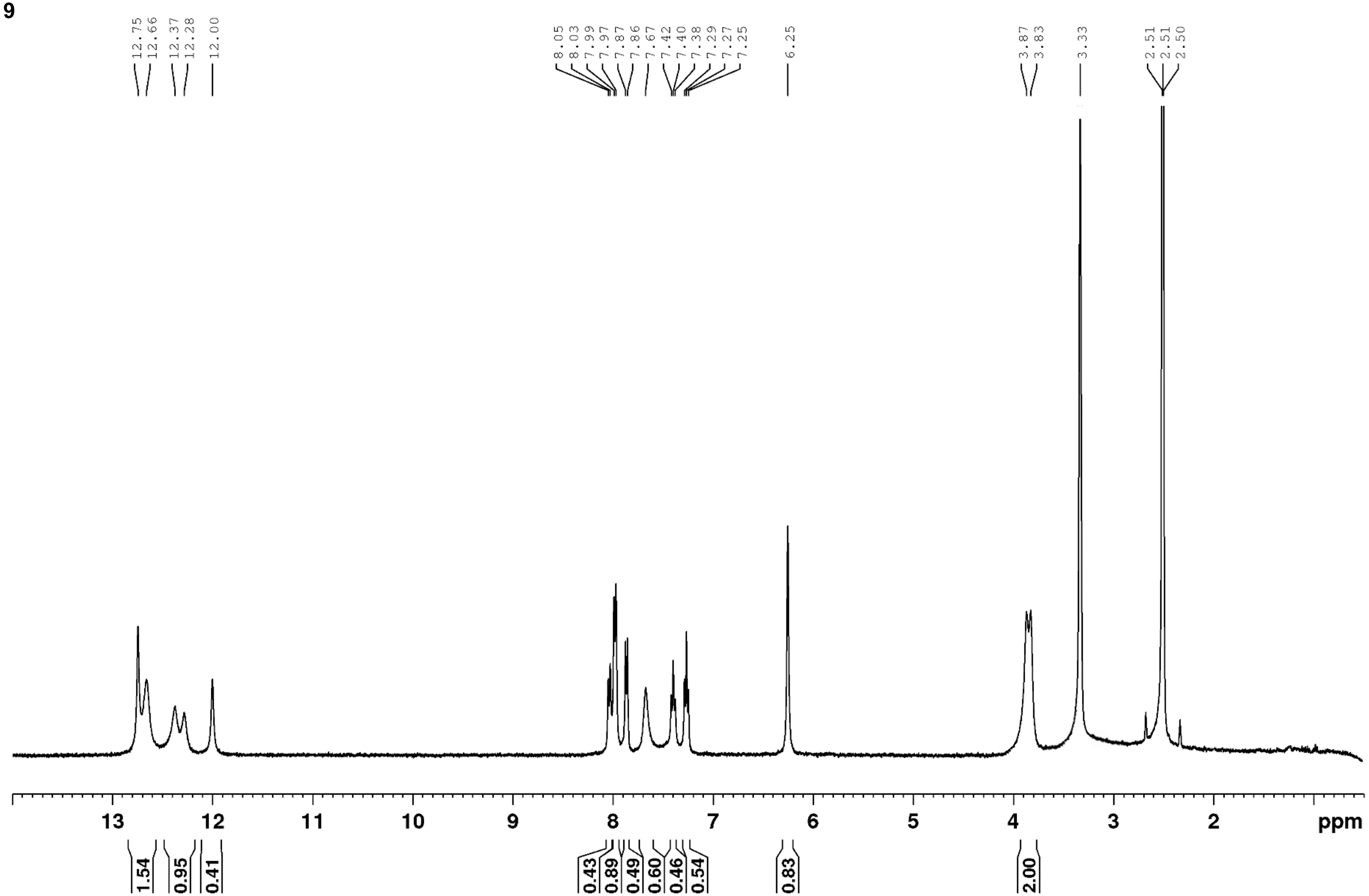
12.75
12.66
12.37
12.28
12.00
8.05
8.03
7.99
7.97
7.87
7.86
7.67
7.42
7.40
7.38
7.29
7.27
7.25
6.25
3.87
3.83
3.33
2.51
2.51
2.50
13
12
11
10
9
8
7
6
5
4
3
2
ppm
1.54
0.95
0.41
0.43
0.89
0.49
0.60
0.46
0.54
0.83
2.00

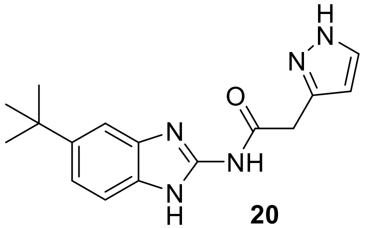

20

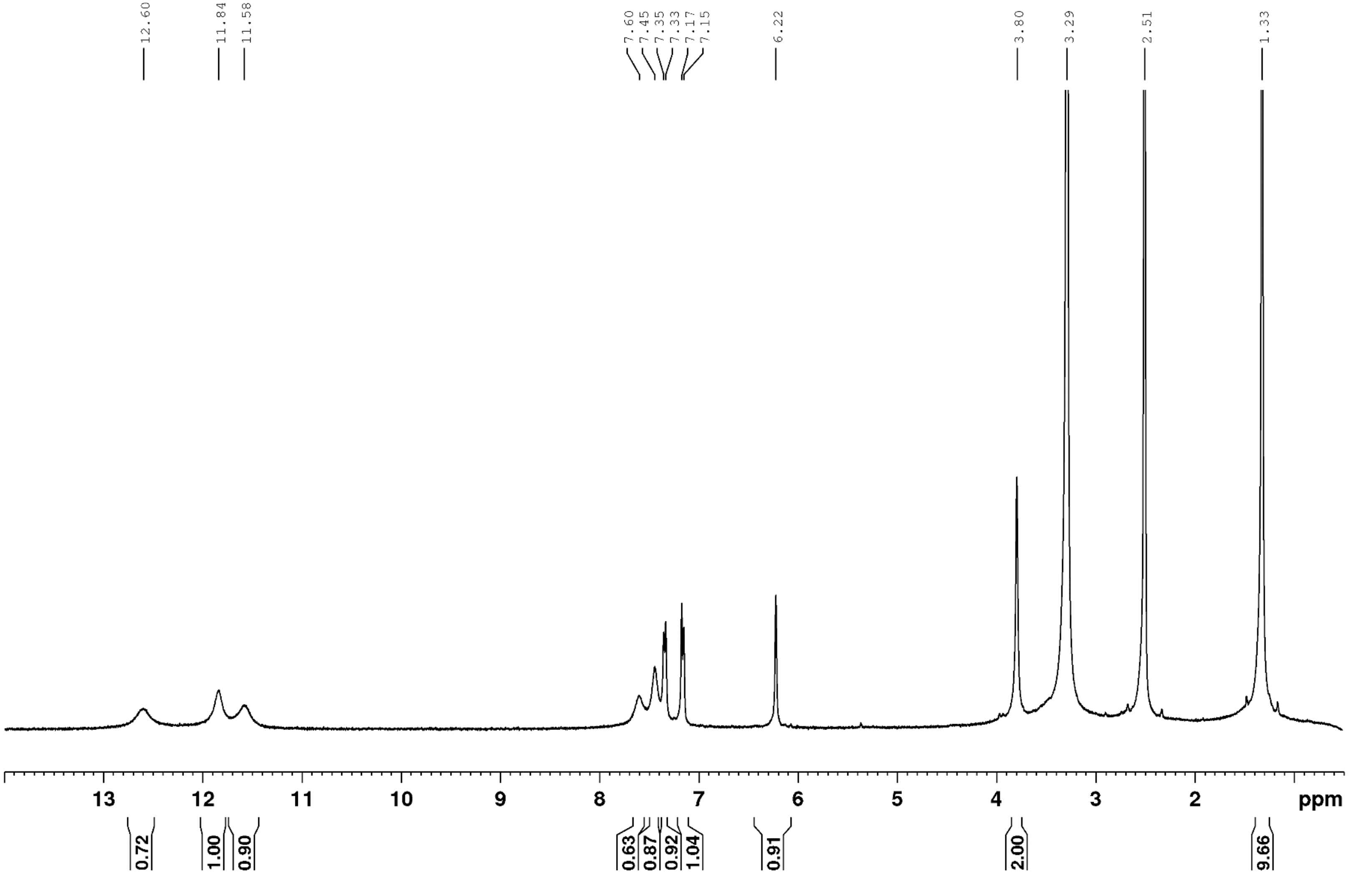
12.60
11.84
11.58
7.60
7.45
7.35
7.33
7.17
7.15
6.22
3.80
3.29
2.51
1.33
13 12 11 10 9 8 7 6 5 4 3 2 ppm
0.72
1.00
0.90
0.63
0.87
0.92
1.04
0.91
2.00
9.66

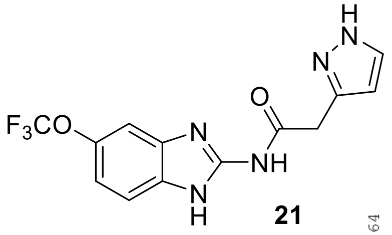
F3CO
N
NH
N
H
O
H
N
N

21

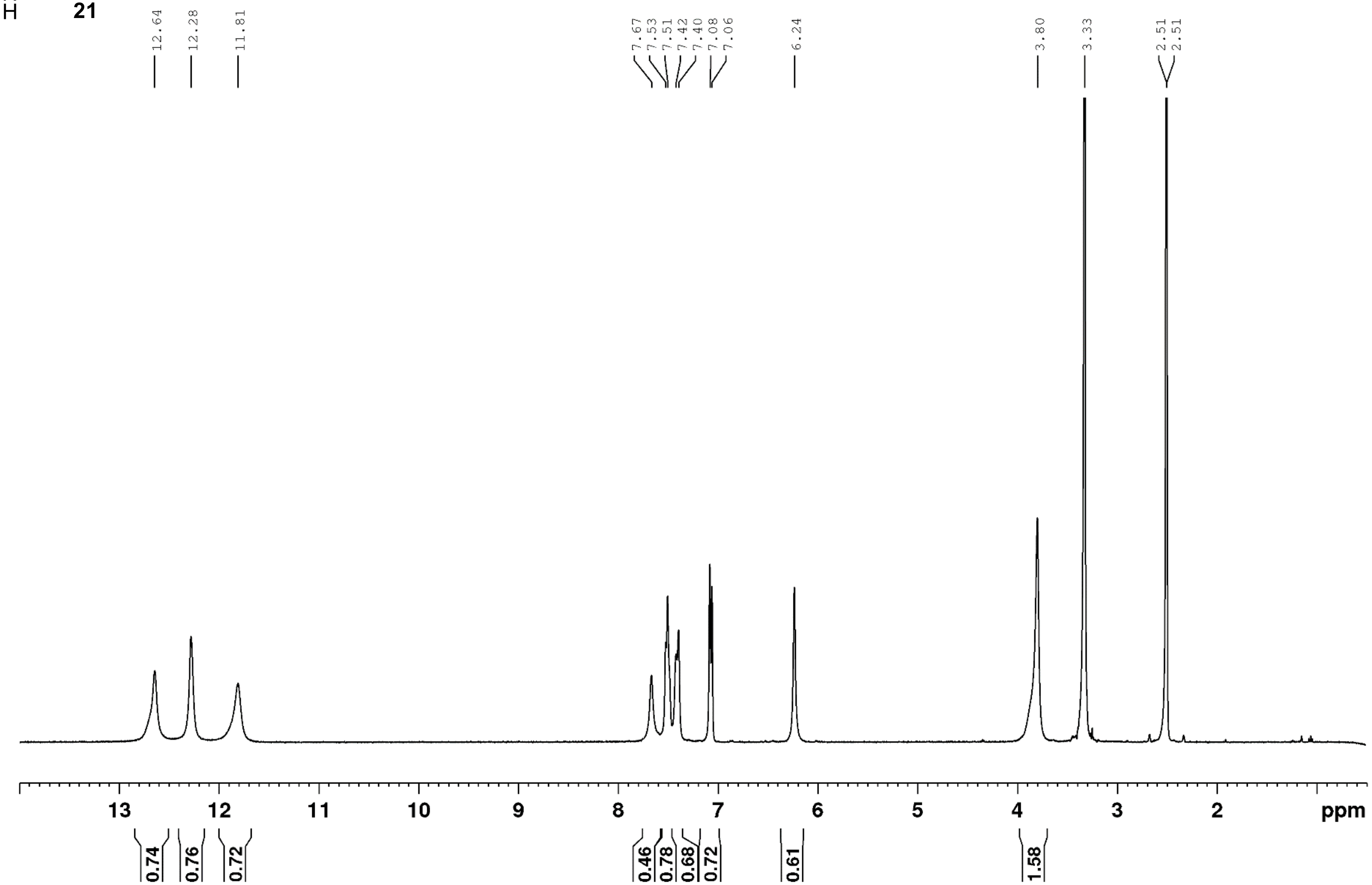
12.64
12.28
11.81
7.67
7.53
7.51
7.42
7.40
7.08
7.06
6.24
3.80
3.33
2.51
2.51
13
12
11
10
9
8
7
6
5
4
3
2
ppm
0.74
0.76
0.72
0.46
0.78
0.68
0.72
0.61
1.58

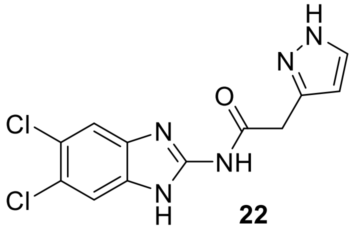

22

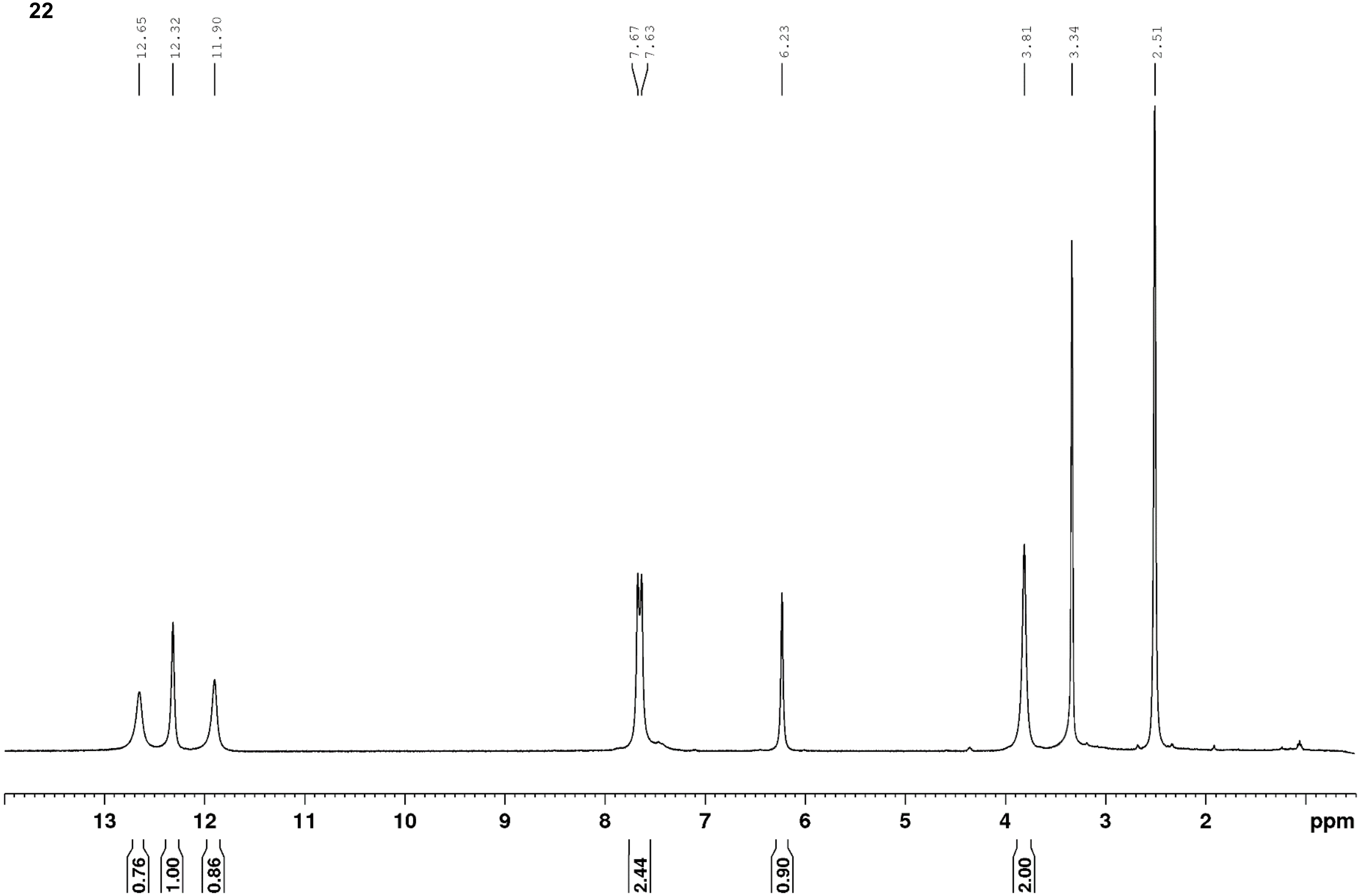

12.65
12.32
11.90
7.67
7.63
6.23
3.81
3.34
2.51
13
12
11
10
9
8
7
6
5
4
3
2
ppm
0.76
1.00
0.86
2.44
0.90
2.00

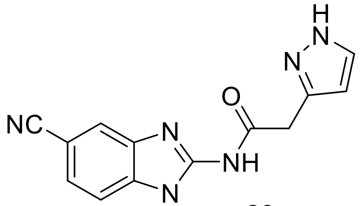
NC
N
NH
N
H
O
N
N
H

23

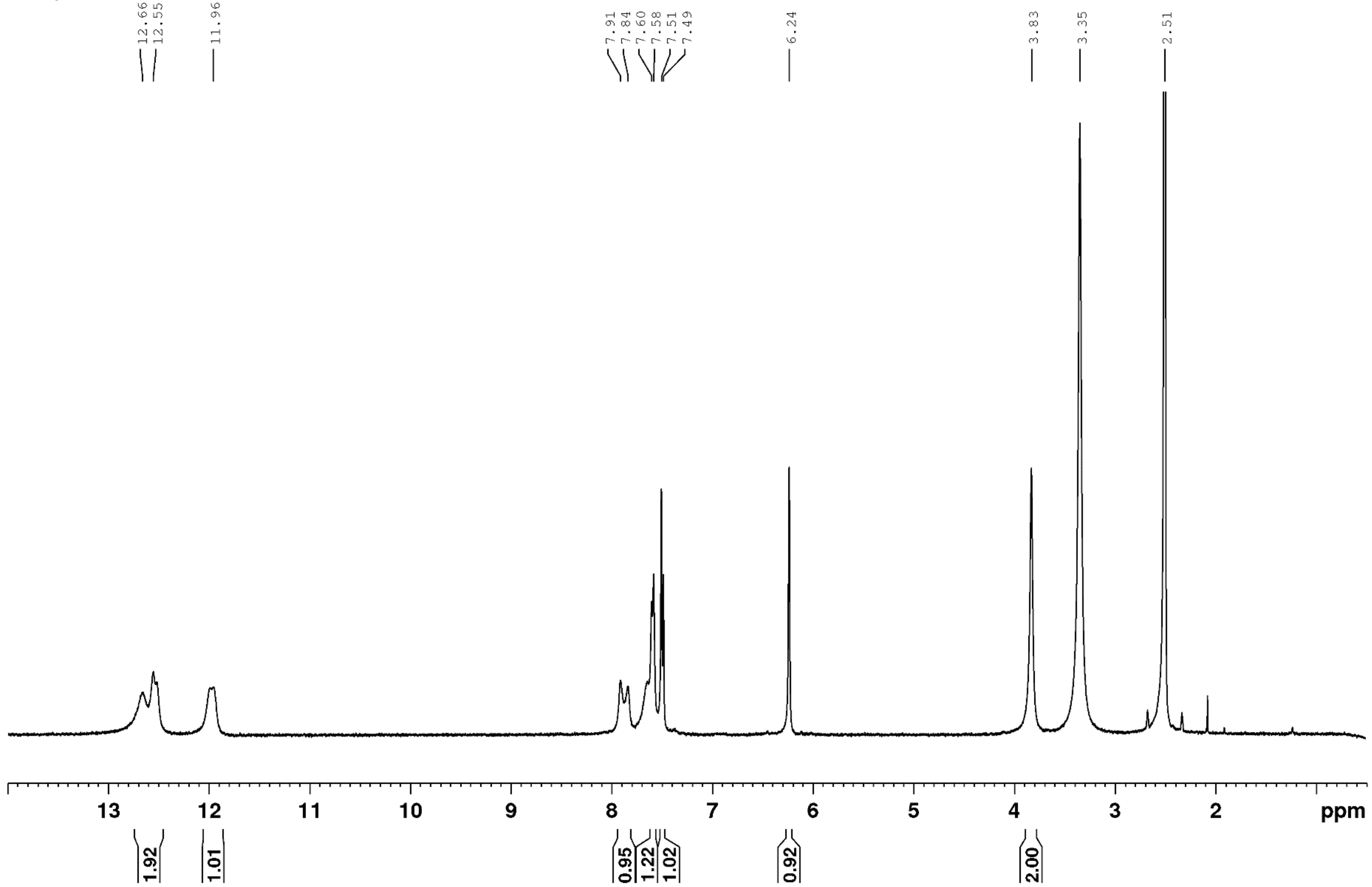
12.66
12.55
11.96
7.91
7.84
7.60
7.58
7.51
7.49
6.24
3.83
3.35
2.51
13 12 11 10 9 8 7 6 5 4 3 2 ppm
1.92
1.01
0.95
1.22
1.02
0.92
2.00

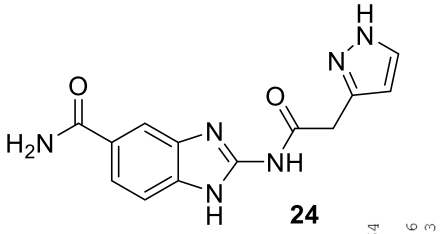

24

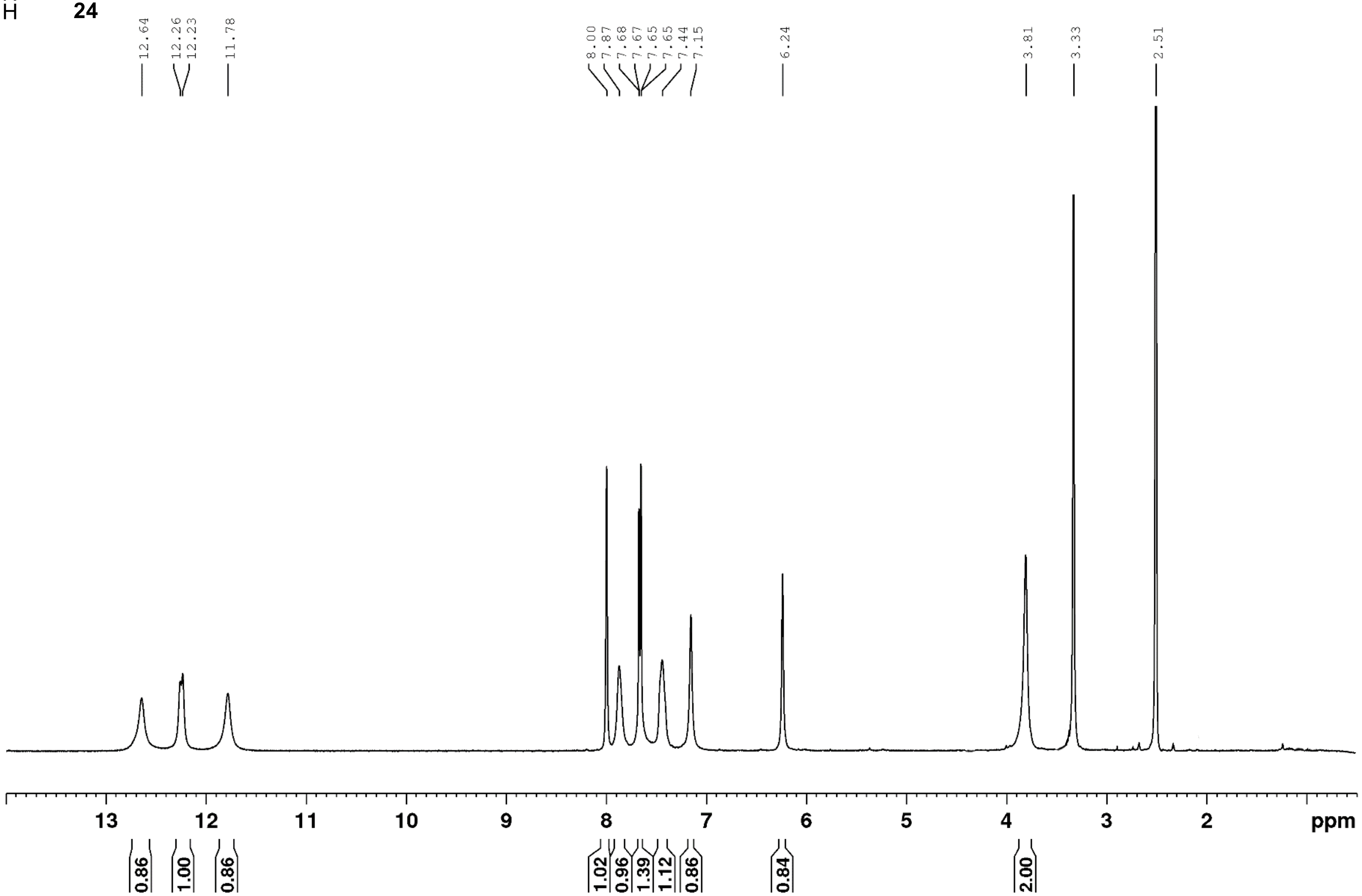
12.64
12.26
12.23
11.78
8.00
7.87
7.68
7.67
7.65
7.65
7.44
7.15
6.24
3.81
3.33
2.51
13 12 11 10 9 8 7 6 5 4 3 2 ppm
0.86
1.00
0.86
1.02
0.96
1.39
1.12
0.86
0.84
2.00

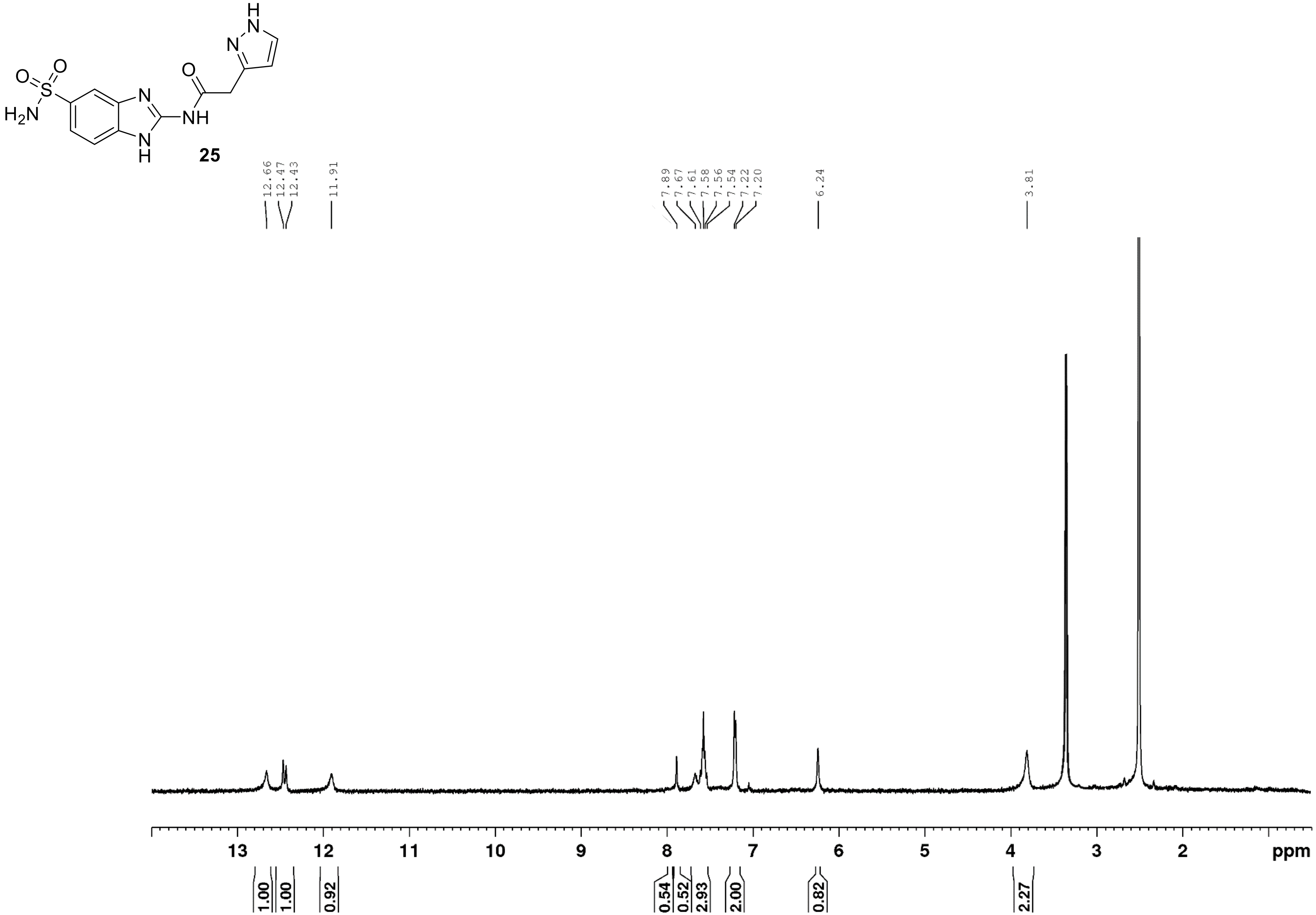
25
12.66
12.47
12.43
11.91
7.89
7.67
7.61
7.58
7.56
7.54
7.22
7.20
6.24
3.81
ppm
1.00
1.00
0.92
0.54
0.52
2.93
2.00
0.82
2.27

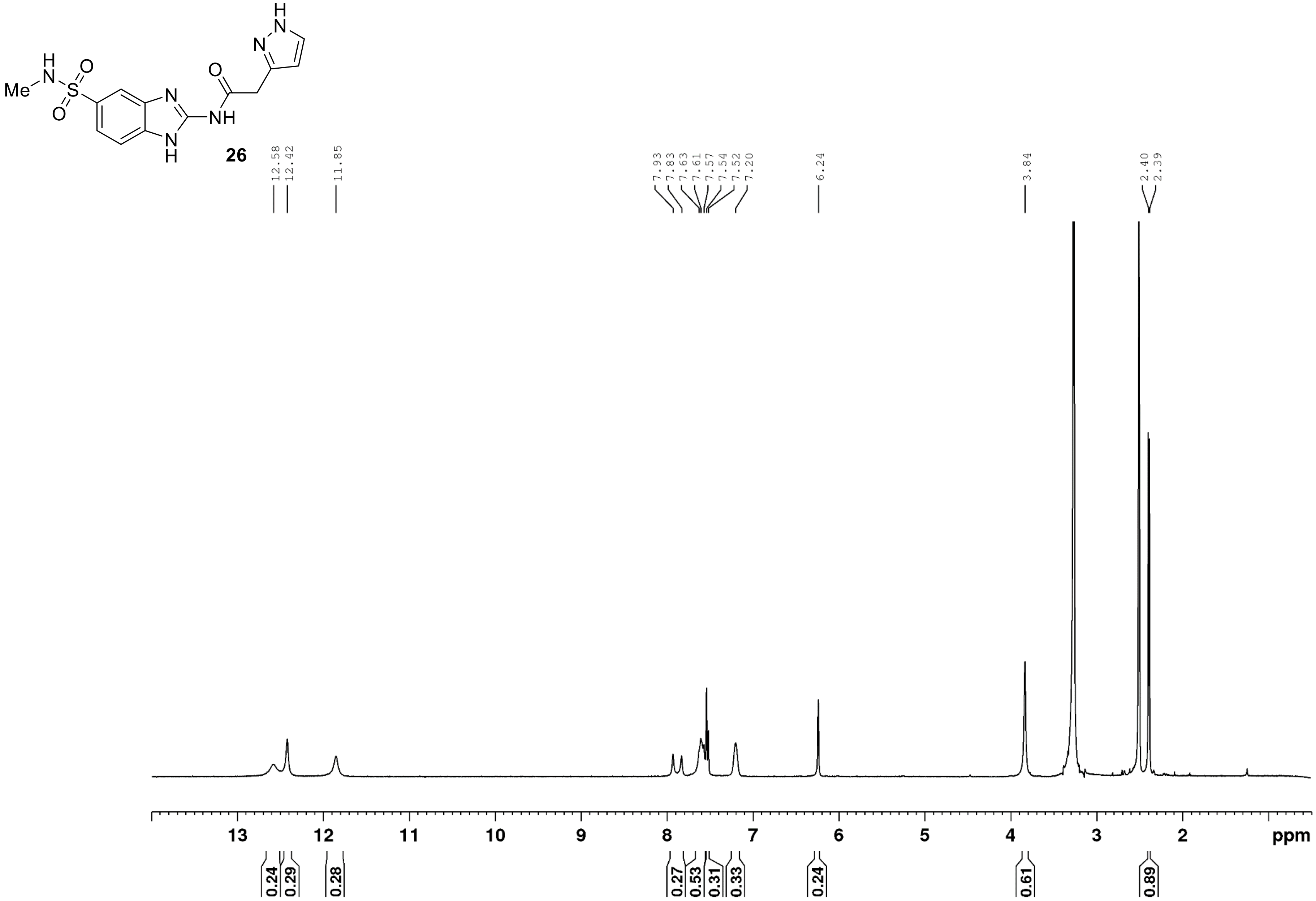
Me
H
N
S
O
O
N
NH
N
H
O
N
N
H
26
12.58
12.42
11.85
7.93
7.83
7.63
7.61
7.57
7.54
7.52
7.20
6.24
3.84
2.40
2.39
13
12
11
10
9
8
7
6
5
4
3
2
ppm
0.24
0.29
0.28
0.27
0.53
0.31
0.33
0.24
0.61
0.89

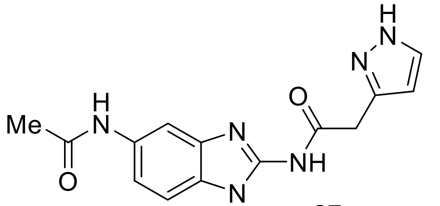

27

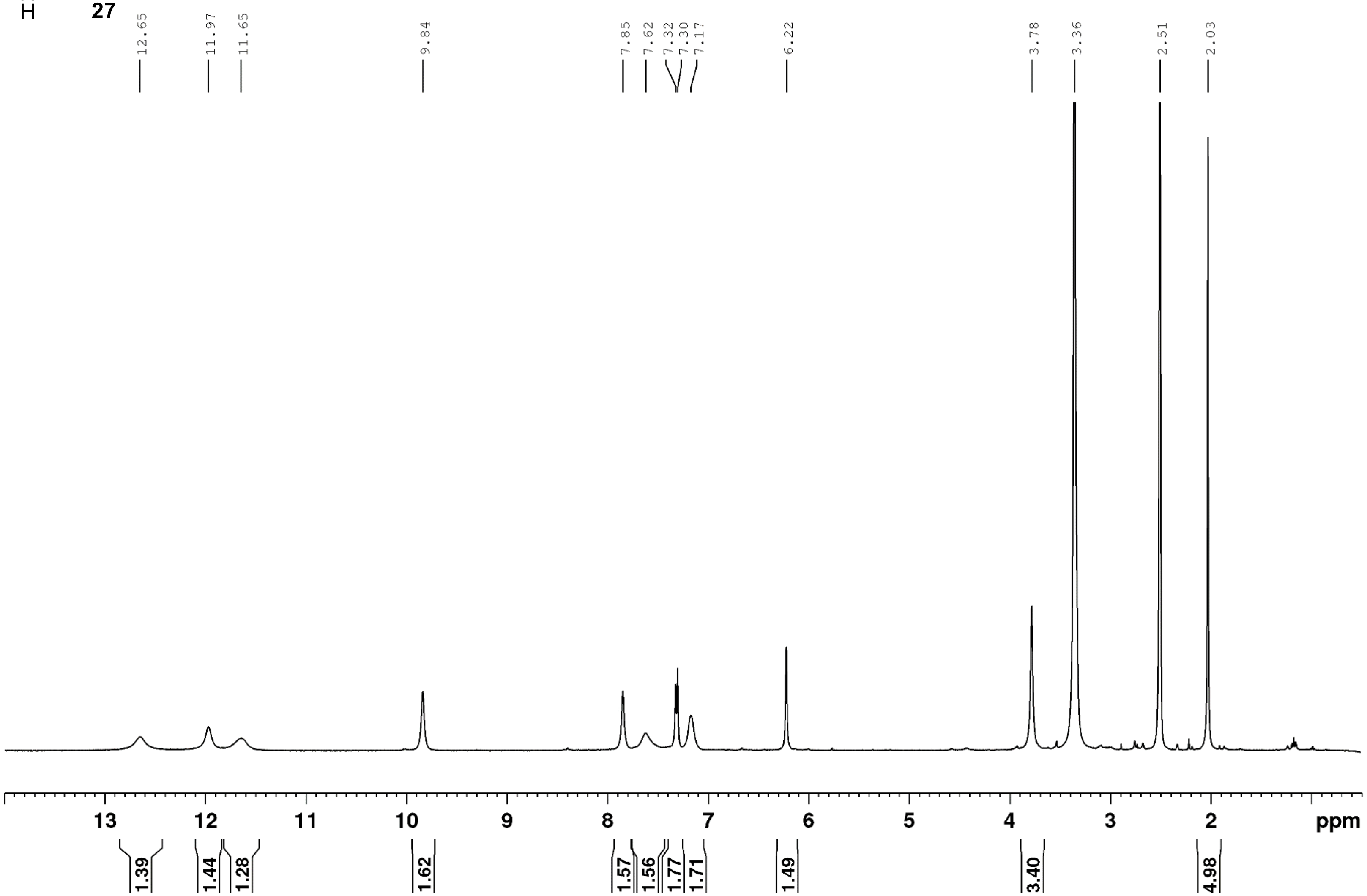
12.65
11.97
11.65
9.84
7.85
7.62
7.32
7.30
7.17
6.22
3.78
3.36
2.51
2.03
13
12
11
10
9
8
7
6
5
4
3
2
ppm
1.39
1.44
1.28
1.62
1.57
1.56
1.77
1.71
1.49
3.40
4.98

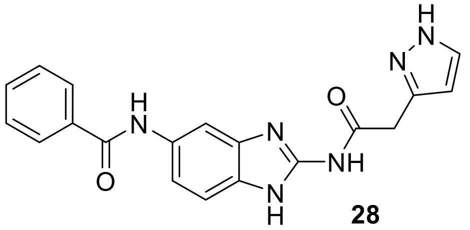

28

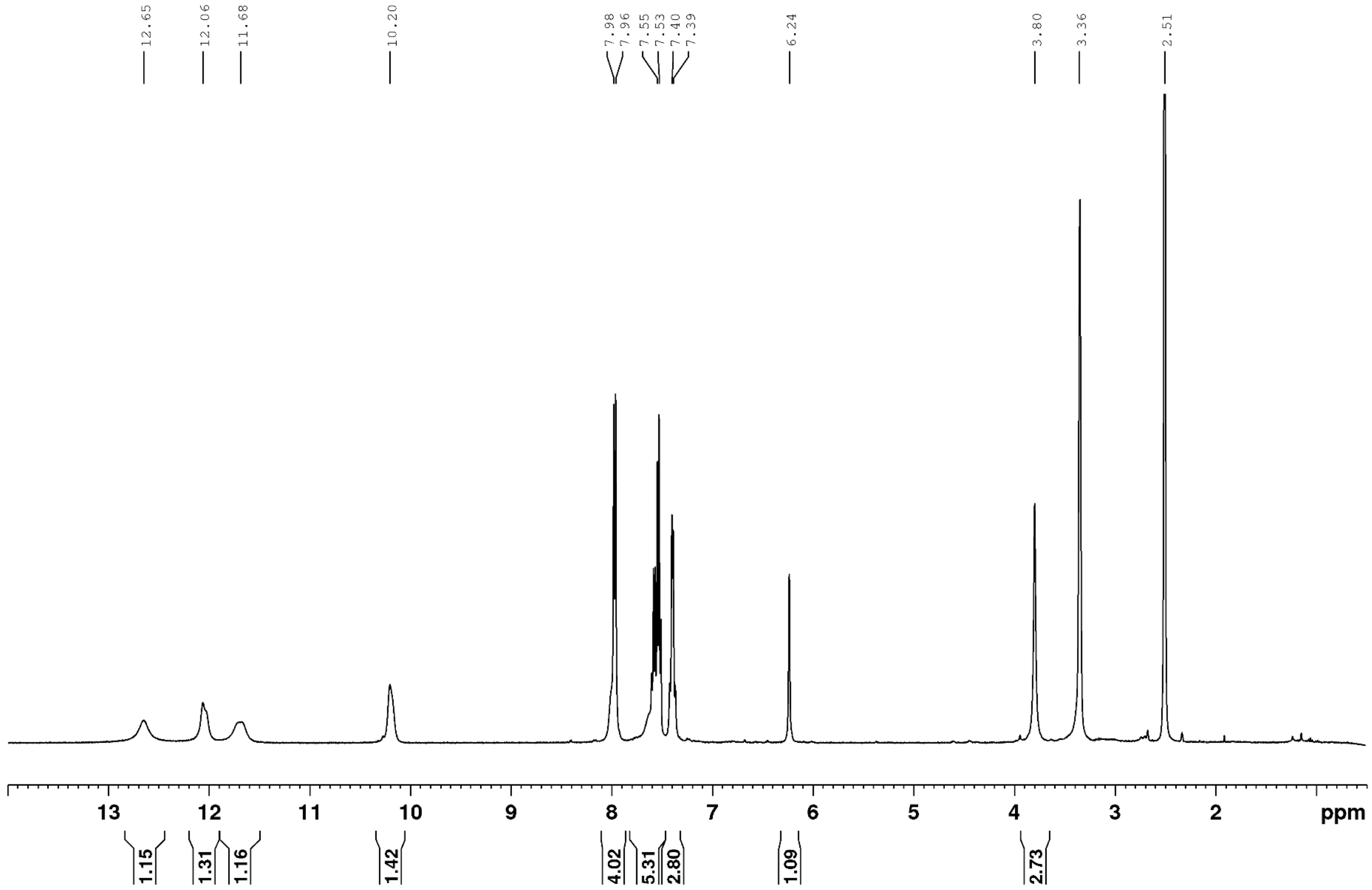
12.65
12.06
11.68
10.20
7.98
7.96
7.55
7.53
7.40
7.39
6.24
3.80
3.36
2.51
13
12
11
10
9
8
7
6
5
4
3
2
ppm
1.15
1.31
1.16
1.42
4.02
5.31
2.80
1.09
2.73

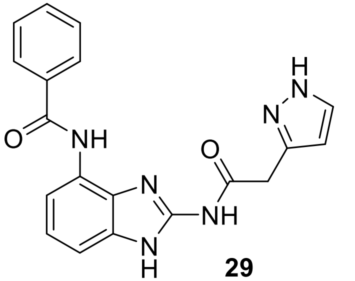
H
N
N
O
NH
O
N
NH
N
H

29

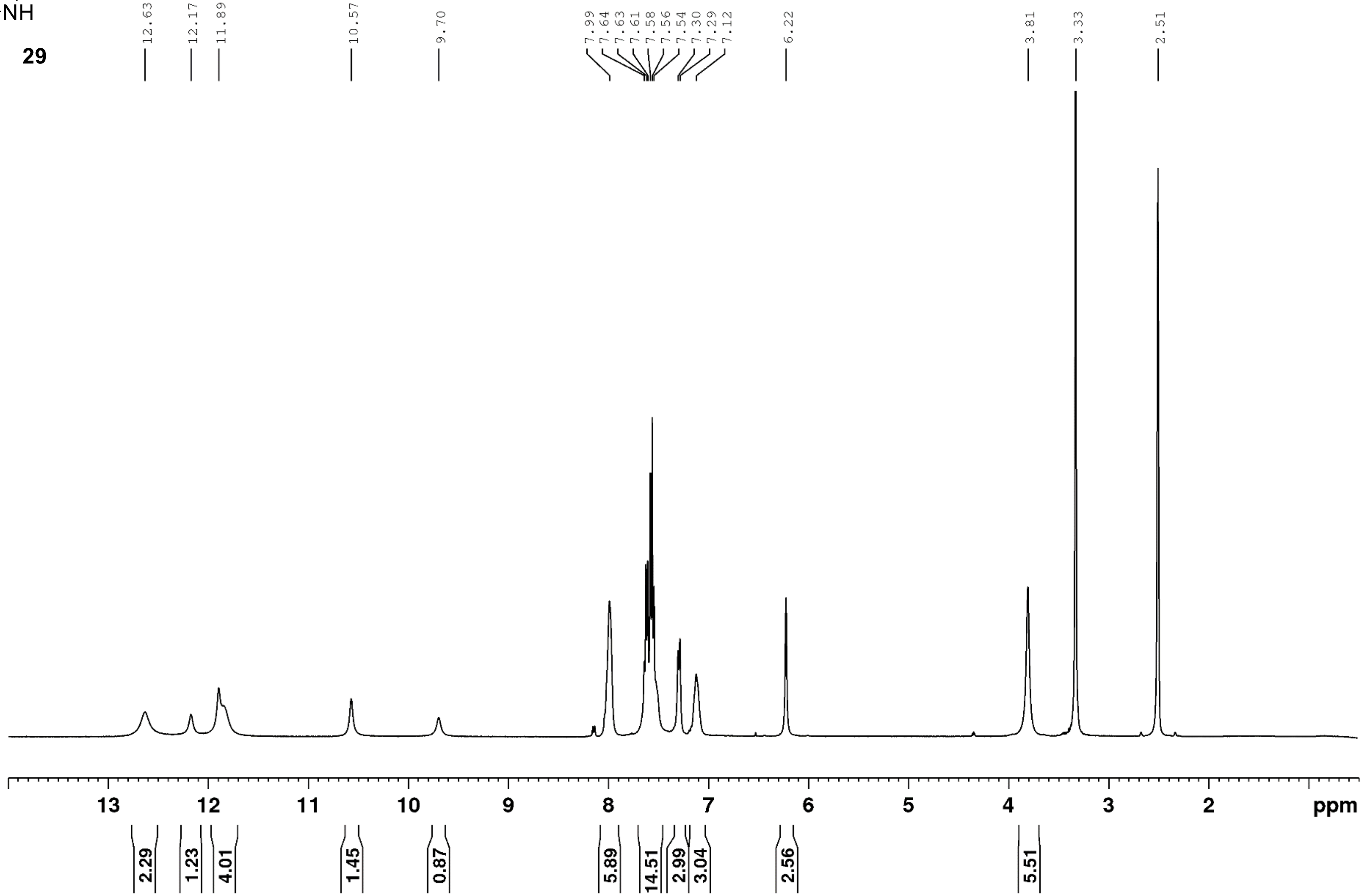
12.63
12.17
11.89
10.57
9.70
7.99
7.64
7.63
7.61
7.58
7.56
7.54
7.30
7.29
7.12
6.22
3.81
3.33
2.51
13
12
11
10
9
8
7
6
5
4
3
2
ppm
2.29
1.23
4.01
1.45
0.87
5.89
14.51
2.99
3.04
2.56
5.51

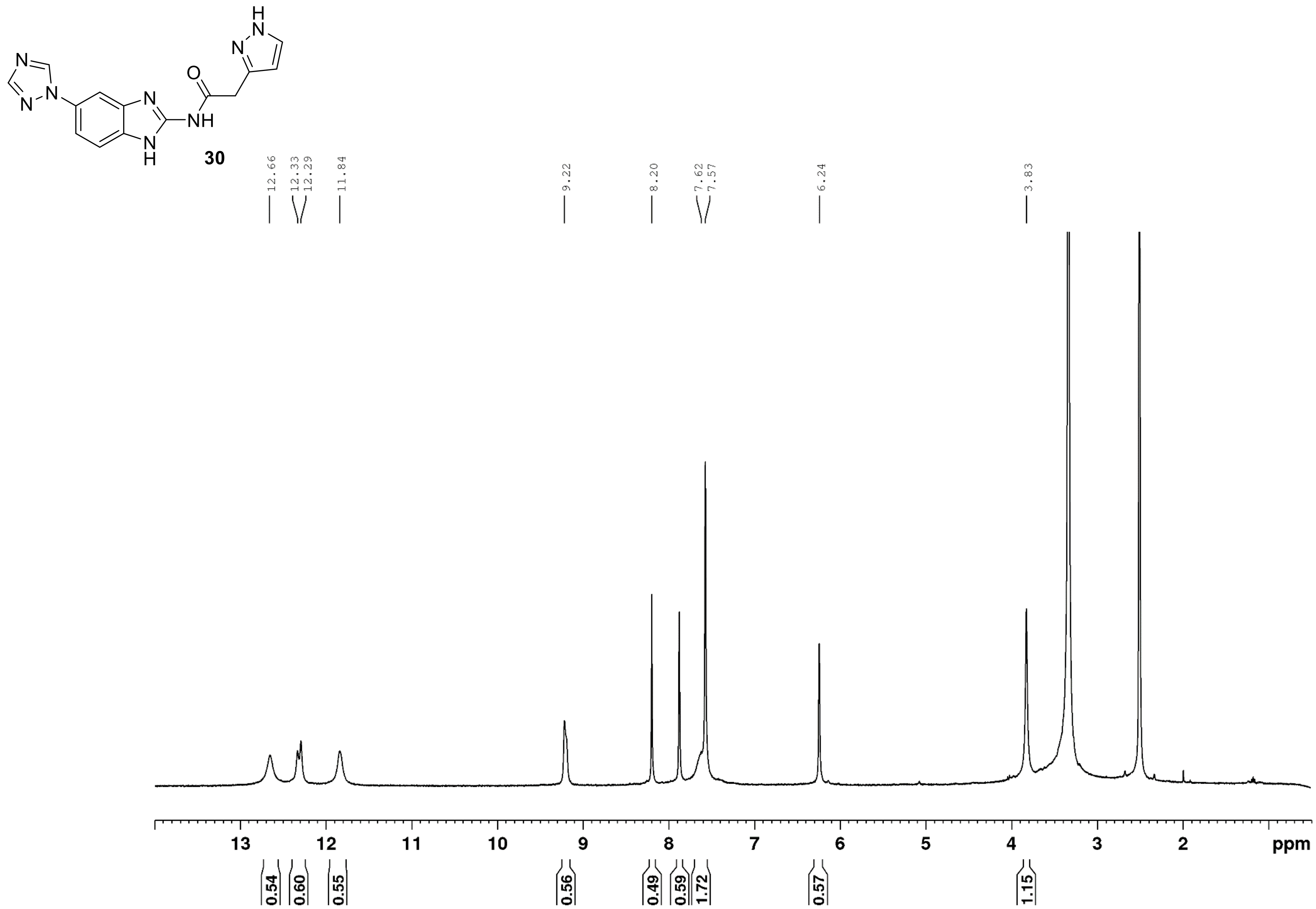

N
N
N
N
N
H
NH
O
N
N
H
30
12.66
12.33
12.29
11.84
9.22
8.20
7.62
7.57
6.24
3.83
13
12
11
10
9
8
7
6
5
4
3
2
ppm
0.54
0.60
0.55
0.56
0.49
0.59
1.72
0.57
1.15

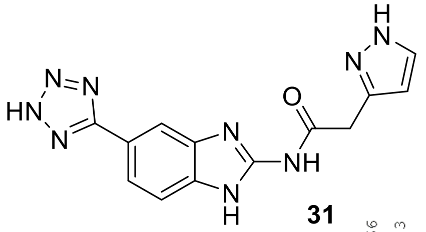

31

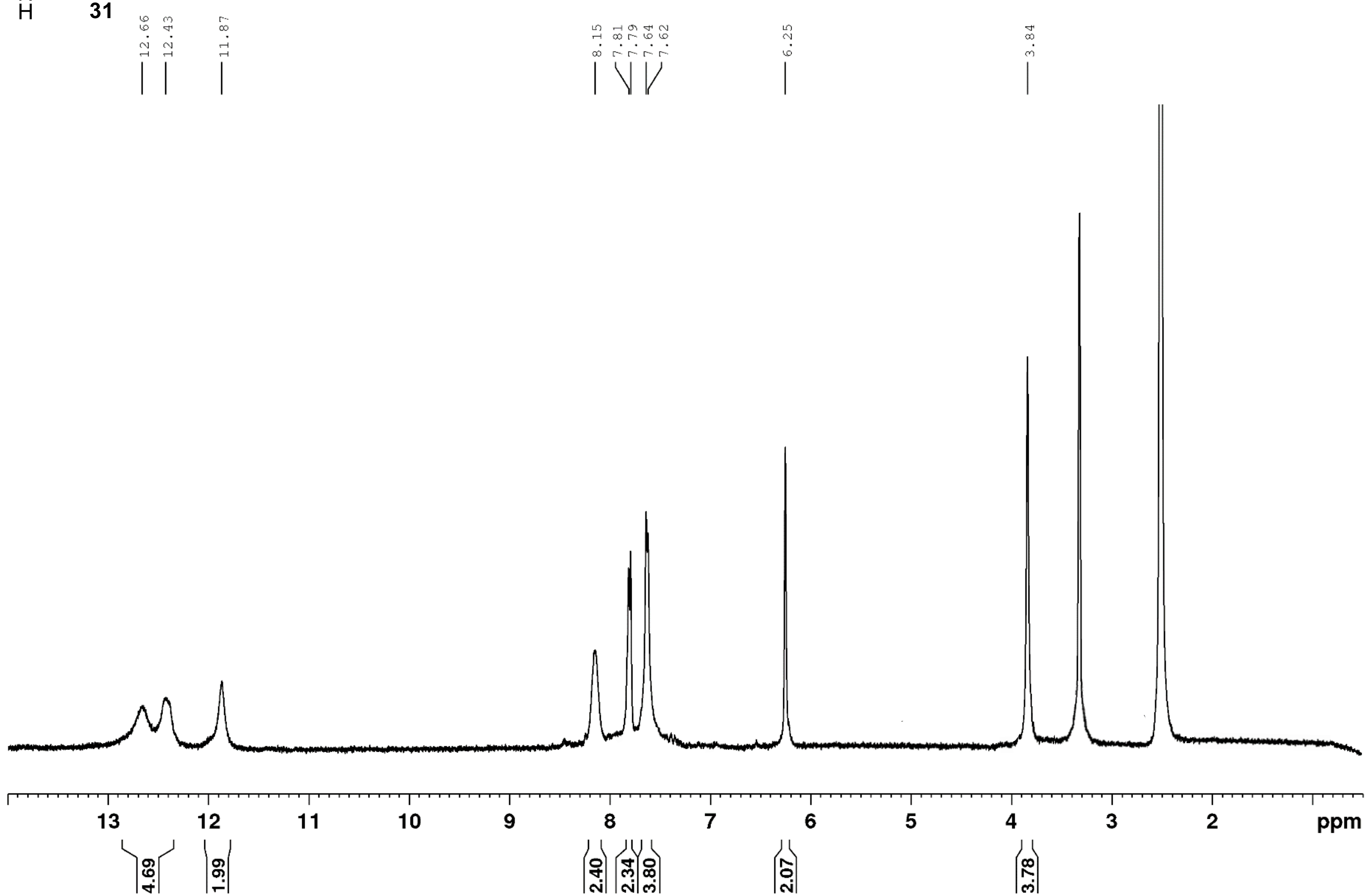

12.66
12.43
11.87
8.15
7.81
7.79
7.64
7.62
6.25
3.84
13
12
11
10
9
8
7
6
5
4
3
2
ppm
4.69
1.99
2.40
2.34
3.80
2.07
3.78

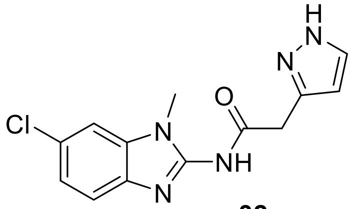
Cl
N
NH
O
N
N
H

32

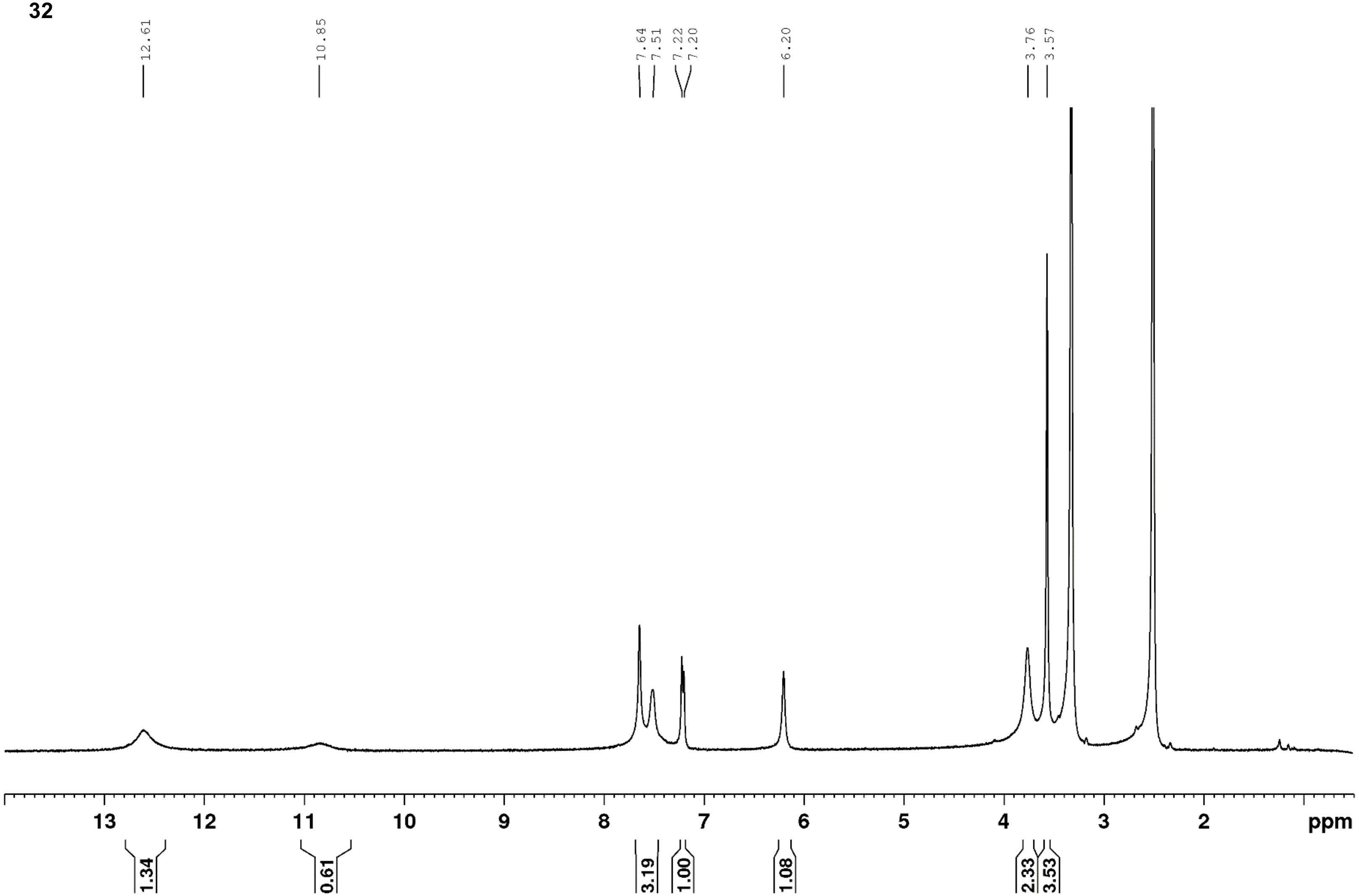
12.61
10.85
7.64
7.51
7.22
7.20
6.20
3.76
3.57
13
12
11
10
9
8
7
6
5
4
3
2
ppm
1.34
0.61
3.19
1.00
1.08
2.33
3.53
